# Optimization of the extraction process and *in vitro* antioxidant capacity analysis of selenium-containing proteins from *Cynanchum thesioides*

**DOI:** 10.7717/peerj.20998

**Published:** 2026-04-15

**Authors:** Yan Men, Xu Han, Xiumei Huang, Xiaoyan Zhang, Pengchao Wei, Zhongren Yang, Fenglan Zhang

**Affiliations:** 1 Inner Mongolia Agricultural University, Hohhot, China; 2 Key Laboratory of Agriculture and Animal Husbandry Big Data Research and Application, Hohhot, Saihan district, China

**Keywords:** *Cynanchum thesioides*, Se-containing proteins, *In vitro* antioxidant activity, Response surface methodology, Selenium biofortification, Extraction optimization

## Abstract

Current research lacks established approaches to concurrently achieve a high extraction yield with preserved bioactivity and to synergistically enhance plant selenium content and protein quality through agronomic practices. This study tested whether alkaline extraction maximizes selenoprotein yield without compromising antioxidant activity and whether foliar selenium application increases both fruit selenium content and the antioxidant capacity of extracted proteins. The fruits of the selenium-hyperaccumulator plant * Cynanchum thesioides* and its variant were utilized as experimental materials to systematically explore the differences in nutritional components and selenium (Se) content among various fruit types. The extraction conditions for Se-containing proteins using five distinct strategies were optimized *via* Response Surface Methodology (RSM), focusing on the extraction mechanism of alkaline extraction and the *in vitro* antioxidant activities of the derived Se-containing proteins. The findings revealed that the large fruits of the variant contained the highest selenium content (1.06 ± 0.02 mg/kg, *n* = 3). Under the optimal alkaline extraction conditions (solid-to-liquid ratio of 1:22.7, temperature of 44.62 °C, and NaOH concentration of 0.243 mol/L), the extraction yield of Se-containing proteins was 5.9 3 ± 0.15 mg/g (*n* = 3), significantly higher than those obtained by other methods (*P* < 0.05). Foliar application of selenomethionine (2 mg/L) significantly enhanced the selenium accumulation in the fruits to 1.92 ± 0.11 mg/kg (*n* = 3) and improved the antioxidant efficacy of the alkaline -extracted proteins. At a concentration of 5 mg/mL, the extract demonstrated strong antioxidant activity, with scavenging rates of 53.74% for superoxide anion (O_2_⋅−), 68.73% for hydroxyl radical (⋅OH), and 62.58% for DPPH radical (DPPH⋅), along with a ferric reducing antioxidant power (FRAP) value of 1.33 ± 0.05 AU. This study offers a theoretical foundation and application prospects for the development of *C. thesioides* Se-containing proteins as antioxidant dietary components or functional food ingredients, encapsulating both extraction mechanisms and the functional activities.

## Introduction

Selenium (Se) is an essential trace element for humans and animals and benefits plant health. Since its recognition as an essential trace element, its diverse physiological functions have continued to attract significant scientific interest (Hatfield et al., 2014). Agricultural practices such as selenium fertilization and selenium-enriched feed effectively increase selenium levels in crops, enhancing their stress resistance and nutritional value (White, 2016). Selenium deficiency remains a global issue and is particularly severe in China, where approximately 72% of the land is selenium-deficient or low in selenium, posing health risks to nearly 700 million people (Tan et al., 2002).

In plants, selenium exists primarily in organic forms, including low-molecular-weight compounds such as selenomethionine and selenocysteine, as well as biological macromolecules like selenoproteins and selenopolysaccharides (Pyrzynska & Sentkowska, 2021). Selenoproteins, the main carriers of selenium in plants, are a unique class of functional proteins in which selenium is covalently incorporated as selenocysteine into polypeptide chains (Labunskyy, Hatfield & Gladyshev, 2014). Total selenium encompasses all selenium species, with protein-bound selenium (primarily in the form of selenoproteins) representing the key bioactive form. This study focuses on selenoprotein-rich crude extracts, as their well-defined bioactivities enable the systematic optimization of the extraction process to balance yield and functional preservation—a central objective in product development. The potential value of other selenium forms warrants further investigation. The primary extraction methods for Se-containing proteins include solvent extraction, chromatographic column separation, and various biological techniques (Luo et al., 2025). Of these methods, solvent extraction is particularly favored due to its simplicity and cost-effectiveness, employing various approaches such as water, salt, acid, alkaline, and organic solvent extraction (Gong et al., 2024). Beyond their roles in storage and transport, plant-derived selenoproteins are highly effective in scavenging free radicals, ameliorating liver damage, and reducing radiation-induced harm, thereby offering superior bioprotective effects compared to their inorganic counterparts (Zhang et al., 2004).

*Cynanchum thesioides* (Freyn) K. Schum and its variant, *C. thesioides* var. *australe*, are perennial xerophytic plants belonging to the genus *Cynanchum* in the Apocynaceae family. Phytochemical investigations have revealed that *C. thesioides* contains a diversity of bioactive constituents, including alkaloids, flavonoids, terpenoids, and organic acids. Notably, unique compounds like succinic acid have been successfully isolated from its roots, stems, and leaves (Yuan & Zuo, 1992). As a traditional wild vegetable, the fruit of *C. thesioides* boasts a rich nutritional profile, encompassing minerals, vitamins, soluble proteins, and lipids, and offers a distinctive flavor and varied culinary applications. Critically, *C. thesioides* has been identified as a naturally selenium-enriched plant, displaying a selenium accumulation capacity that far surpasses that of typical vegetable varieties, thus underscoring its significant potential for selenium biofortification and its development value (Han, 2023).

Despite the recognized selenium-enrichment capabilities of *C. thesioides*, there has been no reported systematic comparison of nutritional components *versus* selenium content among its different fruit types, nor any reported cross-method optimization of selenoprotein extraction strategies. This study aims to fill this gap, with specific objectives including: systematically comparing the nutrition and selenium content among different fruit types; determining the optimal strategy for selenoprotein extraction through multi-method optimization; and evaluating the *in vitro* antioxidant activity of the obtained selenoproteins. The core scientific question this study aims to address is: For the fruits of *C. thesioides*, which extraction method can maximize selenoprotein yield while best preserving its antioxidant activity, and how does foliar selenium application influence this relationship? We propose the following a priori hypotheses: Alkaline extraction is expected to achieve the highest selenoprotein yield due to its ability to disrupt cell walls and denature non-target proteins, and its extracts are predicted to retain high antioxidant activity. Furthermore, we hypothesize that moderate foliar selenium application will improve both total fruit selenium content and the *in vitro* antioxidant capacity of the protein extracts, whereas excessive selenium application may lead to adverse effects. To balance optimization efficiency and model reliability, this study adopted a two-stage strategy: key variables were first screened through single-factor experiments, followed by RSM to analyze interaction effects and determine the globally optimal process. The findings of this study provide direct guidance for process selection in developing *C. thesioides-* based selenium-enriched functional foods. Furthermore, the evaluation of extreme pH extraction conditions (*e.g.*, strong alkali) provides important references for the selection of equipment materials and product safety assessments.

## Materials & Methods

### Experimental plan overview

This study aimed to enhance the quality and functionality of *C. thesioides* fruits *via* foliar selenium application. A systematic four-step procedure was implemented: material preparation, component analysis, process optimization, and efficacy validation. First, fruit powder was prepared, and its basic nutritional components alongside baseline selenium content were determined. Second, single-factor experiments were used for preliminary screening and determining appropriate ranges for key variables, while Box-Behnken design (RSM) was employed to model complex interactions and precisely locate the global optimum. Five extraction methods (aqueous, alkaline, acidic, saline, and organic solvent) were optimized using single-factor tests and RSM to maximize the extraction yield of selenoproteins. Subsequently, different concentrations of selenium fertilizer were foliar-sprayed during the flowering stage to produce selenium-enriched fruits. Finally, the total selenium content was quantified, and protein extracts were obtained using the optimized protocol. These extracts were then comprehensively assessed for *in vitro* antioxidant activity, including DPPH radical scavenging capacity, hydroxyl radical scavenging capacity, ferric reducing antioxidant power (FRAP), and superoxide anion scavenging capacity. This integrated approach elucidates the correlations among selenium fertilization, selenium enrichment, protein extraction efficiency, and the enhancement of antioxidant activity. The complete workflow is illustrated in Fig. S1 (Supplementary Material). All experiments were conducted in the Horticulture and Plant Protection Laboratory at Inner Mongolia Agricultural University, in strict compliance with the institutional guidelines.

### Experimental materials and processing

In August 2022, fruit samples were collected from the teaching experimental base located at Inner Mongolia Agricultural University in Hohhot, Inner Mongolia Autonomous Region. The GPS coordinates of the sampling site are (40.81°N, 111.71°E). The climate type is temperate continental monsoon climate, with an average annual temperature of about 8.3 °C and an annual precipitation of approximately 392.2 mm. The soil type is chestnut soil, with a pH of about 7.5–8.5 (slightly alkaline). Specimens of *Cynanchum thesioides* (Freyn) K. Schum and its variant, *C. thesioides* var. *australe*, that were approximately 2 years old and exhibited healthy, uniform growth were collected. Mature, disease-free fruits exhibiting natural growth were harvested. The fruits were collected approximately 45–50 days after flowering, at which point they were fully developed but had not yet dehisced. These fruits were classified based on morphological characteristics into three categories: large fruits (length ≥ five cm, width ≥ 1.5 cm), medium fruits (either length ≥ five cm and width <1.5 cm, or length <five cm and width ≥ 1.5 cm), and small fruits (both length <five cm and width <1.5 cm). Following collection, the samples were cleansed with deionized water, dried in an oven at 60 °C to constant weight (typically achieved within 48 h), finely ground, sifted through an 80-mesh sieve, hermetically sealed, and stored at −18 °C until further analysis. Each combination of size category and species included three independent biological replicates (*n* = 3). All measurements were performed with three technical replicates, and the data were averaged for subsequent statistical analysis. All plant materials were identified by Professor Quansheng Chen, and the voucher specimen is deposited in the Herbarium, Institute of Botany, Chinese Academy of Sciences (PE) with the accession number PE02034772.

### Nutritional component analysis

The moisture content was determined by the oven-drying method (Jain, 2024). Vitamin C levels were quantified through ultraviolet spectrophotometry (Rahman Khan et al., 2006). Total soluble sugars were determined employing the anthrone-sulfuric acid technique (Jain, 2024). Crude fiber was quantified by colorimetry (Gao, 2006). Crude fat extraction was performed using the Soxhlet method (Jain, 2024). Protein concentration was determined using the Coomassie Brilliant Blue G-250 method using bovine serum albumin (BSA) as the standard (0–100 µg/mL) (Rahman Khan et al., 2006). Selenium content was measured using graphite furnace atomic absorption spectrometry (GFAAS, PerkinElmer PinAAcle 900T). The instrument was operated at an analytical wavelength of 196.0 nm, with nickel nitrate applied as a matrix modifier. All analytical procedures demonstrated satisfactory quality control, with calibration curves showing good linearity (R^2^ > 0.9991), spike recoveries ranging from 95% to 105%, and relative deviations of parallel samples below 8%, confirming the reliability of the data (Ajtony et al., 2005). Each measurement was conducted in triplicate to ensure reliability.

### Optimization of selenoprotein extraction process

#### Single-factor experiment design

All extractions were conducted using freeze-dried sample powder as the starting material, and extraction yields were calculated on a dry weight basis. Precisely 0.1 g of *Cynanchum thesioides* powder was weighed into a 10 mL centrifuge tube, and the effects of extraction time (1.0, 1.5, 2.0, 2.5, 3.0 h), extraction temperature (30, 35, 40, 45, 50 °C), solid-to-liquid ratio (1:20, 1:25, 1:30, 1:35, 1:40 g/mL), NaOH concentration (0.05, 0.10, 0.15, 0.20, 0.25 mol/L), HCl concentration (0.05, 0.10, 0.15, 0.20, 0.25 mol/L), NaCl concentration (0.05, 0.10, 0.15, 0.20, 0.25 mol/L), and ethanol volume fraction (60%, 65%, 70%, 75%, 80%) on the extraction yield of selenoprotein were systematically investigated. The final optimized conditions for each extraction method—including the solid-to-liquid ratio, mixing/vortexing conditions, pH after mixing, temperature, and time—are provided in Table 1. The optimal ranges identified through these single-factor experiments serve as preliminary references, with final optimization to be based on the results of the RSM.

#### Response Surface Methodology for process optimization

Following the single-factor experiments, a three-factor, three-level Box-Behnken design (BBD) was developed using Design-Expert software, version 13.0. The response variable was set as the selenoprotein extraction yield. Each set of experiments was conducted independently three times. The factors and their respective levels for each extraction method were defined as follows:

Water extraction: extraction temperature (A: 25, 30, 35 °C), extraction time (B: 1.5, 2.0, 2.5 h), solid-to-liquid ratio (C: 1:15, 1:20, 1:25 g/mL).

Alkaline extraction: solid-to-liquid ratio (A: 1:20, 1:25, 1:30), extraction temperature (B: 35, 40, 45 °C), NaOH concentration (C: 0.15, 0.20, 0.25 mol/L).

Acid extraction: solid-to-liquid ratio (A: 1:25, 1:30, 1:35), extraction temperature (B: 45, 50, 55 °C), HCl concentration (C: 0.15, 0.20, 0.25 mol/L).

Salt extraction: solid-to-liquid ratio (A: 1:20, 1:25, 1:30), extraction temperature (B: 30, 35, 40 °C), NaCl concentration (C: 0.10, 0.15, 0.20 mol/L).

Organic solvent extraction: solid-to-liquid ratio (A: 1:20, 1:25, 1:30), extraction temperature (B: 45, 50, 55 °C), ethanol volume fraction (C: 75%, 80%, 85%).

An ANOVA was employed to determine the significance of the model and its terms, facilitating the derivation of optimal extraction parameters. A Box-Behnken design (BBD) with five center point replicates and fully randomized run order was applied for each extraction method. A second-order polynomial model was fitted, and its adequacy was confirmed by lack-of-fit tests (*p* > 0.05 for all models). The coded factor levels and actual values are provided in Table S1. The final regression equations and coefficients (±SE) for selenoprotein yield (Y) under aqueous, alkaline, acid, salt, and alcohol extraction methods are summarized in Table S2.

**Table 1:** Optimization results and verification of five extraction methods.

Extraction method	Optimal conditions	Theoretical yield (mg/g)	Actual yield (mg/g)	Relative error (%)
Water extraction	*t* = 2.29 h; R = 1:15.87; *T* = 28.64 °C	2.741	2.738	0.11
Alkaline extraction	R = 1:22.7; *T* = 44.62 °C; C_NaOH_ = 0.243 mol/L	5.925	5.924	0.02
Acid extraction	R = 1:28.6; *T* = 51.60 °C; C_HCl_ = 0.192 mol/L	2.762	2.761	0.04
Salt extraction	R = 1:21.74; *T* = 35.99 °C; C_NaCl_ = 0.141 mol/L	4.731	4.730	0.02
Organic solvent extraction	R = 1:23.3; *T* = 52.49 °C; C_EtOH_ = 81.37%	3.271	3.268	0.09

**Notes.**

t extraction time R solid-to-liquid ratio (g/mL) T temperature C_NaOH_ NaOH concentration C_HCl_ HCl concentration C_NaCl_ NaCl concentration C_EtOH_ ethanol concentration

### Effects of selenium fertilizer on selenium content and *in vitro* antioxidant activity of *C. thesioides* fruits

The selenium source was selenomethionine (Product No. S3875; Sigma Aldrich, St. Louis, MO, USA) purity > 99%). Foliar spraying was performed using a handheld sprayer equipped with a flat-fan nozzle to uniformly wet both sides of the leaves until the runoff point (*i.e.,* the critical dripping point) was nearly reached. The spraying volume was 50 mL per plant. All spraying operations were conducted in windless or breezy early mornings to minimize spray drift. The experiment followed a randomized complete block design (RCBD) with three blocks (serving as replicates). Within each block, all treatments were randomly assigned, and each treatment plot contained 10 plants. Samples were collected 15 days after the final spraying to assess the stable uptake and transformation of selenium. The experiment included four selenomethionine concentration treatments: 0 (non-selenium-enriched matrix control), 2, 4, and 6 mg/L (pH∼6.5). Applications were administered at seven-day intervals, resulting in a total of four treatments. Fruit samples were harvested 15 days after the final application. For analysis, 0.5 g of freeze-dried fruit powder was digested, and the total selenium content was quantified using graphite furnace atomic absorption spectrometry (GFAAS). An additional sample was subjected to an optimal alkaline extraction process (0.243 mol/L NaOH, solid-to-liquid ratio of 1:22.7, 44.62 °C) to obtain the alkaline-soluble fraction. This fraction was then centrifuged at 4,000 × g for 15 min at 4 °C. The supernatant was digested using a microwave digestion system (with 2.5 mL HNO_3_ and 0.5 mL H_2_O_2_), followed by acid removal and volume adjustment, and subsequently stored at 4 °C for the subsequent determination of alkaline-extractable selenium content. The atomic absorption measurement conditions were as follows: wavelength 196.0 nm, lamp current 7.0 mA, slit width 1.3 nm, time constant 0.1 s, and photomultiplier tube (PMT) negative high voltage of 370 V, using 1% (w/v) Ni(NO_3_)_2_ as a matrix modifier.

The resulting alkaline-extracted protein solution was assessed for its *in vitro* antioxidant capacity. The 2,2-diphenyl-1-picrylhydrazyl (DPPH) radical scavenging activity was determined as follows: the reaction was conducted at 25 °C in the dark for 30 min, and the absorbance was measured at 517 nm using a one cm pathlength cuvette (approximately pH 6.5). The DPPH reagent (CAS: 1898-66-4) and all solvents were purchased from Thermo Fisher Scientific. The concentration of the stock solution was calibrated using a molar absorptivity of *ɛ* = 1.02 ×10^4^ L mol^−^^1^ cm^−^^1^ as established in the literature (Maleš et al., 2023). The ferric reducing antioxidant power (FRAP) assay was performed using a freshly prepared aqueous solution of ferrous sulfate (FeSO_4_) to generate the calibration curve, and results were expressed as absorbance at 593 nm, following the protocol described by Assimakopoulou (2006). Hydroxyl radical scavenging activity was quantified using the salicylic acid method (Yang et al., 2003), and the superoxide anion radical scavenging activity was determined *via* the pyrogallol autoxidation method (Guo et al., 2023). All extracts were normalized to a protein concentration of 5 mg/mL prior to antioxidant assays. All assays were conducted in triplicate. Data are presented as the mean ± standard deviation. All statistical analyses were performed using IBM SPSS Statistics (v 22.0; IBM Corp, Armonk, NY, USA) and Design-Expert (v 13.0). Normality (Shapiro–Wilk test) and homogeneity of variances (Levene’s test) were verified prior to parametric tests. Data meeting assumptions were analyzed with ANOVA followed by Tukey’s HSD *post-hoc* test; otherwise, nonparametric alternatives (*e.g.*, Kruskal–Wallis H test) were applied. The significance level was *α* = 0.05, and effect sizes (*η*^2^) are reported alongside *p*-values.

## Results

### Analysis of nutritional components in *C. thesioides* fruits

Both *C. thesioides* and *C. thesioides* var. *australe* fruits displayed a marked reduction in moisture content correlating with an increase in fruit size (*P* < 0.05, Table 2). Specifically, the moisture content in large fruits decreased by 18.39% and 19.44% in *C. thesioides* and *C. thesioides* var. *australe*, respectively. Although the moisture content of *C. thesioides* var. *australe* fruits was generally lower than that of *C. thesioides* across all size classes, the interspecific differences were not statistically significant (*P* > 0.05).

Vitamin C content changed significantly during fruit maturation (*P* < 0.05), showing a decrease then increase in both species. The highest vitamin C concentrations were in small fruits (*C. thesioides*: 107.09 mg/100 g; *C. thesioides* var. australe: 135.10 mg/100 g), while the lowest were in medium-sized fruits (95.25 mg/100 g and 111.03 mg/100 g, respectively). Throughout all size classes, the vitamin C content in *C. thesioides* fruits was consistently lower than that in *C. thesioides* var. *australe* (*P* < 0.05, *η*^2^=0.985, Table S5).

The content of soluble sugars decreased progressively with an increase in fruit size, revealing significant differences among the size classes within each species (*P* < 0.05). The highest percentages were found in the smaller fruits (*C. thesioides*: 3.52%; *C. thesioides* var. *australe*: 3.55%), while the lowest were observed in the larger fruits (*C. thesioides*: 3.42%; *C. thesioides* var. *australe*: 3.45%). Both the large and small fruits of *C. thesioides* possessed significantly lower soluble sugar content compared to those of *C. thesioides* var. *australe* (*P* < 0.05, *η*^2^=0.978, Table S5).

**Table 2:** Analysis of nutritional components in fruits of *C. thesioides* and *C. thesioides* var. *australe*.

Indicator	CS-SF	CS-MF	CS-LF	CA-SF	CA-MF	CA-LF
Moisture content (%)	90.01 ± 2.12 Aa	80.75 ± 3.62 Ab	71.62 ± 0.71 Ac	86.88 ± 2.80 Aa	71.97 ± 0.44 Ab	67.44 ± 1.41 Ac
Vitamin C (mg/100 g)	107.09 ± 1.81 Ba	95.25 ± 1.81 Bc	101.563 ± 2.36 Bb	135.10 ± 1.81 Aa	111.03 ± 2.36 Ac	122.08 ± 1.81Ab
Soluble sugar (%)	3.52 ± 0.005 Ba	3.48 ± 0.003 Ab	3.42 ± 0.004 Bc	3.55 ± 0.004 Aa	3.48 ± 0.004Ab	3.45 ± 0.005Ac
Crude fiber (%)	1.83 ± 0.003 Ab	1.90 ± 0.002 Aa	1.90 ± 0.007 Ba	1.87 ± 0.005 Ab	1.89 ± 0.003Ab	1.95 ± 0.015Aa
Crude fat (%)	2.60 ± 0.002 Ac	5.60 ± 0.05 Ab	5.40 ± 0.014 Aa	2.0 ± 0.139 Bc	3.30 ± 0.017Bb	7.30 ± 0.386Ba
Soluble protein (mg/g)	5.79 ± 0.071 Ba	4.33 ± 0.179 Bc	4.61 ± 0.035 Bb	8.04 ± 0.036 Aa	5.90 ± 0.035 Ab	5.61 ± 0.107Ac
Selenium (mg/kg)	0.66 ± 0.014 Bc	0.724 ± 0.018Ab	1.01 ± 0.017 Ba	0.69 ± 0.012 Ac	0.77 ± 0.043Ab	1.06 ± 0.022Aa

**Notes.**

CS *Cynanchum thesioides* CA *C. thesioides* var. *australe* SF Small fruit MF Medium fruit LF Large fruit

Different uppercase letters indicate significant differences between species within the same fruit size (*P* < 0.05), and different lowercase letters indicate significant differences among fruit sizes within the same species (*P* < 0.05).

In contrast, crude fiber content increased significantly with fruit enlargement (*P* < 0.05). Maximum values were recorded in the large fruits (*C. thesioides*: 1.903%; *C. thesioides* var. *australe*: 1.948%), with the minimum values in small fruits (*C. thesioides*: 1.834%; *C. thesioides* var. *australe*: 1.869%). The crude fiber content in the large and small fruits of *C. thesioides* var. *australe* was higher than that in *C. thesioides*, although the medium-sized fruits of *C. thesioides* var. australe exhibited a significantly lower content than those of *C. thesioides* (*P* < 0.05, *η*^2^=0.88, Table S5).

*C. thesioides* demonstrated an initial increase followed by a decrease, with the peak fat content occurring in medium-sized fruits (5.6%). In contrast, *C. thesioides* var. *australe* showed a consistent increase in fat content with fruit size, reaching the highest level in large fruits (7.3%). The lowest fat content was noted in the small fruits of both species (*C. thesioides*: 2.6%; *C. thesioides* var. *australe*: 2.0%), with significant differences across the size classes (*P* < 0.05). In terms of interspecific differences, the crude fat content in the medium and small fruits of *C. thesioides* was significantly higher than that in *C. thesioides* var. *australe*; however, the large fruits of *C. thesioides* var. *australe* had significantly higher fat content compared to those of *C. thesioides* (*η*^2^=0.999, Table S5).

The content of soluble protein in *C. thesioides* initially decreased before increasing, whereas *C. thesioides* var. australe exhibited a consistent decrease as the fruit size increased. Highest concentrations were in small fruits (*C. thesioides*: 5.74 mg/g; *C. thesioides* var. australe: 8.04 mg/g), and lowest in *C. thesioides* medium fruits (4.29 mg/g) and *C. thesioides* var. australe large fruits (5.61 mg/g). Across all size classes, the soluble protein content of *C. thesioides* was significantly lower than that of *C. thesioides* var. *australe* (*P* < 0.05, *η*^2^=0.996, Table S5).

The selenium content increased significantly with the size of the fruit in both species (*P* < 0.05). The highest selenium concentrations were found in the large fruits (*C. thesioides*: 1.01 mg/kg; *C. thesioides* var. *australe*: 1.06 mg/kg), while the small fruits exhibited the lowest concentrations (*C. thesioides*: 0.66 mg/kg; *C. thesioides* var. *australe*: 0.69 mg/kg). Throughout all size classes, the selenium content in the fruits of *C. thesioides* was significantly lower than that in the fruits of *C. thesioides* var. *australe* (*P* < 0.05, *η*^2^ = 0.985).

### Optimization of selenoprotein extraction process

Based on the outcomes of single-factor experiments illustrated in Fig. S2, RSM was utilized to methodically optimize the extraction processes involving water, alkaline, acid, salt, and organic solvents. This optimization pertains to the extraction of soluble Se-containing proteins from the fruits of *C. thesioides*, with the analytical details presented in Tables S3 and S4, which include ANOVA for RSM analysis and the regression models respectively. The parameters of the models and their validation outcomes for each extraction method are concisely presented in Table 1. For the water extraction process, the optimal parameters were established as an extraction time of 2.29 h, a solid-to-liquid ratio of 1:15.87 (g/mL), and a temperature of 28.64 °C (*P* < 0.0001, *R*^2^ = 0.993). These conditions predicted a theoretical yield of 2.741 mg/g. Subsequent validation experiments yielded an actual extraction of 2.738 mg/g, with a relative error under 0.11%, thus confirming the high predictive accuracy of this model. In the case of alkaline extraction, the most effective yield was 5.925 mg/g, obtained under conditions of a solid-to-liquid ratio of 1:22.7, a temperature of 44.62 °C, and a NaOH concentration of 0.243 mol/L (*P* < 0.05, *R*^2^ = 0.906). Validation of these results underscored the model’s reliability. Optimal conditions for acid extraction included a solid-to-liquid ratio of 1:28.6, a temperature of 51.60 °C, and an HCl concentration of 0.192 mol/L, leading to a theoretical yield of 2.762 mg/g (*P* < 0.05, *R*^2^ = 0.907). Salt extraction demonstrated its maximum yield of 4.731 mg/g at a solid-to-liquid ratio of 1:21.74, a temperature of 35.99 °C, and an NaCl concentration of 0.141 mol/L (*P* < 0.05, *R*^2^ = 0.841). For organic solvent extraction, the conditions optimized were a solid-to-liquid ratio of 1:23.3, a temperature of 52.49 °C, and an ethanol volume fraction of 81.37%, which resulted in a theoretical yield of 3.271 mg/g (*P* < 0.05, *R*^2^ = 0.950). Comparative analysis of these methods indicates that alkaline extraction was the most efficient, followed in descending order by salt, acid, organic solvent, and water extractions.

### Interactive effects of process parameters on the yield of selenium-containing proteins from *cynanchum thesioides* fruit using different extraction methods

**Figure 1:**
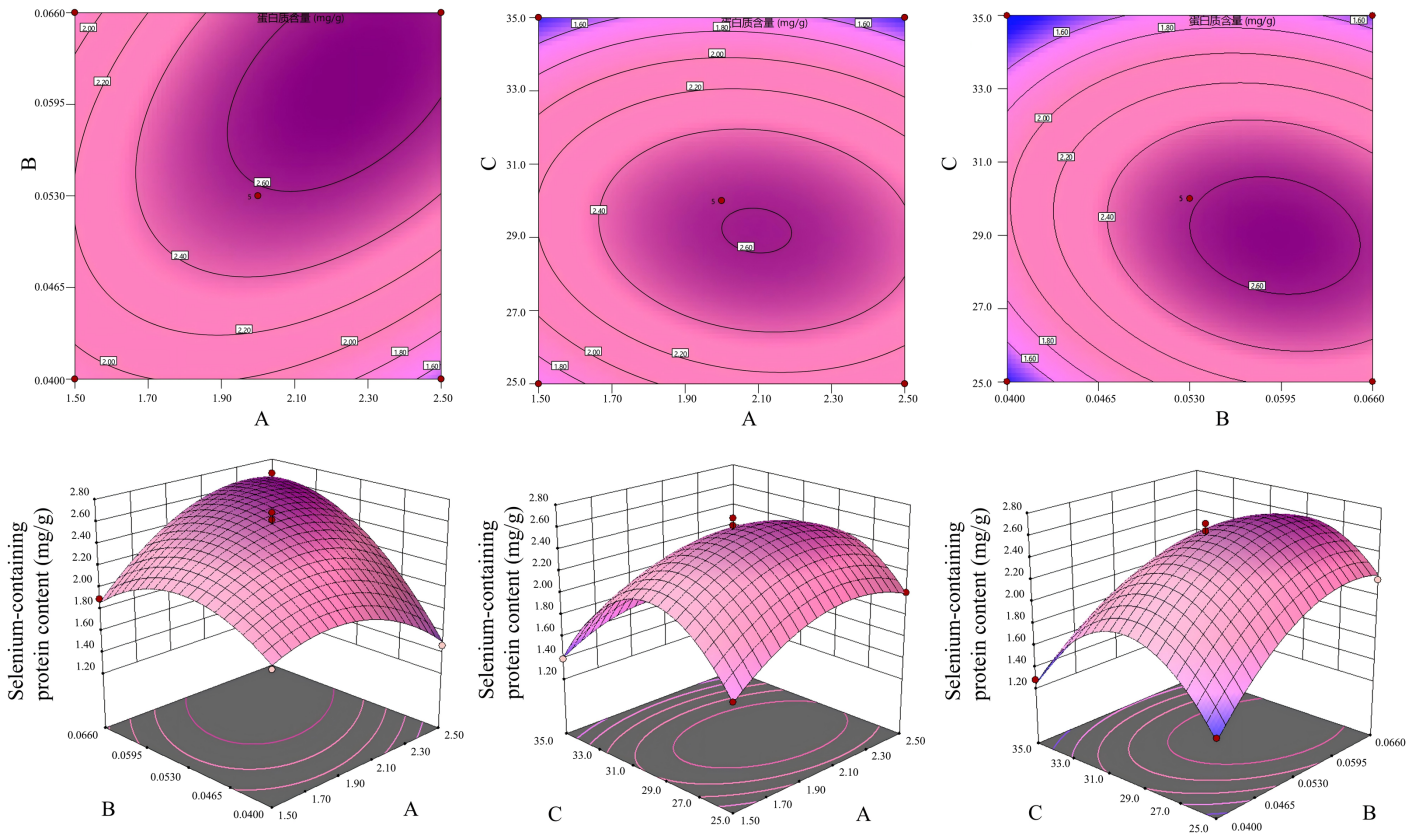
Effects of various two-factor interactions on the soluble selenium-containing protein content in the fruit of *Cynanchum thesioides*. (A) Contour plot; (B) Three-dimensional response surface plot. In the figure, letters A, B, and C denote temperature, time, and solid-to-liquid ratio, respectively. The steepness of the response surface and the ellipticity of the contour reflect the significance of the interaction between the corresponding two factors. Steeper surfaces and more elliptical contours indicate more significant interactive effects.

Figure 1 and Figure S3 present response surface and contour plots illustrating the interactive effects of various factors on the protein yield during the extraction of selenium-containing proteins from the fruit of *Cynanchum thesioides* (Freyn) K. Schum. using water, alkaline, acid, salt, and organic solvent-based methods. Specifically, for the water extraction method, both the AB and BC interactions were significant, with BC exhibiting the greatest influence. In the alkaline extraction method, the AB interaction was the most pronounced. For the acid extraction method, the AB and AC interactions were significant, and factors A, B, and C all showed notable individual effects. In the salt extraction method, significant interactions were observed for AB and BC, with factors A and C exerting particularly strong effects. For the organic solvent extraction method, the AB and BC interactions were significant, and factors A and B demonstrated relatively prominent influences. The data points in the residual plot of the response surface show random scatter without obvious trends or patterns, indicating that the model is adequately fitted and the prediction errors fall within an acceptable range of random variation (Fig. S4). Overall, analysis reveals clear differences in the combinations of factors involved in significant interactions and their respective degrees of influence across the different extraction methods.

### Effects of selenium fertilizer concentration on selenium content in fruits and extracts, and *in vitro* antioxidant activity

**Figure 2:**
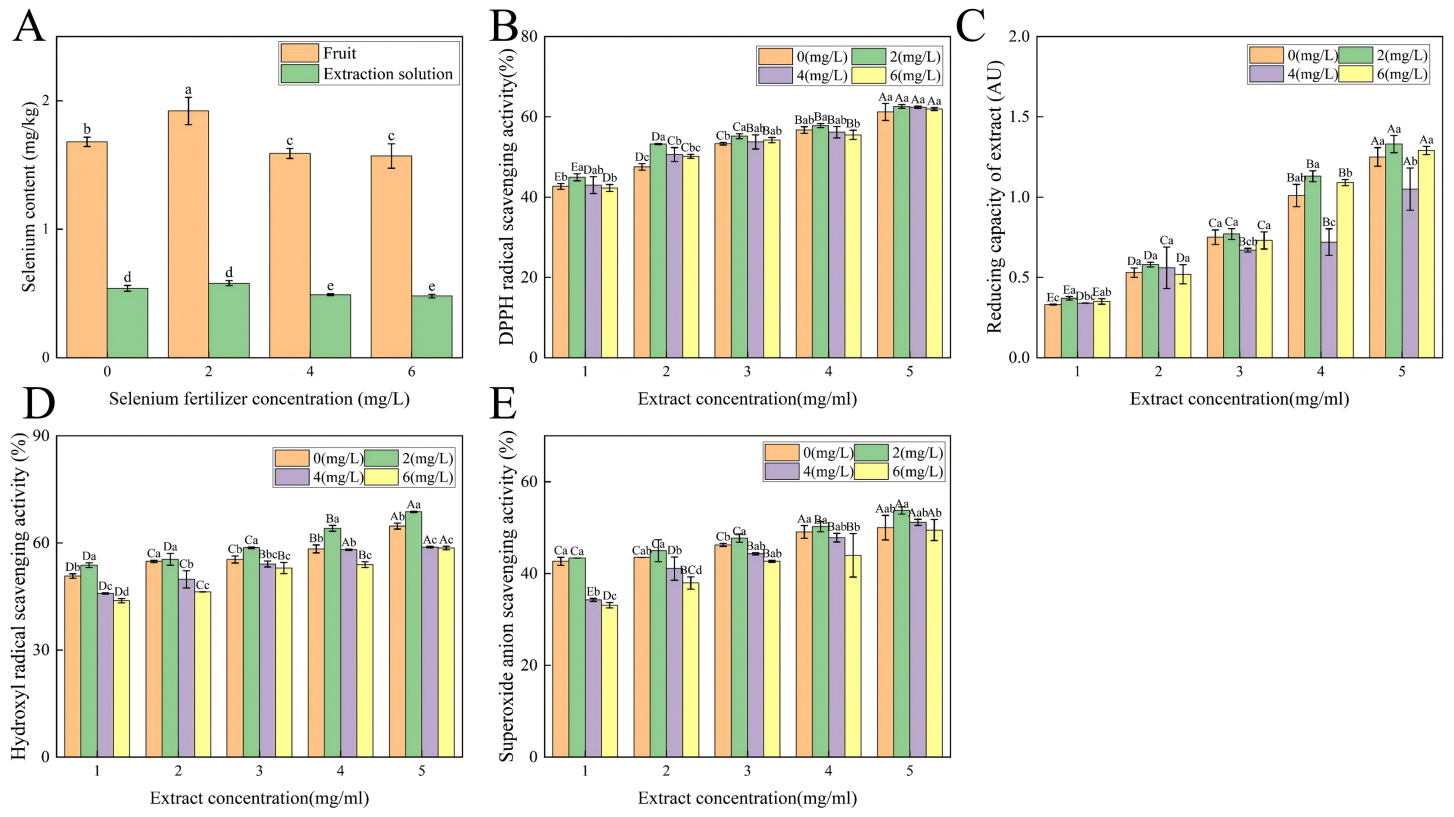
Effects of selenium fertilizer treatment on selenium content and antioxidant activity in *C. thesioides* fruits. (A) Selenium content variations in fruits and their alkaline-extracted protein fractions in response to different selenium fertilizer concentrations. The horizontal axis represents the concentration of selenium fertilizer (mg/L), and the vertical axis represents the selenium content (mg/kg) in *Cynanchum thesioides* fruits and their alkaline extracts; (B) DPPH radical scavenging activity; (C) Ferric reducing antioxidant power (FRAP); (D) Hydroxyl radical scavenging activity; (E) Superoxide anion scavenging activity. The horizontal axis represents the extract concentration (mg/mL), and the vertical axis represents both the scavenging rate (%) and the reducing power (AU). Different letters indicate significant differences among treatments at *P* < 0.05.

The foliar application of selenium markedly influenced the accumulation of selenium in the fruits of *C. thesioides* (Fig. 2A). Observations revealed that both the total selenium content in the fruits and the selenium concentration in the alkaline-extracted protein fraction initially increased and then diminished as the concentration of selenium fertilizer escalated, reaching a zenith at the 2 mg/L treatment (total Se in fruits: 1.92 mg/kg; Se in extract: 0.58 mg/kg). The selenium levels in this experimental set were significantly elevated compared to those in other groups (*P* < 0.05). Notably, the selenium content recorded for the 6 mg/L treatment was even lower than that observed in the control group devoid of selenium supplementation. Furthermore, the alkaline-extracted protein fraction demonstrated a concentration-dependent antioxidant activity *in vitro* (Figs. 2B–2E). At a protein concentration of 5 mg/mL, extracts derived from the 2 mg/L selenium treatment displayed the most potent scavenging capacities against all evaluated free radicals (DPPH⋅, ⋅ OH, and O_2_⋅^−^) and exhibited the highest ferric ion reducing antioxidant power (FRAP value), with activities significantly surpassing those of the control group (*P* < 0.05). Precisely, the scavenging rate for the DPPH radical peaked at 62.58% (*η*^2^ = 0.985, *R*^2^ = 0.9991, with IC_5__0_ values ranging from 0.98 mg/L (in the 2 mg/L Se treatment group) to 4.21 mg/L), the hydroxyl radical scavenging rate was 68.73% (*η*^2^ = 0.911, *R*^2^ = 0.9759, with IC_5__0_ values ranging from 1.18 mg/L (in the 2 mg/L Se treatment group) to 5.13 mg/L), the superoxide anion scavenging rate was 53.74% (*η*^2^ = 0.949, *R*^2^ = 0.9612, with IC_5__0_ values ranging from 1.98 mg/L (in the 2 mg/L Se treatment group) to 6.98 mg/L) (Table S5), and the FRAP value reached 1.33 AU. Analysis revealed a significant positive correlation between the selenoprotein extracts and their antioxidant activity (Fig. S5, Table S6).

## Discussion

### Nutritional value and selenium-enriching characteristics of *C. thesioides*

In comparison to commonly consumed vegetables such as eggplant, potato, and spinach, *C. thesioides* and its variant exhibited significant enhancements in the content of vitamin C, crude fat, and crude fiber. Their soluble protein content was found to be on par with that of conventional vegetables, preliminarily validating their potential as superior wild vegetable resources (Akyol et al., 2016; Puccinelli, Malorgio & Pezzarossa, 2017; Wang et al., 2022). Notably, the selenium content in the fruits of both types was measured at up to 1.06 mg/kg, a concentration markedly higher than that found in most conventional vegetables (Gupta & Gupta, 2017). Additionally, selenium levels were observed to increase during the fruit development stages, underscoring the exceptional characteristic of *C. thesioides* as a naturally selenium-enriched plant. Smaller fruits predominantly accumulated moisture, vitamin C, soluble sugars, and proteins—components closely linked with quality—whereas larger fruits tended to amass greater amounts of crude fiber, crude fat, and selenium, which are primarily associated with storage or structural functions. This reflects its resource allocation strategy during growth and development (Grassein, Till-Bottraud & Lavorel, 2010). Thus, *C. thesioides* not only serves as a nutrient-dense food source but also represents an optimal subject for selenium biofortification research due to its robust capability for selenium enrichment.

### Optimization and selection of extraction processes

The study revealed that the alkaline extraction method, utilizing NaOH, exhibited the highest yield of 5.925 mg/g under optimal conditions. This superior yield is primarily attributed to the efficacy of NaOH in disrupting cellular wall structures, cleaving hydrogen bonds, and increasing the net negative charge on the surfaces of protein molecules, which significantly enhances their solubility (Hewage et al., 2022). Despite its effectiveness, the strongly alkaline conditions may induce peptide bond hydrolysis, oxidize selenoamino acids, and lead to the formation of undesirable byproducts such as lysinoalanine (Alzuwaid et al., 2021). The yield benefit of alkaline extraction *via* cell-wall disruption must be weighed against the risks of lysinoalanine formation and selenium oxidation, making downstream desalting and neutralization essential for food-grade safety (Park et al., 2025; Wu et al., 2025; Xu et al., 2025; Kurniawan, Criselda & Lilian, 2021). Salt extraction, yielding 4.731 mg/g, effectively utilizes the ionic strength effect to neutralize surface charges on proteins, diminish electrostatic repulsion between molecules. This method is particularly advantageous for extracting globulins that are sensitive to acids and alkalis (Zakki, Aryanti & Hadiyanto, 2024). Conversely, acid extraction produced less favorable outcomes, which likely resulted from the extraction pH approaching the isoelectric point of the target proteins, thereby minimizing solubility and inducing precipitation (Budiawan Bakri et al., 2018). Organic solvent extraction, primarily targeting alcohol-soluble proteins, appeared inadequate for *C. thesioides* Se-containing proteins, which do not fit this solubility profile (Ahmed, Hammad & Mawgood, 2025). By systematically comparing five crude extraction methods, this study establishes key preprocessing methodological guidance for subsequent selenoprotein research in *C. thesioides*. The significant differences in extraction efficiency observed suggest that the unique matrix of this plant (*e.g.*, its secondary metabolites) may profoundly influence selenoprotein extraction. This provides a new perspective for understanding selenoprotein studies in non-model plants and lays the primary methodological foundation for developing selenium-enriched functional products from this distinctive resource. Nevertheless, this inference necessitates further validation through comparative analyses of the functional properties, including structural integrity and specific activity, of the protein products obtained *via* different methods. A key limitation of this study lies in its primary focus on extraction yield and selenium content, without systematic evaluation of protein structural integrity, functional properties, or alkali-induced safety risks across extraction methods. The absence of these data constrains process optimization for food applications. Future work should therefore prioritize comprehensive characterization of protein structure (*e.g.*, SDS-PAGE, FTIR), functionality, digestibility, and performance in food-simulating systems, to establish a multidimensional evaluation framework that integrates nutritional, functional and safety aspects beyond yield alone.

### Effects of exogenous selenium fortification on functional activity

Foliar selenium application markedly influenced the accumulation of selenium in the fruits of *C. thesioides*, exhibiting a characteristic dose–response curve. Initially, selenium content increased and subsequently declined with escalating concentrations of exogenous selenium. The peak of selenium content was observed in the treatment group receiving 2 mg/L (1.92 mg/kg), delineating this concentration as a pivotal threshold for optimal selenium assimilation and tolerance in *C. thesioides*. Concentrations exceeding this threshold appeared to provoke phytotoxic responses that hindered further selenium uptake or accumulation (Gupta & Gupta, 2017; Zhou et al., 2020). Selenium exhibits a dual dose–response in plants: low concentrations (*e.g.*, 2 mg/L) enhance stress resistance *via* antioxidant enzyme incorporation, while high levels induce toxicity through sulfur pathway antagonism, explaining yield and quality decline (Hasanuzzaman et al., 2020). Our applied concentration of 2 mg/L falls within the recommended foliar range (1–10 mg/L), representing an economically efficient and sustainable practice for selenium biofortification. Specifically, the alkaline-extracted protein fraction from the 2 mg/L treatment group demonstrated the most potent activity across various *in vitro* antioxidant assays, including DPPH radical, hydroxyl radical, and superoxide anion scavenging capacities, as well as FRAP. The activity showed concentration dependence, exceeding BHT yet lower than vitamin C (Talbi et al., 2019; Kurek et al., 2022). Firstly, selenium, an integral component of antioxidant enzymes such as GPx, directly engages in the scavenging of free radicals (Kong et al., 2019); secondly, the incorporation of selenium might modify protein conformation, thereby exposing active sites (Chauhan et al., 2020) and enhancing interaction with coexisting antioxidant constituents, such as polyphenols, in the extract. The observed decrease in antioxidant activity at selenium concentrations exceeding 4 mg/L can be linked to the oxidative stress induced by selenium excess, which potentially leads to protein structural damage or the accumulation of deleterious substances (Wang et al., 2016).

### Implications of optimization outcomes

Single-factor experiments efficiently narrow the investigation range of key variables, laying the foundation for response surface design, while response surface analysis surpasses the limitations of the single-factor approach by successfully revealing significant synergistic effects among critical factors such as alkali concentration and extraction temperature. The resulting optimal process parameters represent a globally optimal solution with interactive compensatory effects, thereby exhibiting enhanced robustness. These optimization outcomes not only explain the high selenoprotein yield achieved through the alkaline extraction method but also provide a process-based rationale for the superior antioxidant activity of the extracts. This study has three main limitations: the lack of selenium speciation analysis (*e.g.*, the Sec/SeMet ratio), the absence of *in vivo* validation (*e.g.*, using cellular or animal models to confirm bioactivity), and the lack of cross-validation for the response surface models, as further experimental validation was not feasible. Consequently, the interpretation of the model predictions should be focused on directional guidance and factor screening. Addressing these limitations would enhance the scientific rigor of the research and provide clear pathways for future studies.

## Conclusions

Employing RSM, the research optimized five selenoprotein extraction processes, determining the optimal parameters for each method. The alkaline extraction method proved optimal, yielding the highest selenoprotein content (5.93 mg/g DW) under conditions of a 1:22.7 solid-to-liquid ratio, 0.243 mol/L NaOH, at 44.62 °C for 2 h. This yield was significantly greater than those achieved by aqueous, acidic, saline, or ethanolic extraction. Foliar application of selenium at 2 mg/L optimally enhanced fruit Se content to 1.92 mg/kg DW. The extracted selenoprotein from the 2 mg/L Se treatment group exhibited potent *in vitro* antioxidant activity, with an IC_5__0_ against DPPH radicals of 0.98 mg/L (95% CI [0.85–1.15] mg/L). Therefore, combining alkaline extraction with 2 mg/L foliar Se fertilization represents an effective strategy for co-producing Se-enriched functional ingredients with high yield and strong antioxidant capacity.

##  Supplemental Information

10.7717/peerj.20998/supp-1 Supplemental Information 1 Schematic representation of the experimental design and analytical procedure

10.7717/peerj.20998/supp-2 Supplemental Information 2 Effects of key extraction parameters on selenoprotein yield in the aqueous extraction process The horizontal axis represents the four factors, namely time, temperature, solid-to-liquid ratio, and solvent concentration, respectively. The vertical axis shows the selenium content (mg/g). Different lowercase letters above the bars indicate significant differences among the treatments at the 0.05 level.

10.7717/peerj.20998/supp-3 Supplemental Information 3 Effects of key extraction parameters on selenoprotein yield in the alkaline extraction process The horizontal axis represents the four factors, namely time, temperature, solid-to-liquid ratio, and solvent concentration, respectively. The vertical axis shows the selenium content (mg/g). Different lowercase letters above the bars indicate significant differences among the treatments at the 0.05 level.

10.7717/peerj.20998/supp-4 Supplemental Information 4 Effects of key extraction parameters on selenoprotein yield in the acid extraction process The horizontal axis represents the four factors, namely time, temperature, solid-to-liquid ratio, and solvent concentration, respectively. The vertical axis shows the selenium content (mg/g). Different lowercase letters above the bars indicate significant differences among the treatments at the 0.05 level.

10.7717/peerj.20998/supp-5 Supplemental Information 5 Effects of key extraction parameters on selenoprotein yield in the salt extraction process The horizontal axis represents the four factors, namely time, temperature, solid-to-liquid ratio, and solvent concentration, respectively. The vertical axis shows the selenium content (mg/g). Different lowercase letters above the bars indicate significant differences among the treatments at the 0.05 level.

10.7717/peerj.20998/supp-6 Supplemental Information 6 Effects of key extraction parameters on selenoprotein yield in the organic solvent extraction process The horizontal axis represents the four factors, namely time, temperature, solid-to-liquid ratio, and solvent concentration, respectively. The vertical axis shows the selenium content (mg/g). Different lowercase letters above the bars indicate significant differences among the treatments at the 0.05 level.

10.7717/peerj.20998/supp-7 Supplemental Information 7 Effects of two-factor interactions on the content of soluble selenium-containing proteins extracted by alkaline extraction from the fruit of *C. thesioides* A, B, and C represent the solid-to-liquid ratio, temperature, and solvent concentration, respectively.

10.7717/peerj.20998/supp-8 Supplemental Information 8 Effects of two-factor interactions on the content of soluble selenium-containing proteins extracted by acid extraction from the fruit of *C. thesioides* A, B, and C represent the solid-to-liquid ratio, temperature, and solvent concentration, respectively.

10.7717/peerj.20998/supp-9 Supplemental Information 9 Effects of two-factor interactions on the content of soluble selenium-containing proteins extracted by salt extraction from the fruit of* C. thesioides* A, B, and C represent the solid-to-liquid ratio, temperature, and solvent concentration, respectively.

10.7717/peerj.20998/supp-10 Supplemental Information 10 Residual plot of the aqueous extraction optimization model The four images are: Normal Probability Plot of Residuals, Plot of Residuals vs Predicted Values, Plot of Predicted Values vs Actual Values, and Cook’s Distance Plot.

10.7717/peerj.20998/supp-11 Supplemental Information 11 Effects of two-factor interactions on the content of soluble selenium-containing proteins extracted using organic solvent extraction from the fruit of *C. thesioides* A, B, and C represent the solid-to-liquid ratio, temperature, and solvent concentration, respectively.

10.7717/peerj.20998/supp-12 Supplemental Information 12 Residual plot for the alkaline extraction optimization model The four images are: Normal Probability Plot of Residuals, Plot of Residuals vs Predicted Values, Plot of Predicted Values vs Actual Values, and Cook’s Distance Plot.

10.7717/peerj.20998/supp-13 Supplemental Information 13 Residual plot for the acid extraction optimization model The four images are: Normal Probability Plot of Residuals, Plot of Residuals vs Predicted Values, Plot of Predicted Values vs Actual Values, and Cook’s Distance Plot.

10.7717/peerj.20998/supp-14 Supplemental Information 14 Residual plot for the salt extraction optimization model The four images are: Normal Probability Plot of Residuals, Plot of Residuals vs Predicted Values, Plot of Predicted Values vs Actual Values, and Cook’s Distance Plot.

10.7717/peerj.20998/supp-15 Supplemental Information 15 Residual plot for the ethanol extraction optimization model The four images are: Normal Probability Plot of Residuals, Plot of Residuals vs Predicted Values, Plot of Predicted Values vs Actual Values, and Cook’s Distance Plot.

10.7717/peerj.20998/supp-16 Supplemental Information 16 Free radical scavenging activity under different treatments The x-axis represents the treatment method, and the y-axis represents the clearance rate (%).1-5: The selenium fertilizer concentration is 0 mg/L, and the extract concentrations are 1, 2, 3, 4, and 5 mg/mL respectively; 6-10: The selenium fertilizer concentration is 2 mg/L, and the extract concentrations are 1, 2, 3, 4, and 5 mg/mL respectively; 11-15: The selenium fertilizer concentration is 4 mg/L, and the extract concentrations are 1, 2, 3, 4, and 5 mg/mL respectively; 16-20: The selenium fertilizer concentration is 0 mg/L, and the extract concentrations are 1, 2, 3, 4, and 5 mg/mL respectively.

10.7717/peerj.20998/supp-17 Supplemental Information 17 Coefficients of coded factors for different extraction methods

10.7717/peerj.20998/supp-18 Supplemental Information 18 Analysis of variance for regression models of different extraction methods

10.7717/peerj.20998/supp-19 Supplemental Information 19 Optimization results of response surface methodology for soluble selenium-containing proteins from Cynanchum thesioides fruits using different extraction methods

10.7717/peerj.20998/supp-20 Supplemental Information 20 Summary statistics of the model

10.7717/peerj.20998/supp-21 Supplemental Information 21 Confidence intervals and goodness-of-fit for the determination of various indicators

10.7717/peerj.20998/supp-22 Supplemental Information 22 Correlation analysis between selenium fertilizer concentration and antioxidant activity indicators Note: *The correlation is significant at the 0.05 level (two-tailed). ** The correlation is significant at the 0.01 level (two-tailed).

10.7717/peerj.20998/supp-23 Supplemental Information 23 Optimization of extraction process of selenoprotein and analysis of antioxidant capacity in vitro of Cynanchun thesioides (Freyn).K.Schum Han (2023). Optimization of selenium protein extraction process from Cynanchum thesioides and analysis of its in vitro antioxidant activity (Doctoral Thesis). Inner Mongolia Agricultural University, Hohhot.

## Additional Information and Declarations

### Data Availability

The following information was supplied regarding data availability: The data is available at Zenodo: Men, Y. (2025). Optimization of Extraction Process and Analysis of *In Vitro* Antioxidant Capacity of Selenoproteins from *Cynanchum thesioides* [Data set]. Zenodo. https://doi.org/10.5281/zenodo.17920873.
