# Supplementary figures and images for: Optimization of the extraction process and in vitro antioxidant capacity analysis of selenium-containing proteins from Cynanchum thesioides
Source: PeerJ. 2026 Apr 15;14:e20998. doi: 10.7717/peerj.20998 (PMC13091576; doi:10.7717/peerj.20998)

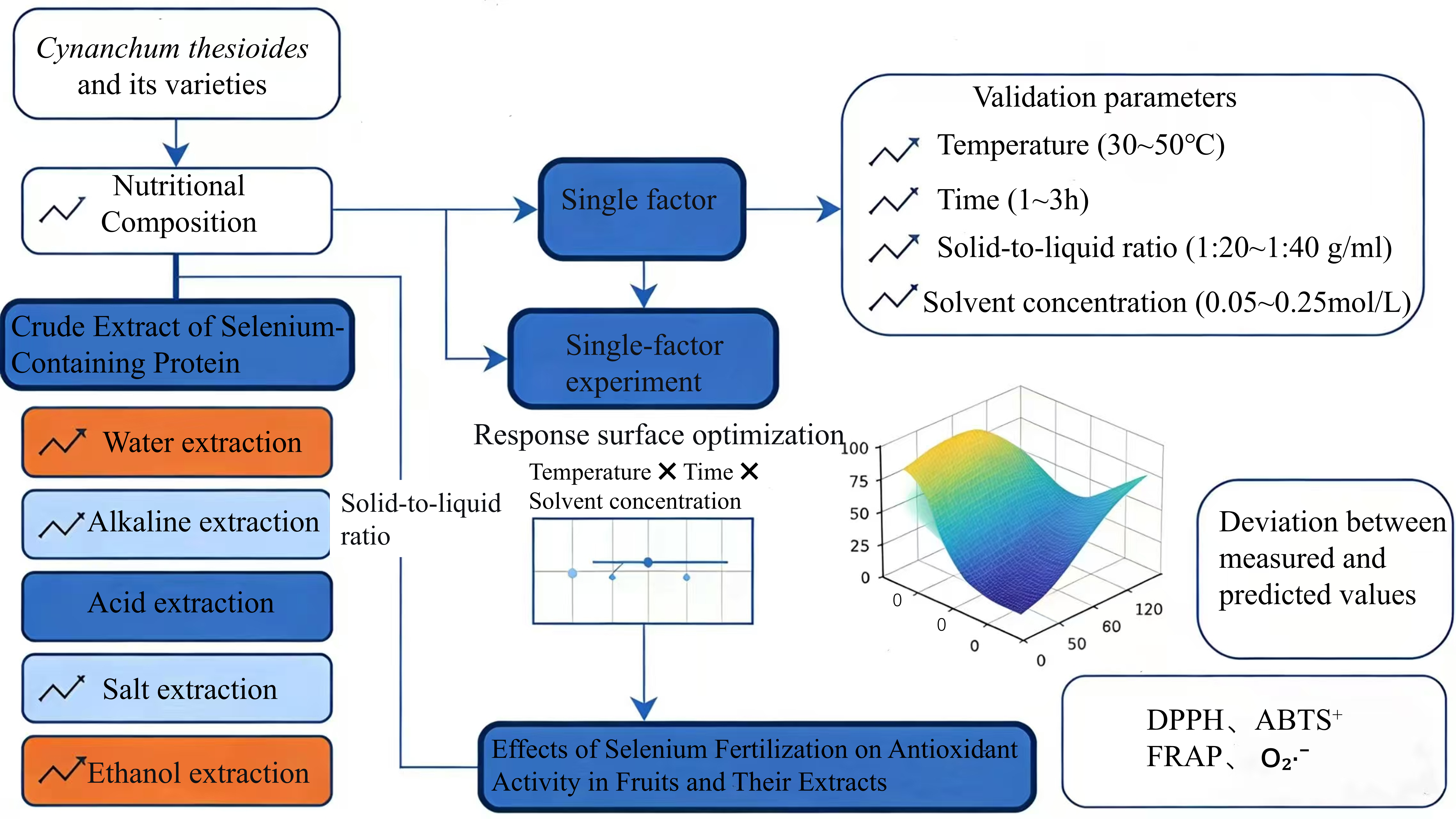

Supplement: Supplemental Information 1 [file peerj-14-20998-s001.png]

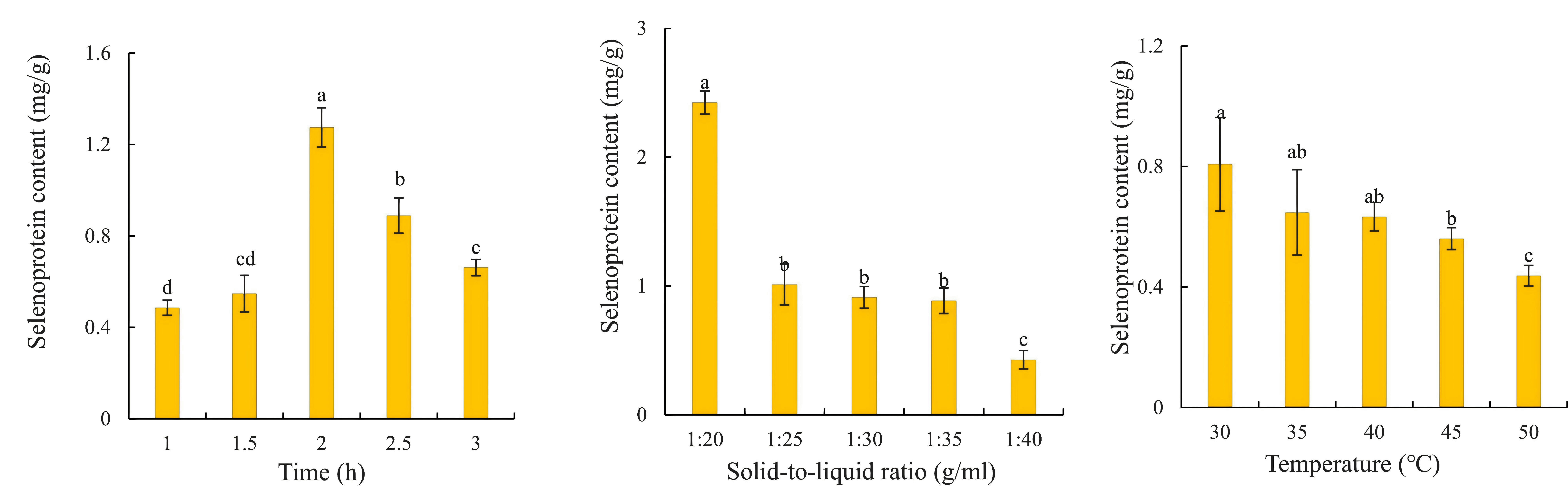

Supplement: Supplemental Information 2 — The horizontal axis represents the four factors, namely time, temperature, solid-to-liquid ratio, and solvent concentration, respectively. The vertical axis shows the selenium content (mg/g). Different lowercase letters above the bars indicate significant differences among the treatments at the 0.05 level. [file peerj-14-20998-s002.png]

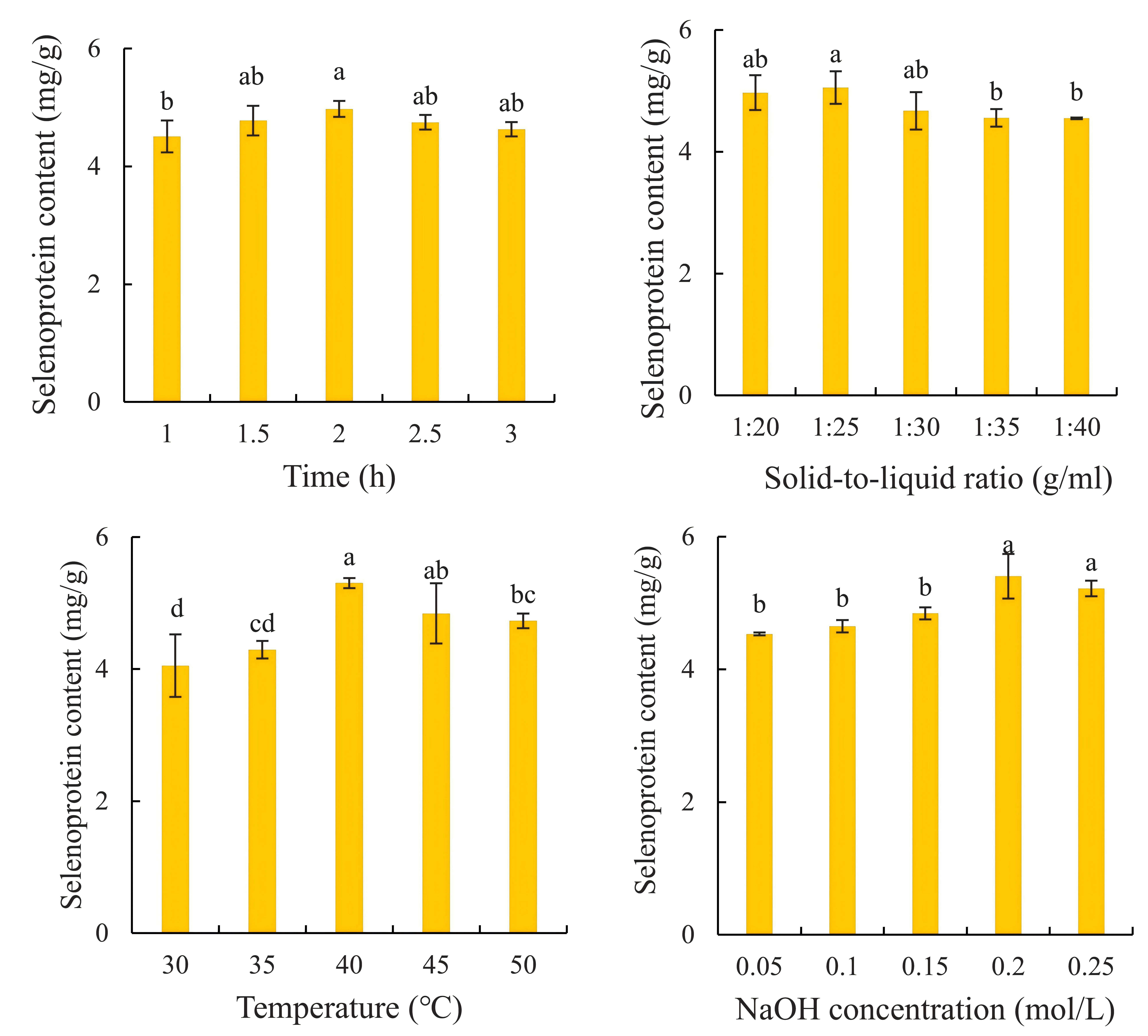

Supplement: Supplemental Information 3 — The horizontal axis represents the four factors, namely time, temperature, solid-to-liquid ratio, and solvent concentration, respectively. The vertical axis shows the selenium content (mg/g). Different lowercase letters above the bars indicate significant differences among the treatments at the 0.05 level. [file peerj-14-20998-s003.png]

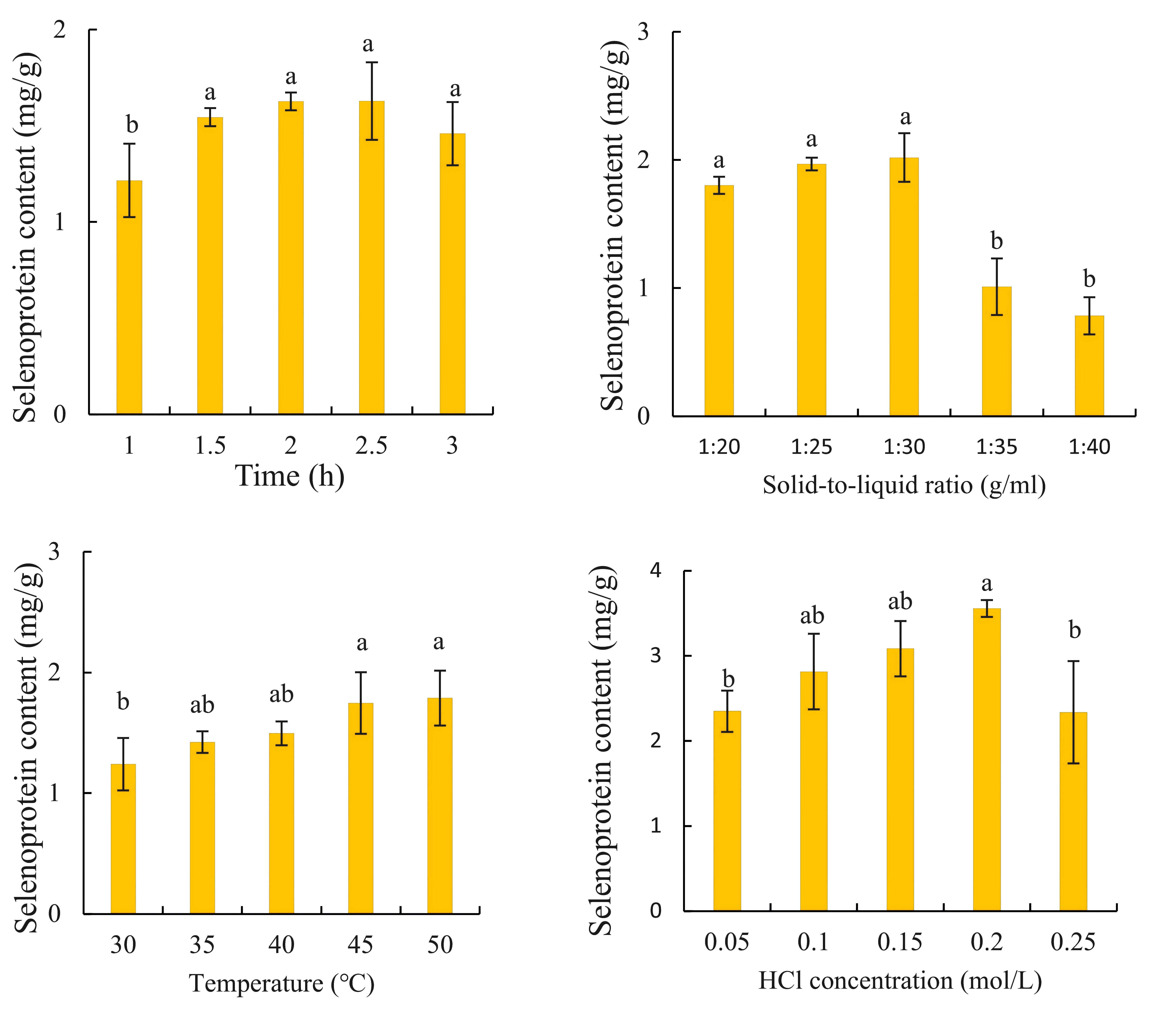

Supplement: Supplemental Information 4 — The horizontal axis represents the four factors, namely time, temperature, solid-to-liquid ratio, and solvent concentration, respectively. The vertical axis shows the selenium content (mg/g). Different lowercase letters above the bars indicate significant differences among the treatments at the 0.05 level. [file peerj-14-20998-s004.png]

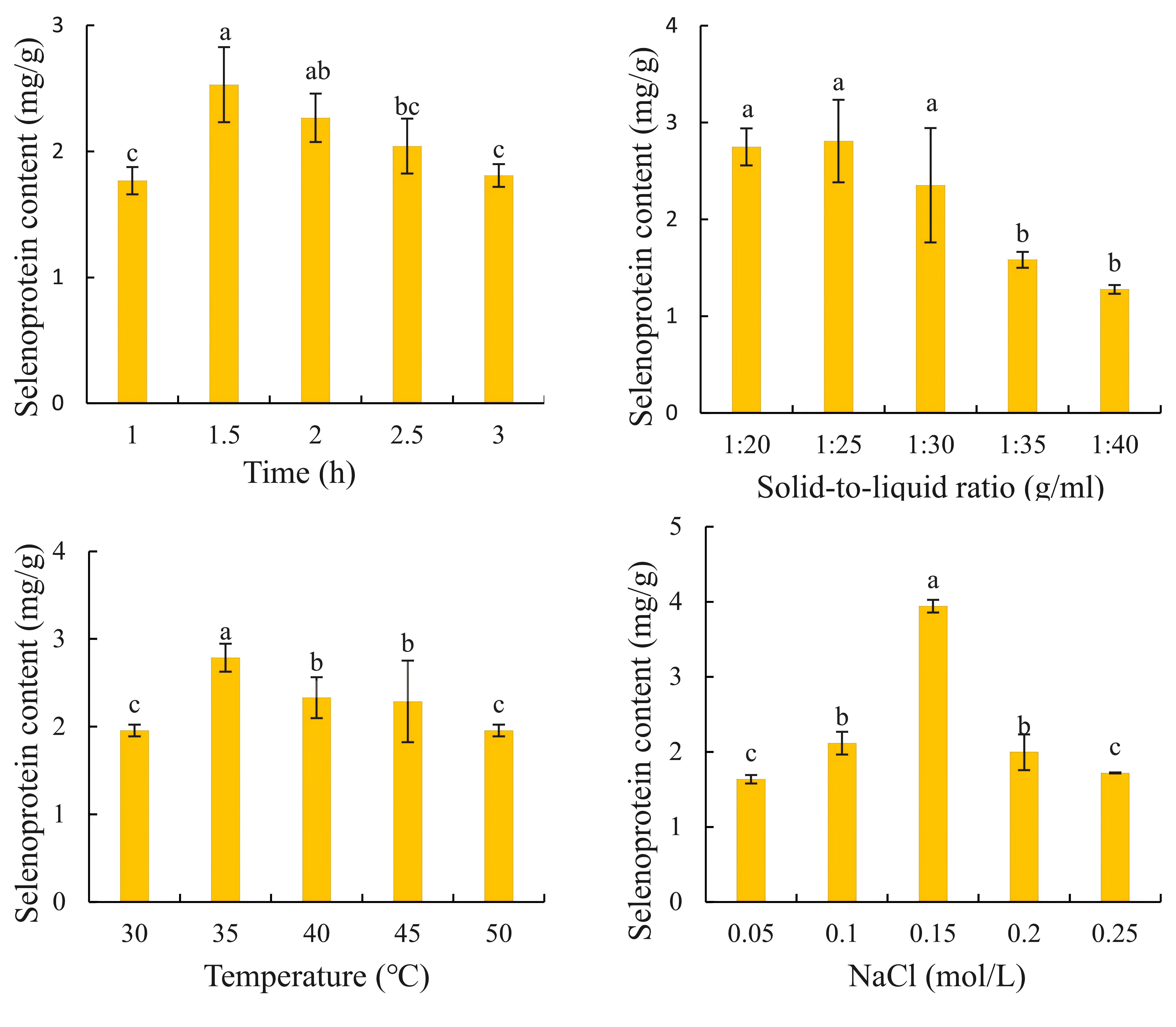

Supplement: Supplemental Information 5 — The horizontal axis represents the four factors, namely time, temperature, solid-to-liquid ratio, and solvent concentration, respectively. The vertical axis shows the selenium content (mg/g). Different lowercase letters above the bars indicate significant differences among the treatments at the 0.05 level. [file peerj-14-20998-s005.png]

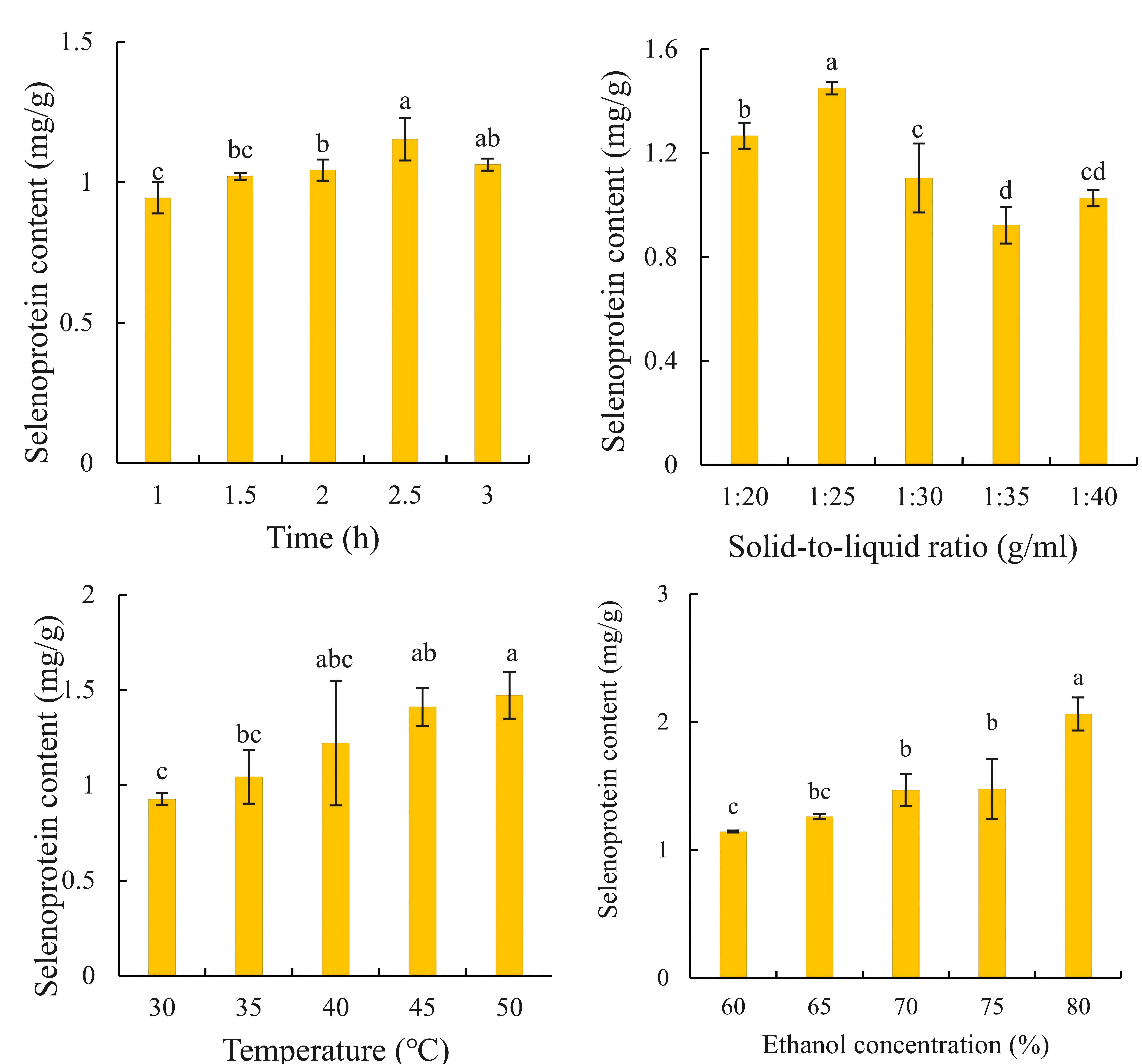

Supplement: Supplemental Information 6 — The horizontal axis represents the four factors, namely time, temperature, solid-to-liquid ratio, and solvent concentration, respectively. The vertical axis shows the selenium content (mg/g). Different lowercase letters above the bars indicate significant differences among the treatments at the 0.05 level. [file peerj-14-20998-s006.png]

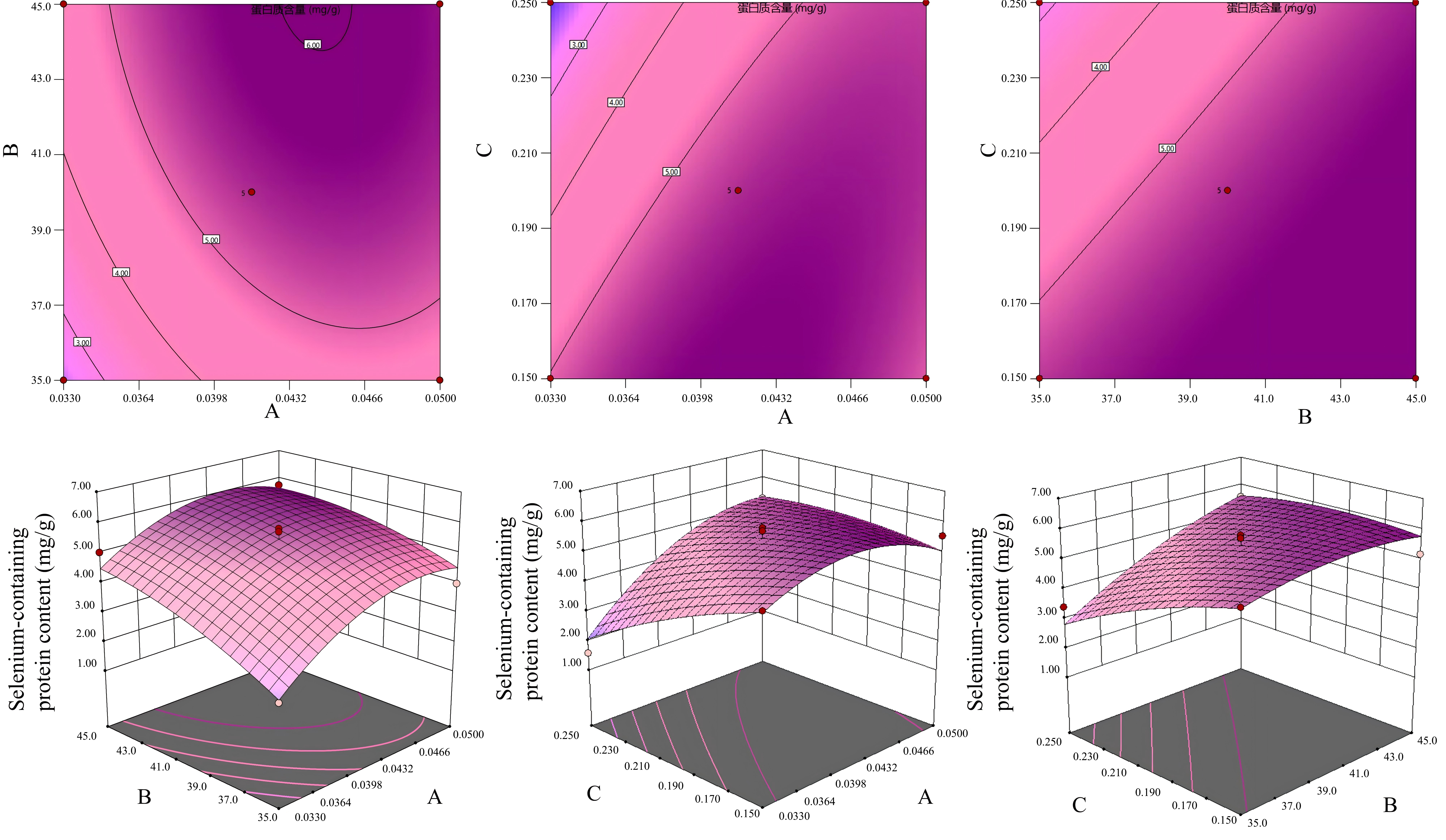

Supplement: Supplemental Information 7 — A, B, and C represent the solid-to-liquid ratio, temperature, and solvent concentration, respectively. [file peerj-14-20998-s007.png]

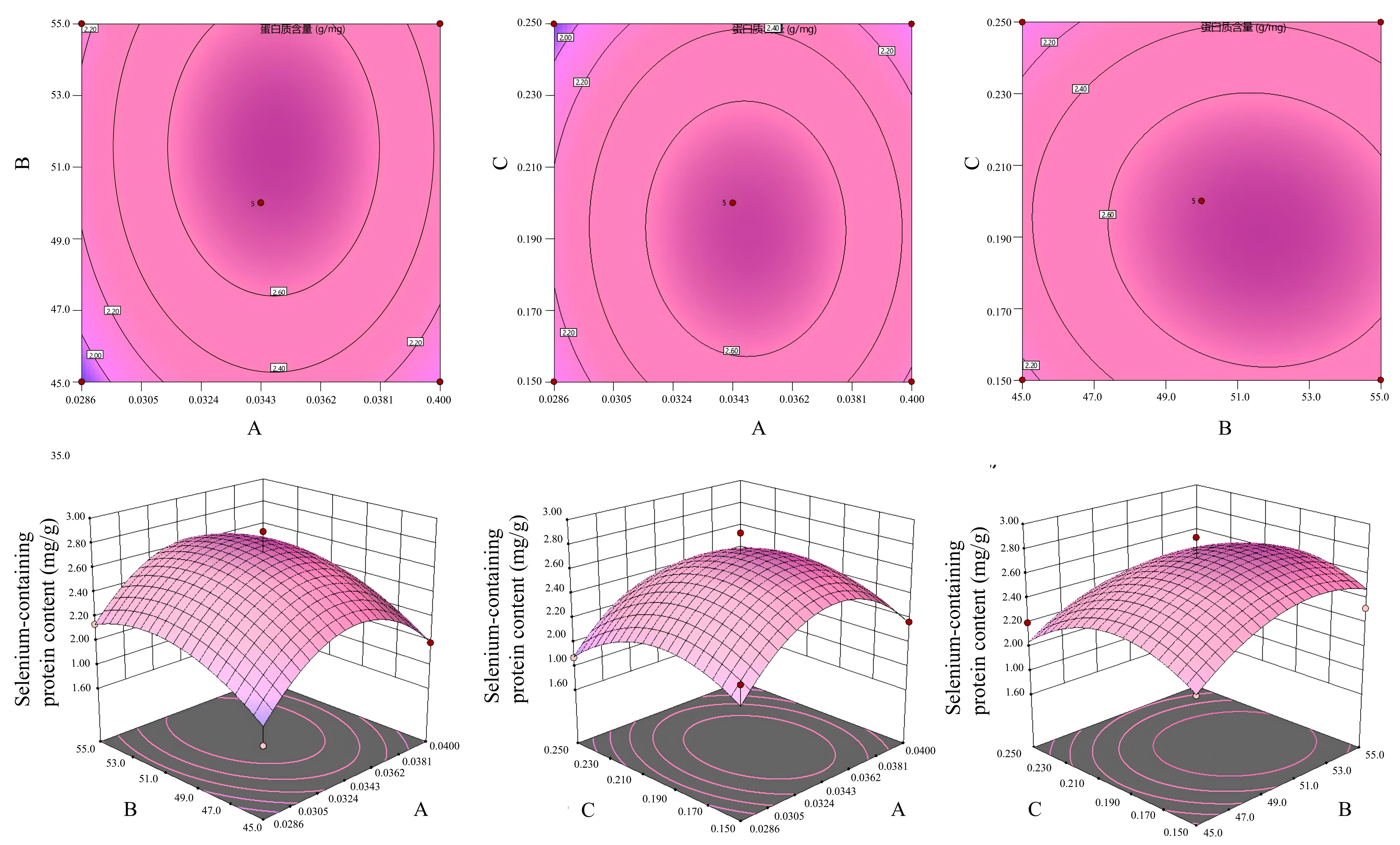

Supplement: Supplemental Information 8 — A, B, and C represent the solid-to-liquid ratio, temperature, and solvent concentration, respectively. [file peerj-14-20998-s008.png]

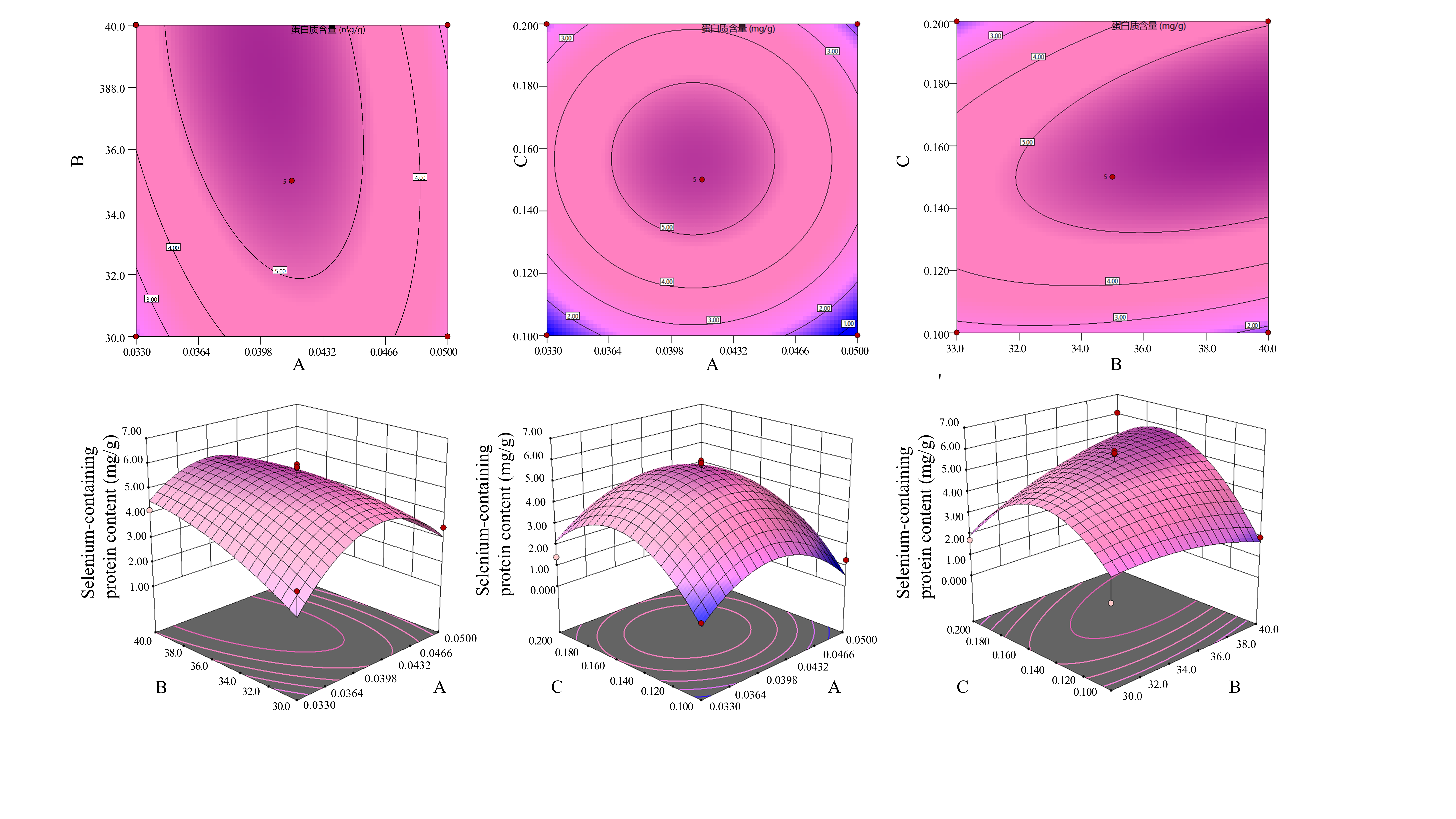

Supplement: Supplemental Information 9 — A, B, and C represent the solid-to-liquid ratio, temperature, and solvent concentration, respectively. [file peerj-14-20998-s009.png]

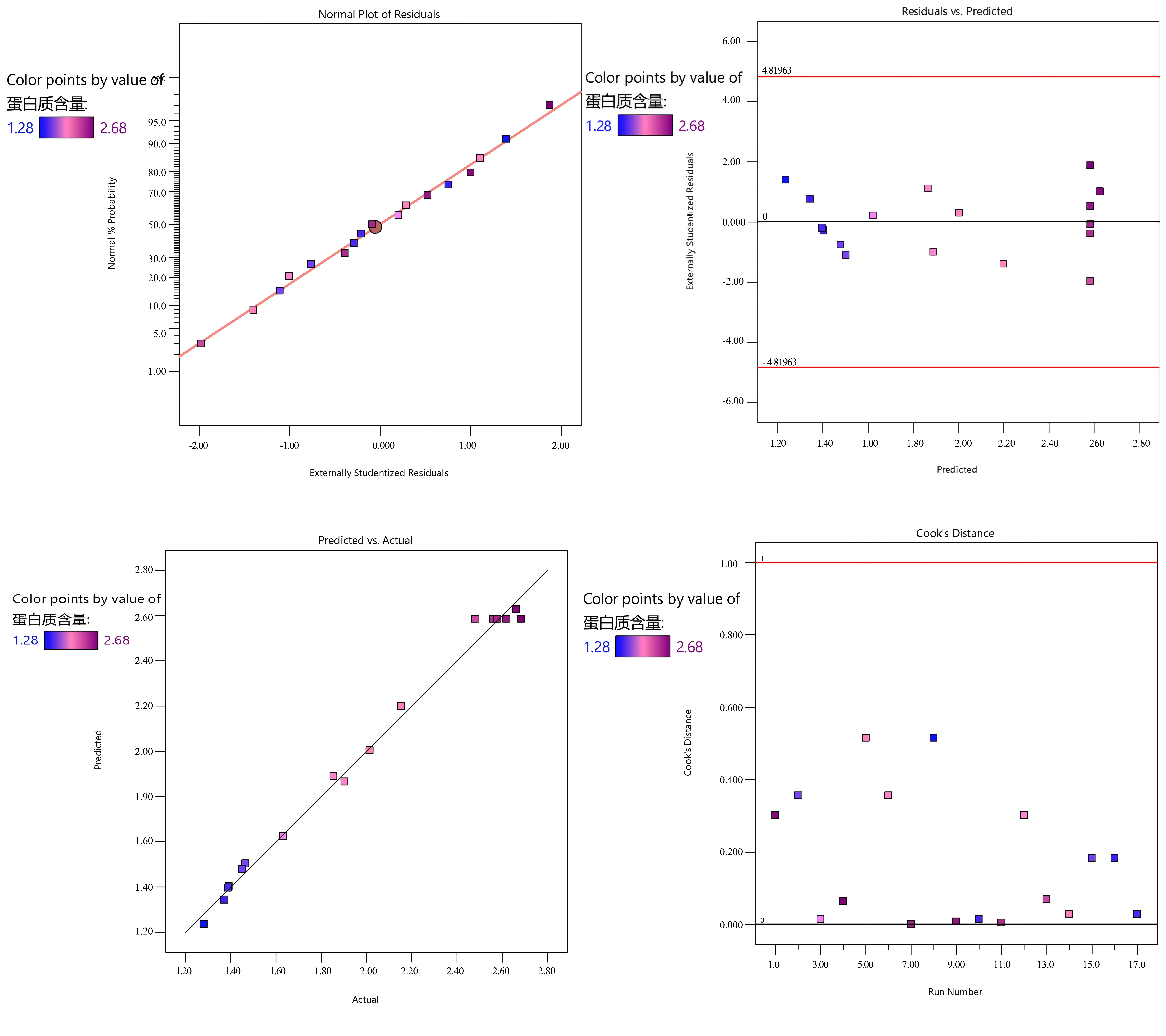

Supplement: Supplemental Information 10 — The four images are: Normal Probability Plot of Residuals, Plot of Residuals vs Predicted Values, Plot of Predicted Values vs Actual Values, and Cook’s Distance Plot. [file peerj-14-20998-s010.png]

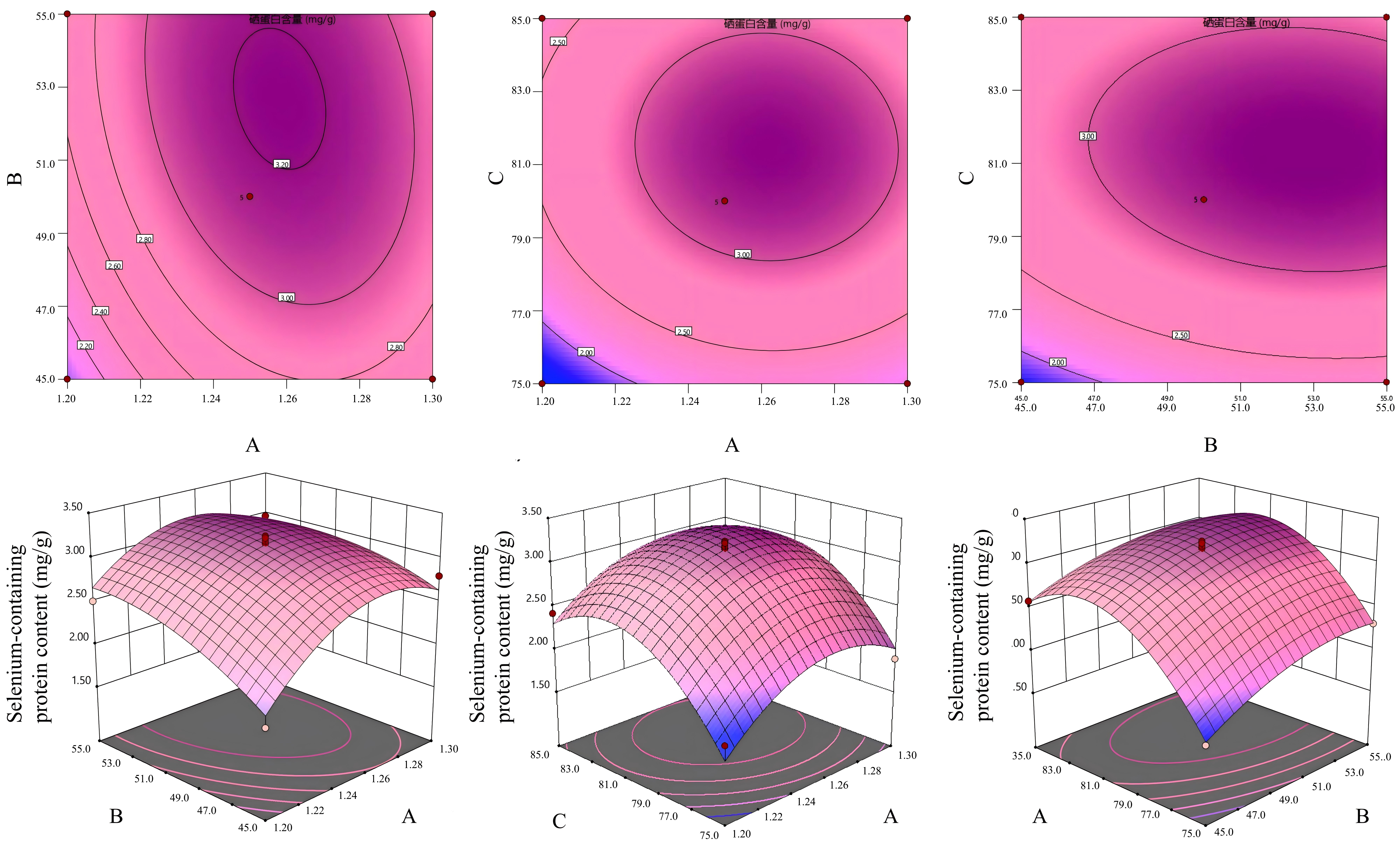

Supplement: Supplemental Information 11 — A, B, and C represent the solid-to-liquid ratio, temperature, and solvent concentration, respectively. [file peerj-14-20998-s011.png]

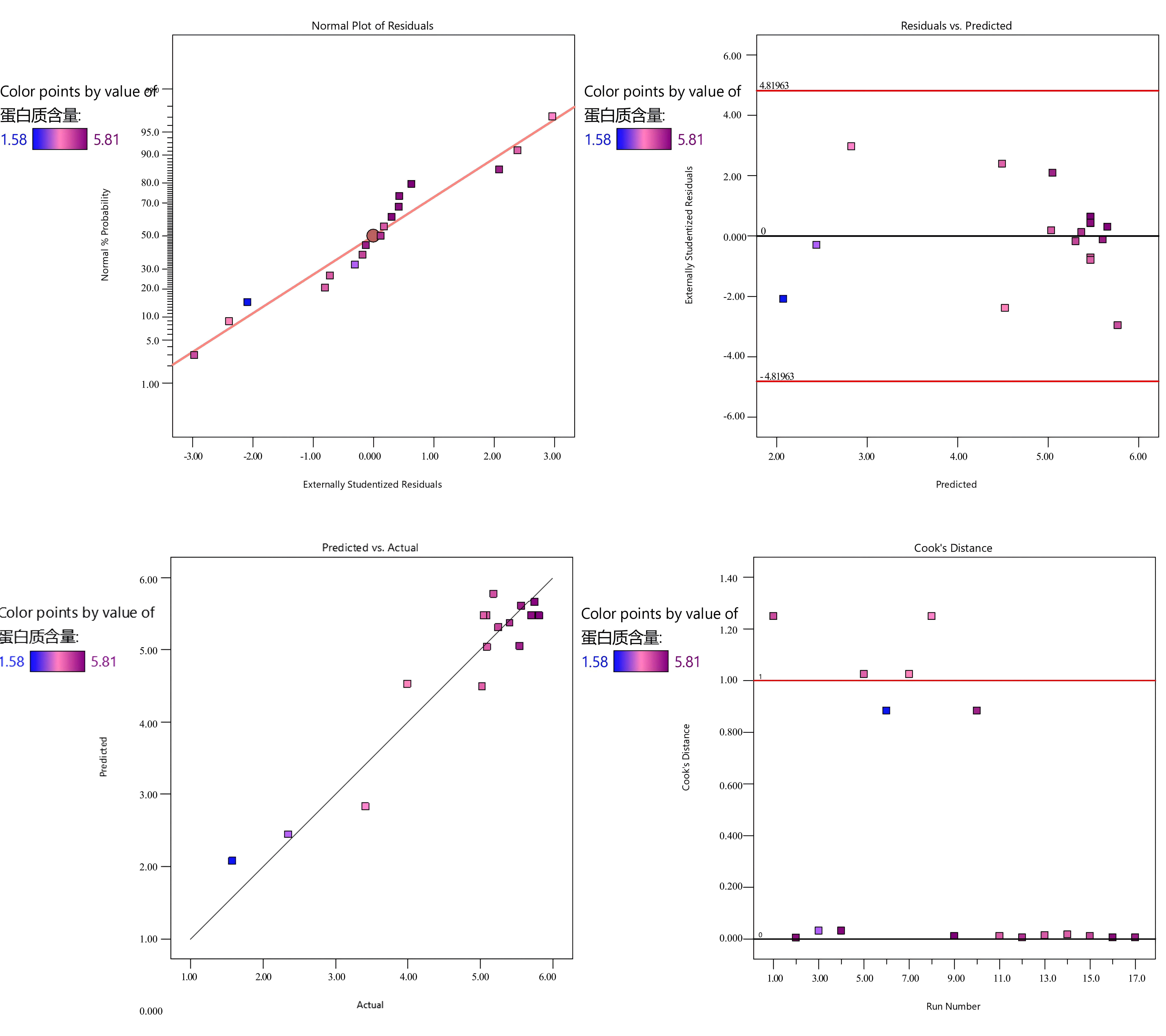

Supplement: Supplemental Information 12 — The four images are: Normal Probability Plot of Residuals, Plot of Residuals vs Predicted Values, Plot of Predicted Values vs Actual Values, and Cook’s Distance Plot. [file peerj-14-20998-s012.png]

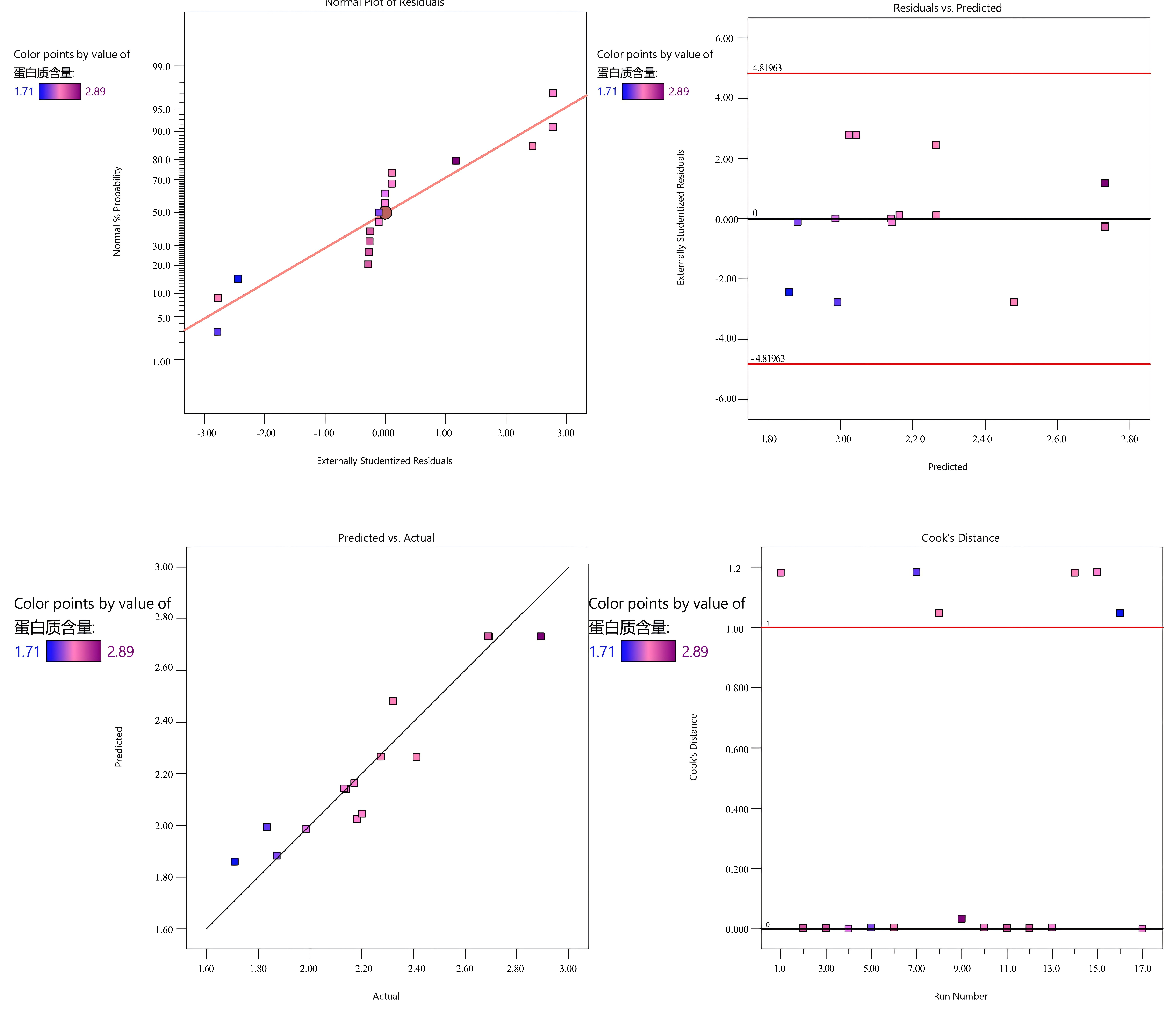

Supplement: Supplemental Information 13 — The four images are: Normal Probability Plot of Residuals, Plot of Residuals vs Predicted Values, Plot of Predicted Values vs Actual Values, and Cook’s Distance Plot. [file peerj-14-20998-s013.png]

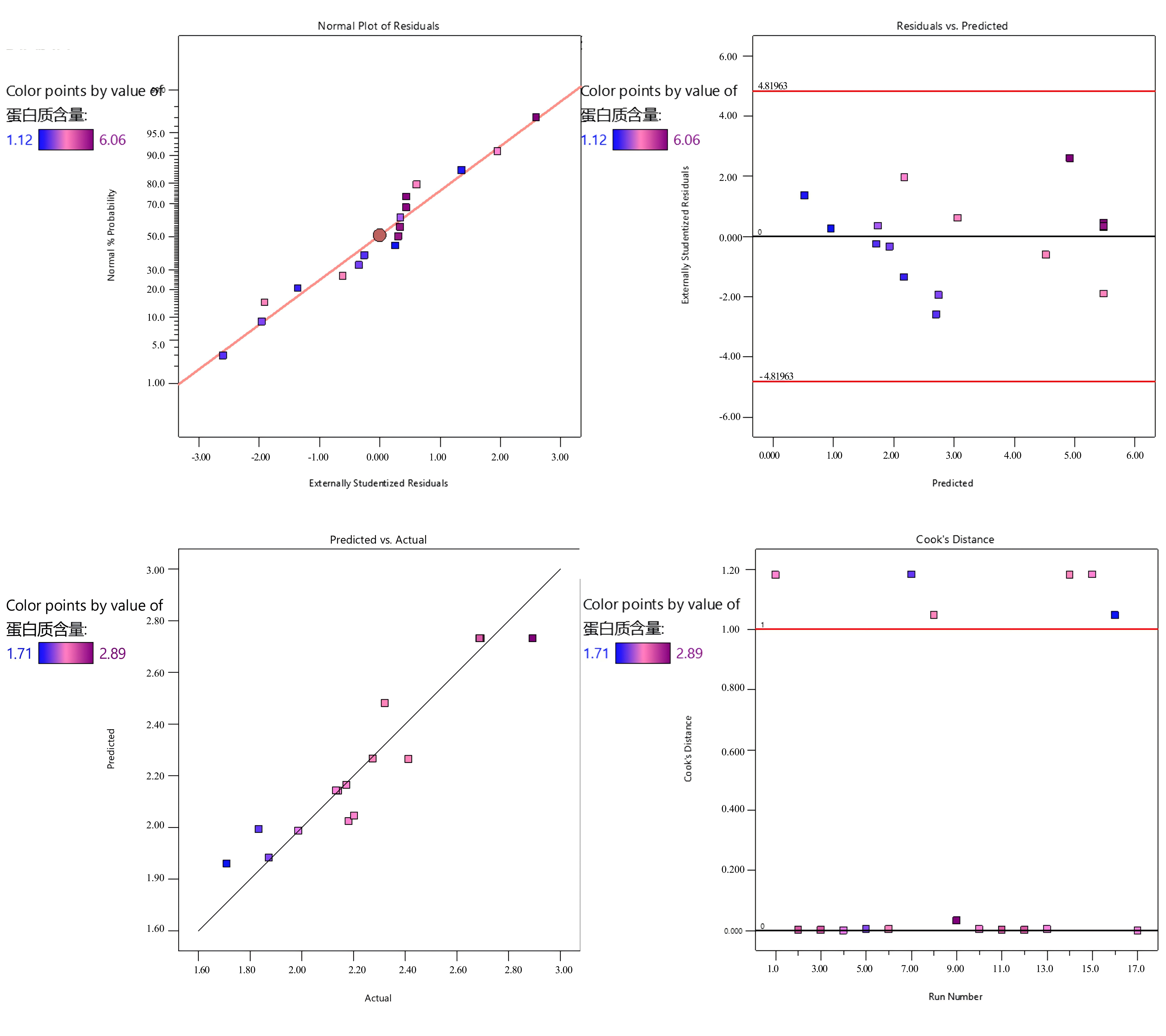

Supplement: Supplemental Information 14 — The four images are: Normal Probability Plot of Residuals, Plot of Residuals vs Predicted Values, Plot of Predicted Values vs Actual Values, and Cook’s Distance Plot. [file peerj-14-20998-s014.png]

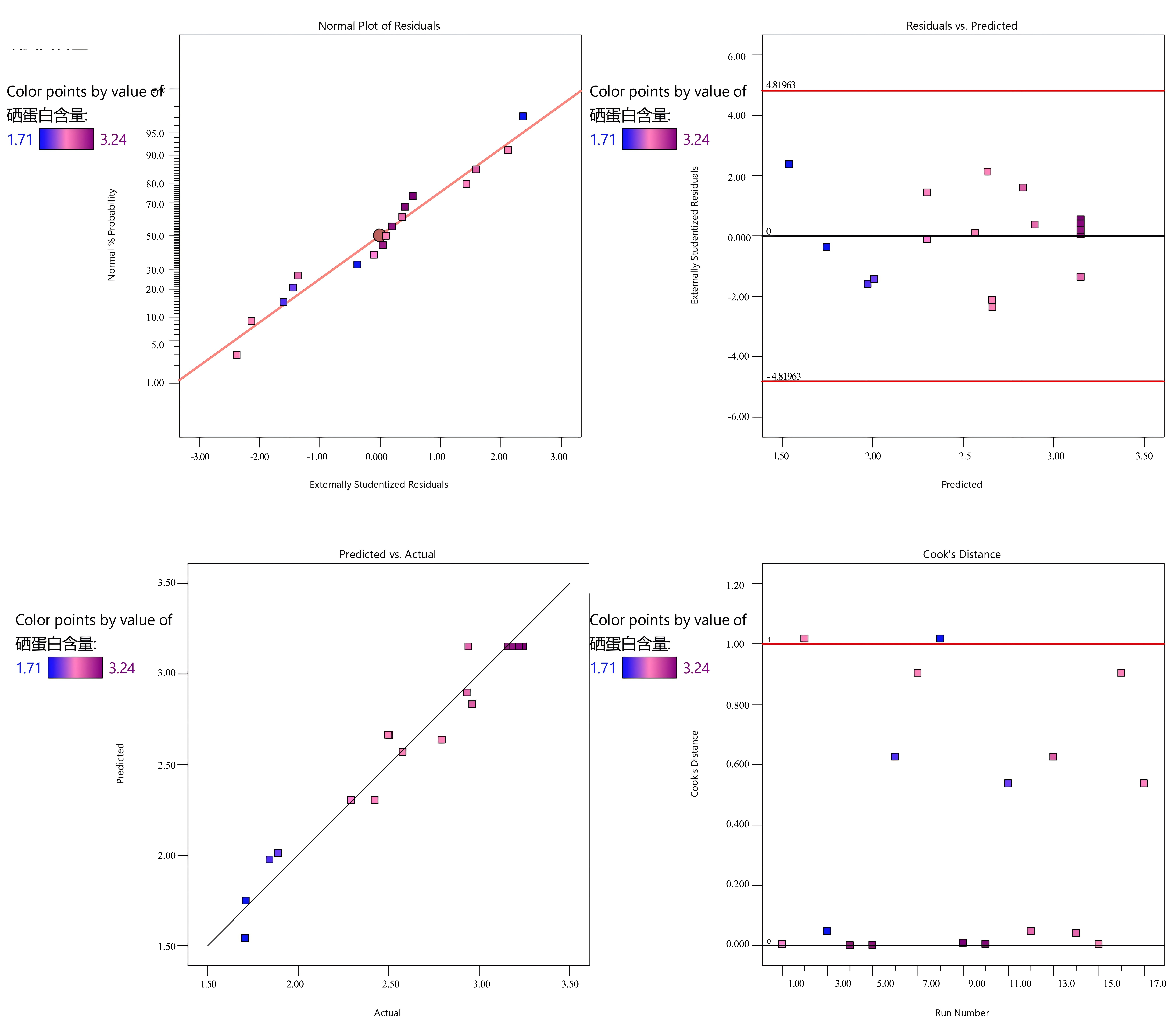

Supplement: Supplemental Information 15 — The four images are: Normal Probability Plot of Residuals, Plot of Residuals vs Predicted Values, Plot of Predicted Values vs Actual Values, and Cook’s Distance Plot. [file peerj-14-20998-s015.png]

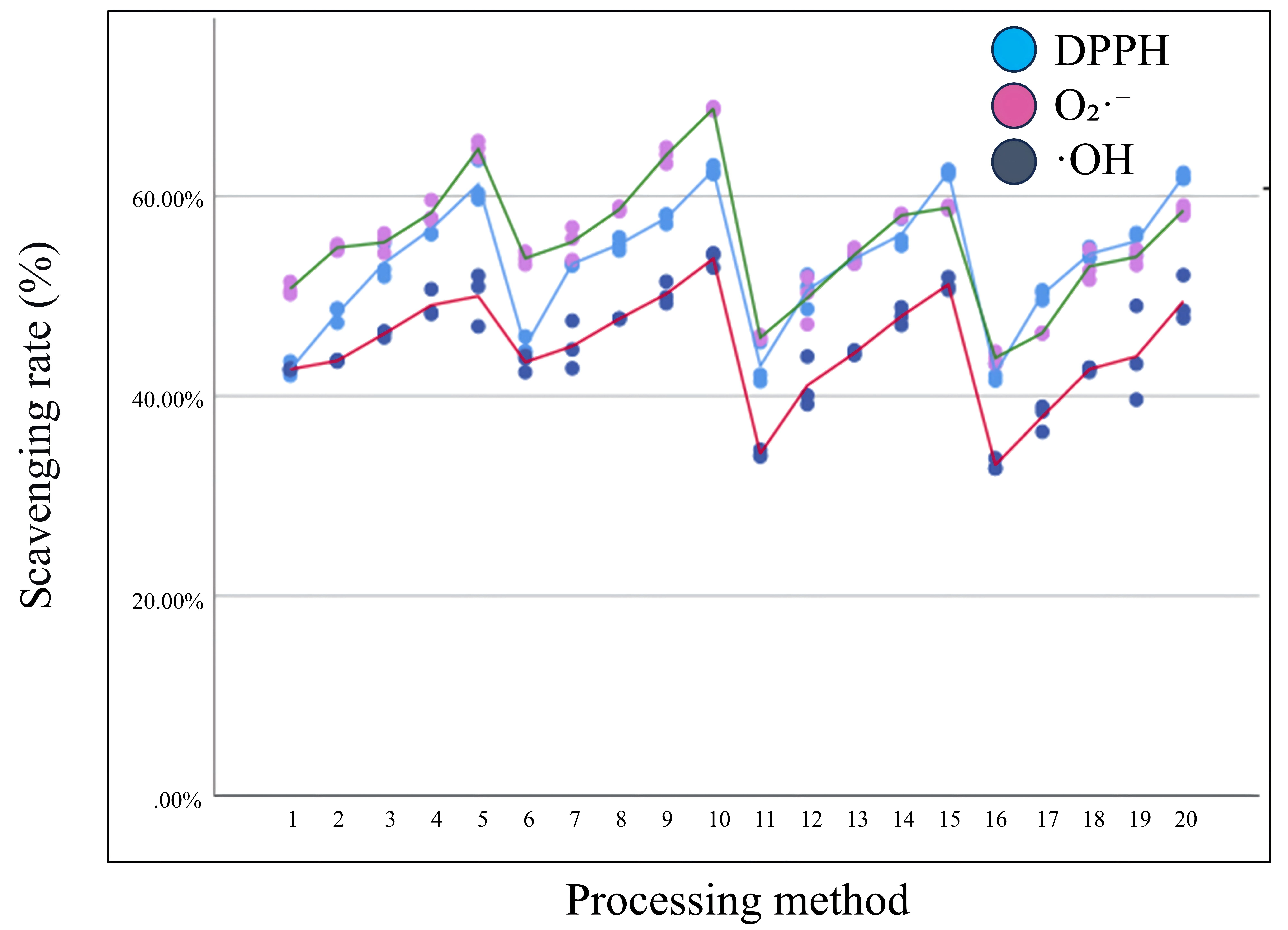

Supplement: Supplemental Information 16 — The x-axis represents the treatment method, and the y-axis represents the clearance rate (%).1-5: The selenium fertilizer concentration is 0 mg/L, and the extract concentrations are 1, 2, 3, 4, and 5 mg/mL respectively; 6-10: The selenium fertilizer concentration is 2 mg/L, and the extract concentrations are 1, 2, 3, 4, and 5 mg/mL respectively; 11-15: The selenium fertilizer concentration is 4 mg/L, and the extract concentrations are 1, 2, 3, 4, and 5 mg/mL respectively; 16-20: The selenium fertilizer concentration is 0 mg/L, and the extract concentrations are 1, 2, 3, 4, and 5 mg/mL respectively. [file peerj-14-20998-s016.png]
