# Supplementary material for: Optimization of the extraction process and in vitro antioxidant capacity analysis of selenium-containing proteins from Cynanchum thesioides
Source: PeerJ. 2026 Apr 15;14:e20998. doi: 10.7717/peerj.20998 (PMC13091576; doi:10.7717/peerj.20998)
Supplement: Supplemental Information 17 [file peerj-14-20998-s017.docx]

**Table S3-1** Coefficients of coded factors for the water extraction method

| Factor | Coefficient Estimate | df | Standard Error | 95% CI Low | 95% CI High | VIF |
| --- | --- | --- | --- | --- | --- | --- |
| Intercept | 2.58 | 1 | 0.0309 | 2.51 | 2.66 | 1.00 |
| A-A temperature | 0.0935 | 1 | 0.0244 | 0.0358 | 0.151 | 1.00 |
| B-B time | 0.275 | 1 | 0.0244 | 0.217 | 0.333 | 1.00 |
| C-C solid-to-liquid ratio | -0.207 | 1 | 0.0244 | -0.265 | -0.149 | 1.00 |
| AB | 0.287 | 1 | 0.0345 | 0.205 | 0.369 | 1.00 |
| AC | -0.0965 | 1 | 0.0345 | -0.178 | -0.0148 | 1.00 |
| BC | -0.153 | 1 | 0.0345 | -0.235 | -0.0716 | 1.00 |
| A² | -0.286 | 1 | 0.0337 | -0.365 | -0.206 | 1.01 |
| B² | -0.328 | 1 | 0.0337 | -0.408 | -0.248 | 1.01 |
| C² | -0.692 | 1 | 0.0337 | -0.772 | -0.613 | 1.01 |

**Table S3-2** Coefficients of coded factors for the alkaline extraction method

| Factor | Coefficient Estimate | df | Standard Error | 95% CI Low | 95% CI High | VIF |
| --- | --- | --- | --- | --- | --- | --- |
| Intercept | 5.47 | 1 | 0.2578 | 4.86 | 6.08 | 1.00 |
| A-A solid-to-liquid ratio | 0.811 | 1 | 0.2038 | 0.3294 | 1.293 | 1.00 |
| B-B temperature | 0.795 | 1 | 0.2038 | 0.313 | 1.277 | 1.00 |
| C-C concentration | -0.677 | 1 | 0.2038 | -1.159 | -0.194 | 1.00 |
| AB | -0.230 | 1 | 0.2883 | -0.911 | 0.4519 | 1.00 |
| AC | 0.8045 | 1 | 0.2883 | 0.123 | 1.4862 | 1.00 |
| BC | 0.594 | 1 | 0.2883 | -0.088 | 1.2756 | 1.00 |
| A² | -0.859 | 1 | 0.2809 | -1.525 | -0.195 | 1.01 |
| B² | -0.332475 | 1 | 0.2810 | -0.997 | 0.332 | 1.01 |
| C² | -0.245225 | 1 | 0.2810 | -0.910 | 0.419 | 1.01 |

**Table S3-3** Coefficients of coded factors for the acid extraction method

| Factor | Coefficient Estimate | df | Standard Error | 95% CI Low | 95% CI High | VIF |
| --- | --- | --- | --- | --- | --- | --- |
| Intercept | 2.73 | 1 | 0.0565 | -0.0709 | 0.196 | 1.00 |
| A-A solid-to-liquid ratio | 0.0626 | 1 | 0.0565 | 0.00626 | 0.273 | 1.00 |
| B-B temperature | 0.140 | 1 | 0.0565 | -0.212 | 0.0554 | 1.00 |
| C-C concentration | -0.0781 | 1 | 0.0798 | -0.190 | 0.188 | 1.00 |
| AB | -0.00125 | 1 | 0.0798 | -0.196 | 0.181 | 1.00 |
| AC | -0.00750 | 1 | 0.0798 | -0.218 | 0.160 | 1.00 |
| BC | -0.0292 | 1 | 0.0778 | -0.627 | -0.259 | 1.00 |
| A² | -0.443 | 1 | 0.0778 | -0.409 | -0.0413 | 1.01 |
| B² | -0.225 | 1 | 0.0778 | -0.456 | -0.0885 | 1.01 |
| C² | -0.272 | 1 | 0.0565 | -0.0709 | 0.196 | 1.01 |

**Table S3-4** Coefficients of coded factors for the salt extraction method

| Factor | Coefficient Estimate | df | Standard Error | 95% CI Low | 95% CI High | VIF |
| --- | --- | --- | --- | --- | --- | --- |
| Intercept | 5.48 | 1 | 0.526 | 4.24 | 6.73 | 1.00 |
| A-A solid-to-liquid ratio | -0.224 | 1 | 0.416 | -1.21 | 0.760 | 1.00 |
| B-B temperature | 0.506 | 1 | 0.416 | -0.477 | 1.49 | 1.00 |
| C-C concentration | 0.602 | 1 | 0.416 | -0.382 | 1.59 | 1.00 |
| AB | -0.666 | 1 | 0.588 | -2.06 | 0.726 | 1.00 |
| AC | -0.00350 | 1 | 0.588 | -1.39 | 1.39 | 1.00 |
| BC | 0.989 | 1 | 0.588 | -0.403 | 2.38 | 1.00 |
| A² | -1.92 | 1 | 0.573 | -3.28 | -0.564 | 1.01 |
| B² | -0.436 | 1 | 0.573 | -1.79 | 0.920 | 1.01 |
| C² | -2.22 | 1 | 0.573 | -3.58 | -0.864 | 1.01 |

**Table S3-5** Coefficients of coded factors for the ethanol extraction method

| Factor | Coefficient Estimate | df | Standard Error | 95% CI Low | 95% CI High | VIF |
| --- | --- | --- | --- | --- | --- | --- |
| Intercept | 3.15 | 1 | 0.0812 | 2.96 | 3.34 | 1.00 |
| A-A solid-to-liquid ratio | 0.208 | 1 | 0.0642 | 0.0560 | 0.360 | 1.00 |
| B-B temperature | 0.221 | 1 | 0.0642 | 0.0691 | 0.373 | 1.00 |
| C-C concentration | 0.354 | 1 | 0.0642 | 0.202 | 0.506 | 1.00 |
| AB | -0.123 | 1 | 0.0908 | -0.338 | 0.0915 | 1.00 |
| AC | -0.0275 | 1 | 0.0908 | -0.242 | 0.187 | 1.00 |
| BC | -0.0568 | 1 | 0.0908 | -0.272 | 0.158 | 1.00 |
| A² | -0.437 | 1 | 0.0885 | -0.646 | -0.228 | 1.01 |
| B² | -0.187 | 1 | 0.0885 | -0.397 | 0.0220 | 1.01 |
| C² | -0.585 | 1 | 0.0885 | -0.794 | -0.375 | 1.01 |
