# Supplementary material for: Optimization of the extraction process and in vitro antioxidant capacity analysis of selenium-containing proteins from Cynanchum thesioides
Source: PeerJ. 2026 Apr 15;14:e20998. doi: 10.7717/peerj.20998 (PMC13091576; doi:10.7717/peerj.20998)
Supplement: Supplemental Information 18 [file peerj-14-20998-s018.docx]

**Table S2-1** Analysis of variance (ANOVA) for the response surface regression model of water extraction of selenium-containing proteins from *C. thesioides* fruits

| Source | Sum of Squares | Degrees of Freedom | Mean Square | F-value | P-value |
| --- | --- | --- | --- | --- | --- |
| Model | 4.55 | 9 | 0.5059 | 106.02 | < 0.0001 |
| A (Temperature) | 0.0699 | 1 | 0.0699 | 14.66 | 0.0065 |
| B (Time) | 0.6045 | 1 | 0.6045 | 126.68 | < 0.0001 |
| C (Solid-to-liquid ratio) | 0.3424 | 1 | 0.3424 | 71.75 | < 0.0001 |
| AB | 0.3295 | 1 | 0.3295 | 69.05 | < 0.0001 |
| AC | 0.0372 | 1 | 0.0372 | 7.81 | 0.0268 |
| BC | 0.0939 | 1 | 0.0939 | 19.69 | 0.0030 |
| A^2^ | 0.3436 | 1 | 0.3436 | 72.01 | < 0.0001 |
| B^2^ | 0.4528 | 1 | 0.4528 | 94.89 | < 0.0001 |
| C^2^ | 2.02 | 1 | 2.02 | 423.07 | < 0.0001 |
| Residual | 0.0334 | 7 | 0.0048 |  |  |
| Lack of fit | 0.0111 | 3 | 0.0037 | 0.6658 | 0.6155 |
| Pure error | 0.0223 | 4 | 0.0056 |  |  |
| Total | 4.59 | 16 |  |  |  |
| Regression equation | Y=2.58+0.0935*A+0.2749*B+-0.2069*C+0.2870*AB-0.0965*AC-0.1532*BC-0.2857*A^2^-0.3279*B^2^-0.6924*C^2^ | | | | |

**Table S2-2** Analysis of variance (ANOVA) for the response surface regression model of alkaline extraction of selenium-containing proteins from *C. thesioides* fruits

| Source | Sum of Squares | Degrees of Freedom | Mean Square | F-value | P-value |
| --- | --- | --- | --- | --- | --- |
| Model | 22.32 | 9 | 2.48 | 7.46 | 0.0074 |
| A (Solid-to-liquid ratio) | 5.27 | 1 | 5.27 | 15.84 | 0.0053 |
| B (Temperature) | 5.06 | 1 | 5.06 | 15.21 | 0.0059 |
| C (concentration) | 3.66 | 1 | 3.66 | 11.01 | 0.0128 |
| AB | 0.2111 | 1 | 0.2111 | 0.6351 | 0.4517 |
| AC | 2.59 | 1 | 2.59 | 7.79 | 0.0269 |
| BC | 1.41 | 1 | 1.41 | 4.25 | 0.0783 |
| A^2^ | 3.11 | 1 | 3.11 | 9.35 | 0.0184 |
| B^2^ | 0.4654 | 1 | 0.4654 | 1.40 | 0.2753 |
| C^2^ | 0.2532 | 1 | 0.2532 | 0.7617 | 0.4117 |
| Residual | 2.33 | 7 | 0.3324 |  |  |
| Lack of fit | 1.78 | 3 | 0.5918 | 4.29 | 0.0967 |
| Pure error | 0.5517 | 4 | 0.1379 |  |  |
| Total | 24.64 | 16 |  |  |  |
| Regression equation | Y=5.47+0.8114*A+0.7951*B-0.6765*C-0.2298*AB+0.8045*AC+0.594*BC-0.8590*A^2^-0.3325*B^2^-0.2452*C^2^ | | | | |

**Table S2-3** Analysis of variance (ANOVA) for the response surface regression model of acid extraction of selenium-containing proteins from *C. thesioides* fruits

| Source | Sum of Squares | Degrees of Freedom | Mean Square | F-value | P-value |
| --- | --- | --- | --- | --- | --- |
| Model | 1.73 | 9 | 0.1926 | 7.55 | 0.0071 |
| A (Solid-to-liquid ratio) | 0.0314 | 1 | 0.0314 | 1.23 | 0.3039 |
| B (Temperature) | 0.1562 | 1 | 0.1562 | 6.13 | 0.0425 |
| C (concentration) | 0.0488 | 1 | 0.0488 | 1.92 | 0.2089 |
| AB | 6.250E-06 | 1 | 6.250E-06 | 0.0002 | 0.9879 |
| AC | 0.0002 | 1 | 0.0002 | 0.0088 | 0.9278 |
| BC | 0.0034 | 1 | 0.0034 | 0.1342 | 0.7249 |
| A^2^ | 0.8263 | 1 | 0.8263 | 32.41 | 0.0007 |
| B^2^ | 0.2136 | 1 | 0.2136 | 8.38 | 0.0232 |
| C^2^ | 0.3127 | 1 | 0.3127 | 12.26 | 0.0100 |
| Residual | 0.1785 | 7 | 0.0255 |  |  |
| Lack of fit | 0.1452 | 3 | 0.0484 | 5.83 | 0.0608 |
| Pure error | 0.0332 | 4 | 0.0083 |  |  |
| Total | 1.91 | 16 |  |  |  |
| Regression equation | Y=2.73+0.0626*A+0.1398*B-0.07811*C-0.0013*AB-0.00755*AC-0.0292*BC-0.4430*A^2^-0.2253*B^2^-0.2725*C^2^ | | | | |

**Table S2-4** Analysis of variance (ANOVA) for the response surface regression model of salt extraction of selenium-containing proteins from *C. thesioides* fruits

| Source | Sum of Squares | Degrees of Freedom | Mean Square | F-value | P-value |
| --- | --- | --- | --- | --- | --- |
| Model | 51.17 | 9 | 5.69 | 4.11 | 0.0380 |
| A (Solid-to-liquid ratio) | 0.4001 | 1 | 0.4001 | 0.2889 | 0.6076 |
| B (Temperature) | 2.05 | 1 | 2.05 | 1.48 | 0.2630 |
| C (concentration) | 2.90 | 1 | 2.90 | 2.09 | 0.1912 |
| AB | 1.77 | 1 | 1.77 | 1.28 | 0.2951 |
| AC | 0.0000 | 1 | 0.0000 | 0.0000 | 0.9954 |
| BC | 3.91 | 1 | 3.91 | 2.82 | 0.1368 |
| A^2^ | 15.52 | 1 | 15.52 | 11.21 | 0.0123 |
| B^2^ | 0.8018 | 1 | 0.8018 | 0.5790 | 0.4716 |
| C^2^ | 20.74 | 1 | 20.74 | 14.98 | 0.00613 |
| Residual | 9.69 | 7 | 1.38 |  |  |
| Lack of fit | 6.02 | 3 | 2.01 | 2.1 | 0.2327 |
| Pure error | 3.68 | 4 | 0.9190 |  |  |
| Total | 60.86 | 16 |  |  |  |
| Regression equation | Y=-87.944+2728.72*A+1.380*B+140.347*C-15.665*AB-8.235*AC+3.95400*BC-26572.664*A^2^-0.018*B^2^-887.850*C^2^ | | | | |

**Table S2-5** Analysis of variance (ANOVA) for the response surface regression model of organic solvent extraction of selenium-containing proteins from *C. thesioides* fruits

| Source | Sum of Squares | Degrees of Freedom | Mean Square | F-value | P-value |
| --- | --- | --- | --- | --- | --- |
| Model | 4.43 | 9 | 0.49 | 14.91 | 0.0008 |
| A (Solid-to-liquid ratio) | 0.3457 | 1 | 0.3457 | 10.4755 | 0.0143 |
| B (Temperature) | 0.39 | 1 | 0.39 | 11.84 | 0.0108 |
| C (concentration) | 1.00 | 1 | 1.00 | 30.36 | 0.0008 |
| AB | 0.06 | 1 | 0.06 | 1.84 | 0.2169 |
| AC | 0.0030 | 1 | 0.0030 | 0.0917 | 0.7708 |
| BC | 0.0129 | 1 | 0.0129 | 0.3903 | 0.5519 |
| A^2^ | 0.80 | 1 | 0.80 | 24.37 | 0.00168 |
| B^2^ | 0.1477 | 1 | 0.1477 | 4.4760 | 0.0721 |
| C^2^ | 1.4387 | 1 | 1.4387 | 43.5973 | 0.00030 |
| Residual | 0.231 | 7 | 0.0330 |  |  |
| Lack of fit | 0.172 | 3 | 0.0574 | 3.92 | 0.110 |
| Pure error | 0.0587 | 4 | 0.0147 |  |  |
| Total | 4.66 | 16 |  |  |  |
| Regression equation | Y=3.15+0.208*A+0.221*B+0.354*C-0.123*AB-0.0275*AC-0.0568*BC-0.437*A^2^-0.187*B^2^-0.585*C^2^ | | | | |
