# Supplementary material for: Optimization of the extraction process and in vitro antioxidant capacity analysis of selenium-containing proteins from Cynanchum thesioides
Source: PeerJ. 2026 Apr 15;14:e20998. doi: 10.7717/peerj.20998 (PMC13091576; doi:10.7717/peerj.20998)
Supplement: Supplemental Information 19 [file peerj-14-20998-s019.docx]

**Table S1-1** Experimental design and results of response surface methodology (RSM) for water-extractable selenium-containing proteins from *C. thesioides* fruits

| Run Number | Factor | | | Selenoprotein content  (mg/g) |
| --- | --- | --- | --- | --- |
|  | A | B | C |  |
| 1 | 1 | 1 | 0 | 2.661 |
| 2 | 1 | -1 | 0 | 1.465 |
| 3 | -1 | 0 | -1 | 1.631 |
| 4 | 0 | 0 | 0 | 2.684 |
| 5 | 0 | 1 | -1 | 2.154 |
| 6 | -1 | 1 | 0 | 1.903 |
| 7 | 0 | 0 | 0 | 2.579 |
| 8 | 0 | -1 | 1 | 1.281 |
| 9 | 0 | 0 | 0 | 2.619 |
| 10 | 1 | 0 | 1 | 1.389 |
| 11 | 0 | 0 | 0 | 2.559 |
| 12 | -1 | -1 | 0 | 1.855 |
| 13 | 0 | 0 | 0 | 2.482 |
| 14 | 1 | 0 | -1 | 2.014 |
| 15 | 0 | 1 | 1 | 1.452 |
| 16 | 0 | -1 | -1 | 1.370 |
| 17 | -1 | 0 | 1 | 1.392 |

Note: A: Temperature (°C); B: Time (h); C: Solid-to-Liquid Ratio (g/ml).

**Table S1-2** Experimental design and results of response surface methodology (RSM) for alkaline-extracted selenium-containing proteins from *C. thesioides* fruits

| Run Number | Factor | | | Selenoprotein content  (mg/g) |
| --- | --- | --- | --- | --- |
|  | A | B | C |  |
| 1 | 0 | 1 | -1 | 5.183 |
| 2 | 0 | 0 | 0 | 5.702 |
| 3 | -1 | -1 | 0 | 2.350 |
| 4 | 1 | 1 | 0 | 5.750 |
| 5 | -1 | 1 | 0 | 5.026 |
| 6 | -1 | 0 | 1 | 1.580 |
| 7 | 1 | -1 | 0 | 3.993 |
| 8 | 0 | -1 | 1 | 3.416 |
| 9 | 0 | 0 | 0 | 5.812 |
| 10 | 1 | 0 | -1 | 5.545 |
| 11 | -1 | 0 | -1 | 5.092 |
| 12 | 0 | -1 | -1 | 5.407 |
| 13 | 0 | 0 | 0 | 5.087 |
| 14 | 0 | 0 | 0 | 5.048 |
| 15 | 1 | 0 | 1 | 5.251 |
| 16 | 0 | 0 | 0 | 5.707 |
| 17 | 0 | 1 | 1 | 5.568 |

Note: A: Solid-to-Liquid Ratio (g/ml); B: Temperature (°C); C: Solvent Concentration (mol/L).

**Table S1-3** Experimental design and results of response surface methodology (RSM) for acid-extracted selenium-containing proteins from *C. thesioides* fruits

| Run Number | Factor | | | Selenoprotein content  (mg/g) |
| --- | --- | --- | --- | --- |
|  | A | B | C |  |
| 1 | 0 | -1 | 1 | 2.203 |
| 2 | 0 | 0 | 0 | 2.689 |
| 3 | 0 | 0 | 0 | 2.693 |
| 4 | 1 | -1 | 0 | 1/987 |
| 5 | -1 | 0 | 1 | 1.873 |
| 6 | 0 | 1 | 1 | 2.275 |
| 7 | 0 | 0 | 1 | 1.834 |
| 8 | 0 | 1 | 0 | 2.413 |
| 9 | 0 | 0 | 0 | 2.894 |
| 10 | 1 | 0 | -1 | 2.173 |
| 11 | 0 | 0 | 0 | 2.688 |
| 12 | 0 | 0 | 0 | 2.691 |
| 13 | 0 | -1 | -1 | 2.133 |
| 14 | 0 | 1 | -1 | 2.322 |
| 15 | -1 | 0 | -1 | 2.182 |
| 16 | -1 | -1 | 0 | 1.710 |
| 17 | -1 | 1 | 0 | 2.141 |

Note: A: Solid-to-Liquid Ratio (g/ml); B: Temperature (°C); C: Solvent Concentration (mol/L).

**Table S1-4** Experimental design and results of response surface methodology (RSM) for salt-extracted selenium-containing proteins from *C. thesioides* fruits

| Run Number | Factor | | | Selenoprotein content  (mg/g) |
| --- | --- | --- | --- | --- |
|  | A | B | C |  |
| 1 | 0 | -1 | 1 | 1.719 |
| 2 | 0 | 0 | 0 | 5.973 |
| 3 | 0 | 1 | -1 | 1.960 |
| 4 | 1 | 0 | -1 | 1.277 |
| 5 | 0 | -1 | -1 | 1.576 |
| 6 | 1 | -1 | 0 | 3.441 |
| 7 | -1 | 1 | 0 | 4.146 |
| 8 | 0 | 1 | 1 | 6.057 |
| 9 | -1 | 0 | 1 | 1.419 |
| 10 | 0 | 0 | 0 | 5.831 |
| 11 | 1 | 0 | 1 | 1.558 |
| 12 | 0 | 0 | 0 | 5.867 |
| 13 | 0 | 0 | 0 | 3.773 |
| 14 | -1 | 0 | -1 | 1.124 |
| 15 | 0 | 0 | 0 | 5.976 |
| 16 | 1 | 1 | 0 | 1.774 |
| 17 | -1 | -1 | 0 | 3.150 |

Note: A: Solid-to-Liquid Ratio (g/ml); B: Temperature (°C); C: Solvent Concentration (mol/L).

**Table S1-5** Experimental design and results of response surface methodology (RSM) for organic solvent-extracted selenium-containing proteins from *C. thesioides* fruits

| Run Number | Factor | | | Selenoprotein content  (mg/g) |
| --- | --- | --- | --- | --- |
|  | A | B | C |  |
| 1 | 0 | 1 | -1 | 2.293 |
| 2 | 1 | 0 | 1 | 2.496 |
| 3 | 0 | -1 | -1 | 1.711 |
| 4 | 0 | 0 | 0 | 3.159 |
| 5 | 0 | 0 | 0 | 3.186 |
| 6 | -1 | -1 | 0 | 1.843 |
| 7 | 1 | -1 | 0 | 2.794 |
| 8 | -1 | 0 | -1 | 1.707 |
| 9 | 0 | 0 | 0 | 3.244 |
| 10 | 0 | 0 | 0 | 3.222 |
| 11 | 0 | 0 | -1 | 1.889 |
| 12 | 0 | 1 | 1 | 2.933 |
| 13 | 0 | 1 | 0 | 2.963 |
| 14 | 0 | 0 | 0 | 2.942 |
| 15 | 0 | -1 | 1 | 2.578 |
| 16 | -1 | 1 | 0 | 2.505 |
| 17 | -1 | 0 | 1 | 2.424 |

Note: A: Solid-to-Liquid Ratio (g/ml); B: Temperature (°C); C: Solvent Concentration (%).
