# Supplementary material for: Optimization of the extraction process and in vitro antioxidant capacity analysis of selenium-containing proteins from Cynanchum thesioides
Source: PeerJ. 2026 Apr 15;14:e20998. doi: 10.7717/peerj.20998 (PMC13091576; doi:10.7717/peerj.20998)
Supplement: Supplemental Information 20 [file peerj-14-20998-s020.docx]

**Table S4** Summary statistics of the model

| Extraction Method | pH after Mixing | R² | P | Adjusted R² | Predicted R² | PRESS |
| --- | --- | --- | --- | --- | --- | --- |
| Water extraction | 6.7±0.3 | 0.993 | <0.0001 | 0.983 | 0.954 | 0.21 |
| Alkaline extraction | 12.2±0.2 | 0.906 | 0.0074 | 0.784 | 0.188 | 5.31 |
| Acid extraction | 1.7±0.1 | 0.907 | 0.0071 | 0.787 | 0.243 | 4.38 |
| Salt extraction | 6.9±0.1 | 0.841 | 0.038 | 0.636 | 0.376 | 2.94 |
| Organic solvent extraction | -- | 0.950 | 0.0009 | 0.887 | 0.389 | 2.85 |
