# Supplementary material for: Optimization of the extraction process and in vitro antioxidant capacity analysis of selenium-containing proteins from Cynanchum thesioides
Source: PeerJ. 2026 Apr 15;14:e20998. doi: 10.7717/peerj.20998 (PMC13091576; doi:10.7717/peerj.20998)
Supplement: Supplemental Information 21 [file peerj-14-20998-s021.docx]

**Table S5** Confidence Intervals and Model Goodness-of-fit for the Determination of Various Indicators

|  | η² | R² | Lower limit | Upper limit | IC50 (mg/L) |
| --- | --- | --- | --- | --- | --- |
| Vitamin C | 0.985 | 0.9996 | 105.1978 | 118.8394 |  |
| Soluble Sugars | 0.978 | 0.9949 | 2222.4596 | 2800.6017 |  |
| Crude Fiber | 0.88 | 0.9994 | 1.8713 | 1.9095 |  |
| Selenium Content | 0.985 | 0.9991 | 0.7381 | 0.8975 |  |
| Soluble Protein | 0.996 | 0.9992 | 5.1015 | 6.3295 |  |
| Crude Fat | 0.999 | 0.9993 | 0.0342 | 0.0533 |  |
| DPPH | 0.945 | 0.9991 | 51.6162 | 54.9604 | 0.98~4.21 |
| O₂·⁻ | 0.949 | 0.9612 | 43.4483 | 46.2956 | 1.18~5.13 |
| ·OH | 0.911 | 0.9759 | 53.7228 | 56.9650 | 1.98~6.98 |
| FRAP | 0.902 | 0.9928 | 0.6829 | 0.8538 |  |
