# Supplementary material for: Optimization of the extraction process and in vitro antioxidant capacity analysis of selenium-containing proteins from Cynanchum thesioides
Source: PeerJ. 2026 Apr 15;14:e20998. doi: 10.7717/peerj.20998 (PMC13091576; doi:10.7717/peerj.20998)
Supplement: Supplemental Information 22 — Note: *The correlation is significant at the 0.05 level (two-tailed). ** The correlation is significant at the 0.01 level (two-tailed). [file peerj-14-20998-s022.docx]

**Table S6** Correlation analysis between selenium fertilizer concentration and antioxidant activity indicators

|  | DPPH | O₂·⁻ | ·OH | FRAP |
| --- | --- | --- | --- | --- |
| Selenium fertilizer concentration | .982^**^ | .918^**^ | .949^**^ | .992^**^ |

Note: *The correlation is significant at the 0.05 level (two-tailed). ** The correlation is significant at the 0.01 level (two-tailed).
