# Supplementary material for: Optimization of the extraction process and in vitro antioxidant capacity analysis of selenium-containing proteins from Cynanchum thesioides
Source: PeerJ. 2026 Apr 15;14:e20998. doi: 10.7717/peerj.20998 (PMC13091576; doi:10.7717/peerj.20998)
Supplement: Supplemental Information 23 — Han (2023). Optimization of selenium protein extraction process from Cynanchum thesioides and analysis of its in vitro antioxidant activity (Doctoral Thesis). Inner Mongolia Agricultural University, Hohhot. [file peerj-14-20998-s023.pdf]

分类号 S609.9　　　　　　学校代码 10129
U D C 35.140　　　　　　学　　号 2021212260046

内蒙古農業大學

硕士学位论文

地梢瓜硒蛋白提取工艺优化及体外抗氧化能力分析

Optimization of extraction process of selenoprotein and analysis of antioxidant capacity in vitro of *Cynanchun thesioides* (Freyn).K.Schum

申 请 人：韩 旭

学生类别：全日制专业学位硕士

学位类别：农业硕士

领　　域：农艺与种业

指导教师：杨忠仁 教授

张凤兰 副教授

论文提交日期：二〇二三年六月

中国知网 https://www.cnki.net

摘　要

硒(Selenium,Se)是人体必需的微量元素，主要以硒蛋白的形式存在于植物体内，随食物链被人体吸收，在人体内参与重要的代谢反应。地梢瓜（*Cynanchum thesioides* (Freyn) K. Schum）又名沙奶奶、奶瓜等，是一种集饲用、药用、食用及工业原料于一体的植物，其中雀瓢为地梢瓜变种。相关研究表明地梢瓜富硒能力较强，其硒含量远高于一些常见的蔬菜。因此，本实验以地梢瓜、雀瓢果实为实验材料，测定其不同果实类型的水分、维生素 C、粗纤维、粗脂肪、可溶性总糖、可溶性蛋白以及硒含量；并采用水提法、盐提法、酸提法、碱提法和有机溶剂提取法提取果实硒蛋白并通过响应面法优化其提取工艺；最后采用不同浓度硒肥对地梢瓜植株进行施硒处理，探究其果实硒含量变化及其体外抗氧化活性，以期为地梢瓜果实中硒蛋白提取、利用提供技术支撑。具体结果如下：

1.水分含量地梢瓜小果最高，为 90.01 %；维生素 C、可溶性总糖、可溶性蛋白含量雀瓢小果最高，分别 135.10 mg/100 g、3.55 %、8.04 mg/g；粗纤维、粗脂肪、硒含量雀瓢大果最高，分别为 1.948 %、7.3 %、1.06 mg/kg。

2.水提法最佳提取工艺为：料液比 1：15.87、提取温度 28.64 ℃、时间 2.29 h，理论硒蛋白含量为2.741 mg/g；碱提法最佳提取工艺为：料液比 1：22.7、提取温度 44.62 ℃、溶剂浓度 0.243 mol/L，理论硒蛋白含量为 5.925 mg/g；酸提法最佳提取工艺为：料液比 1：28.6、提取温度 51.603 ℃、溶剂浓度 0.192 mol/L，理论硒蛋白含量为 2.762 mg/g；盐提法最佳提取工艺为：料液比 1：21.74、提取温度 35.99 ℃、溶剂浓度 0.141 mg/L，理论硒蛋白含量为 4.731 mg/g；有机溶剂提取法最佳提取工艺为：料液比 1:23.3、提取温度 52.485 ℃、溶剂浓度 81.37 %，理论硒蛋白含量为 3.271 mg/g，5 种提取方法中，碱提法提取的硒蛋白含量最高。

3.在硒肥施用浓度 2 mg/L 样品稀释浓度为 5 mg/mL 时，地梢瓜果实、提取液中硒含量最高分别为 1.92 mg/kg、0.58 mg/kg，此时地梢瓜果实硒蛋白清除 DPPH 自由基、羟自由基、超氧阴离子以及还原能力明显强于其他浓度处理。

关键词：地梢瓜；硒蛋白；体外抗氧化活性；响应面法；提取工艺

中国知网　https://www.cnki.net

Optimization of extraction process of selenoprotein and analysis of antioxidant capacity in vitro of *Cynanchun thesioides* (Freyn). K. Schum

Abstract

Selenium (Se) is an essential trace element for human body. It mainly exists in the form of selenium protein in plants. It is absorbed by human body along the food chain and takes part in important metabolic reactions in human body. *Cynanchum thesioides* (Freyn) K. Schum, also known as sand grandma, milk melon, etc., is a kind of plant integrating feeding, medicinal, edible and industrial raw materials. Among them, *Cynanchum thesioides* (Freyn)K. *Schum. var. australe* (Maxim.) Tsiang et P. T. L. Is variant of *C. thesioides*. Relevant studies have shown that *C. thesioides* has strong selenium enrichment ability, and its selenium content is much higher than that of some common vegetables. Therefore, in this study, *C. thesioides* and *C. australe* fruits were used as experimental materials to determine the contents of water, vitamin C, crude fiber, crude fat, soluble total sugar, soluble protein and selenium in different fruit types. The selenium protein was extracted by water extraction, salt extraction, acid extraction, alkali extraction and organic solvent extraction, and the extraction technology was optimized by response surface method. Finally, plants of *C. thesioides* were treated with selenium fertilizer of different concentrations to explore the changes of selenium content and in vitro antioxidant activities of their fruits, in order to provide technical support for the extraction and utilization of selenium protein from *C. thesioides* fruits. The specific results are as follows:

1. The water content of *C. thesioides* was the highest, 90.01 %; *C. australe* had the highest content of vitamin C, soluble total sugar and soluble protein, which were 135.10 mg/100g, 3.55 % and 8.04 mg/g, respectively. The contents of crude fiber, crude fat and selenium *C. australe* were the highest, which were 1.948 %, 7.3 % and 1.06 mg/kg, respectively.

2. The optimum extraction process of water extraction was as follows : the ratio of material to liquid was 1:15.87, the extraction temperature was 28.64 °C, the extraction time was 2.29 h, and the theoretical selenoprotein content was 2.741 mg/g ; the optimum extraction process of alkali extraction was as follows : solid-liquid ratio 1:22.7, extraction temperature 44.62 °C, solvent concentration 0.243 mol/L, and theoretical selenoprotein content 5.925 mg/g. The optimum extraction process of acid extraction was as follows : solid-liquid ratio 1:28.6, extraction temperature 51.603 °C, solvent concentration 0.192mol/L, theoretical selenoprotein content 2.762 mg/g. The optimum extraction process of salt extraction was as follows : solid-liquid ratio 1:21.74, extraction temperature 35.99 °C, solvent concentration 0.141 mg/L, theoretical selenoprotein content 4.731 mg/g ; the optimum extraction process of organic solvent extraction method was as follows : the ratio of material to liquid was 1:23.3, the extraction temperature was 52.485 °C, the solvent concentration was 81.37 %, and the theoretical selenoprotein content was 3.271 mg/g. Among the five extraction methods, the selenoprotein content extracted by alkali extraction

中国知网 https://www.cnki.net

was the highest.

3. The highest selenium content in *C. thesioides* fruit and extract was 1.92 mg/kg and 0.58 mg/kg, respectively, when the diluted concentration of 2 mg/L selenium fertilizer sample was 5 mg/mL. The scavenging ability of selenium protein in *C. thesioides* fruit on DPPH free radical, hydroxyl free radical, superoxide anion and reducing ability was significantly stronger than that in other concentration treatments.

Key Words: *Cynanchun thesioides (Freyn). K. Schum ; Selenoprotein ; Antioxidant activity in vitro ; Response surface method ; Extraction process*

中国知网 https://www.cnki.net

目　录

1 引言......1
1.1 硒......1
1.1.1 植物硒的存在形式......1
1.1.2 硒蛋白的提取方法......1
1.1.3 硒蛋白的抗氧化作用......2
1.2 地梢瓜......3
1.2.1 地梢瓜的化学成分......3
1.2.2 地梢瓜的营养价值......3
1.3 研究的目的意义......4
2 地梢瓜果实营养成分分析......5
2.1 材料与方法......5
2.2 实验试剂......5
2.3 实验仪器......5
2.4 测定指标及其方法......5
2.5 数据分析......5
2.6 结果与分析......5
2.6.1 地梢瓜、雀瓢不同类型果实水分含量的分析......5
2.6.2 地梢瓜、雀瓢不同类型果实维生素 C 含量的分析......6
2.6.3 地梢瓜、雀瓢不同类型果实可溶性总糖含量的分析......7
2.6.4 地梢瓜、雀瓢不同类型果实粗纤维含量的分析......7
2.6.5 地梢瓜、雀瓢不同类型果实粗脂肪含量的分析......8
2.6.6 地梢瓜、雀瓢不同类型果实可溶性蛋白含量的分析......8
2.6.7 地梢瓜、雀瓢不同类型果实硒含量的分析......9
2.7 讨论......10
3 不同提取方法提取地梢瓜果实硒蛋白工艺优化......11
3.1 实验材料与方法......11
3.1.1 实验试剂......11
3.1.2 实验仪器......11
3.1.3 实验方法......11
3.2 水提法提取地梢瓜果实硒蛋白......11
3.2.1 提取时间对地梢瓜硒蛋白提取的影响......11
3.2.2 提取温度对地梢瓜硒蛋白提取的影响......11
3.2.3 料液比对地梢瓜硒蛋白提取的影响......12
3.2.4 响应面法优化地梢瓜硒蛋白提取工艺......12

中国知网 https://www.cnki.net

3.3 碱提法提取地梢瓜果实硒蛋白 12
3.3.1 提取时间的单因素安排..12
3.3.2 提取温度的单因素安排..12
3.3.3 料液比的单因素安排..13
3.3.4 氢氧化钠浓度的单因素安排..13
3.3.5 响应面法优化提取工艺..13
3.4 酸提法提取地梢瓜果实硒蛋白 13
3.4.1 提取时间的单因素安排..13
3.4.2 提取温度的单因素安排..14
3.4.3 料液比的单因素安排..14
3.4.4 盐酸浓度的单因素安排..14
3.4.5 响应面法优化提取工艺..14
3.5 盐提法提取地梢瓜果实硒蛋白 14
3.5.1 提取时间的单因素安排..15
3.5.2 提取温度的单因素安排..15
3.5.3 料液比的单因素安排..15
3.5.4 氯化钠浓度的单因素安排..15
3.5.5 响应面法优化提取工艺..15
3.6 有机溶剂(乙醇)提取法提取地梢瓜果实硒蛋白..16
3.6.1 提取时间的单因素安排..16
3.6.2 提取温度的单因素安排..16
3.6.3 料液比的单因素安排..16
3.6.4 乙醇浓度的单因素安排..16
3.6.5 响应面法优化提取工艺..17
3.7 数据处理方法..17
3.8 结果与分析..17
3.8.1 水提法对地梢瓜果实硒蛋白含量的影响 17
3.8.2 碱提法对地梢瓜果实硒蛋白含量的影响 22
3.8.3 酸提法对地梢瓜果实硒蛋白含量的影响 28
3.8.4 盐提法对地梢瓜果实硒蛋白含量的影响 33
3.8.5 有机溶剂提取法对地梢瓜果实硒蛋白含量的影响 39
3.9 讨论..45
4 硒肥对地梢瓜果实硒含量及其体外抗氧化活性的影响 48
4.1 材料与方法..48
4.1.1 实验材料..48
4.1.2 实验试剂..48
4.1.3 实验仪器..48
4.1.4 实验方法..48

中国知网 https://www.cnki.net

4.2 硒肥处理果实及提取液中硒含量的测定 48
4.3 硒肥对地梢瓜果实硒蛋白体外抗氧化活性的影响 48
4.4 数据处理方法......49
4.5 结果与分析......49
4.5.1 不同浓度硒肥对果实及提取液中硒含量的影响 49
4.5.2 清除 DPPH 效果分析 49
4.5.3 还原能力分析......50
4.5.4 清除羟自由基能力分析......51
4.5.5 清除超氧阴离子能力分析......51
4.6 讨论......52
5 结论与展望......54
5.1 结论......54
5.2 展望......54
参考文献......55

中国知网 https://www.cnki.net

插图和附表清单

1. 图 1　地梢瓜和雀瓢果实含水量分析......6
2. 图 2　地梢瓜和雀瓢果实维生素 C 含量分析......6
3. 图 3　地梢瓜和雀瓢果实可溶性总糖含量分析......7
4. 图 4　地梢瓜和雀瓢果实粗纤维含量分析......8
5. 图 5　地梢瓜和雀瓢果实粗脂肪含量分析......8
6. 图 6　地梢瓜和雀瓢果实可溶性蛋白含量分析......9
7. 图 7　地梢瓜和雀瓢果实硒含量分析......9
8. 图 8　不同提取时间对地梢瓜果实可溶性硒蛋白含量的影响......17
9. 图 9　不同提取温度对地梢瓜果实可溶性硒蛋白含量的影响......18
10. 图 10　不同料液比对地梢瓜果实可溶性硒蛋白含量的影响......18
11. 图 11　各两因素交互作用对地梢瓜果实可溶性硒蛋白含量影响的三维响应曲线图和等高线图......21
12. 图 12　不同提取时间对地梢瓜果实可溶性硒蛋白含量的影响......22
13. 图 13　不同提取温度对地梢瓜果实可溶性硒蛋白含量的影响......23
14. 图 14　不同料液比对地梢瓜果实可溶性硒蛋白含量的影响......23
15. 图 15　不同溶剂浓度对地梢瓜果实可溶性硒蛋白含量的影响......24
16. 图 16　各两因素交互作用对地梢瓜果实可溶性硒蛋白含量影响的三维响应曲线图和等高线图......27
17. 图 17　不同提取时间对地梢瓜果实可溶性硒蛋白含量的影响......28
18. 图 18　不同提取温度对地梢瓜果实可溶性硒蛋白含量的影响......29
19. 图 19　不同料液比对地梢瓜果实可溶性硒蛋白含量的影响......29
20. 图 20　不同溶剂浓度对地梢瓜果实可溶性硒蛋白含量的影响......30
21. 图 21　各两因素交互作用对地梢瓜果实可溶性硒蛋白含量影响的三维响应曲线图和等高线图......32
22. 图 22　不同提取时间对地梢瓜果实可溶性硒蛋白含量的影响......33
23. 图 23　不同提取温度对地梢瓜果实可溶性硒蛋白含量的影响......34
24. 图 24　不同料液比对地梢瓜果实可溶性硒蛋白含量的影响......34
25. 图 25　不同溶剂浓度对地梢瓜果实可溶性硒蛋白含量的影响......35
26. 图 26　各两因素交互作用对地梢瓜果实可溶性硒蛋白含量影响的三维响应曲线图和等高线图......38
27. 图 27　不同提取时间对地梢瓜果实可溶性硒蛋白含量的影响......39
28. 图 28　不同提取温度对地梢瓜果实可溶性硒蛋白含量的影响......40
29. 图 29　不同料液比对地梢瓜果实可溶性硒蛋白含量的影响......40
30. 图 30　不同溶剂浓度对地梢瓜果实可溶性硒蛋白含量的影响......41
31. 图 31　各两因素交互作用对地梢瓜果实可溶性硒蛋白含量影响的三维响应曲线图和等高线图......44
32. 图 32　不同浓度硒肥处理果实及提取液中硒含量的影响......49
33. 图 33　地梢瓜硒蛋白提取液体外清除 DPPH 自由基能力测定结果......50
34. 图 34　地梢瓜硒蛋白提取液还原能力测定结果......50

中国知网　https://www.cnki.net

35. 图 35　地梢瓜硒蛋白提取液体外清除羟自由基能力测定结果......51
36. 图 36　地梢瓜硒蛋白提取液体外清除超氧阴离子能力测定结果......52

37. 表 1　水提法提取地梢瓜果实可溶性硒蛋白因素......11
38. 表 2　响应面设计的因素和水平......12
39. 表 3　碱提法提取地梢瓜果实可溶性硒蛋白因素考察水平......12
40. 表 4　响应面设计的因素和水平......13
41. 表 5　酸提法提取地梢瓜果实可溶性硒蛋白因素考察水平......13
42. 表 6　响应面设计的因素和水平......14
43. 表 7　盐提法提取地梢瓜果实可溶性硒蛋白因素考察水平......15
44. 表 8　响应面设计的因素和水平......16
45. 表 9　有机溶剂提取法提取地梢瓜果实可溶性硒蛋白因素考察水平......16
46. 表 10　响应面设计的因素和水平......17
47. 表 11　地梢瓜果实可溶性硒蛋白响应面分析方案与结果......19
48. 表 12　回归模型方程的方差分析......20
49. 表 13　验证实验结果......22
50. 表 14　地梢瓜果实可溶性硒蛋白响应面分析方案与结果......25
51. 表 15　回归模型方程的方差分析......25
52. 表 16　验证实验结果......28
53. 表 17　地梢瓜果实可溶性硒蛋白响应面分析方案与结果......30
54. 表 18　回归模型方程的方差分析......31
55. 表 19　验证实验结果......33
56. 表 20　地梢瓜果实可溶性硒蛋白响应面分析方案与结果......36
57. 表 21　回归模型方程的方差分析......36
58. 表 22　验证实验结果......39
59. 表 23　地梢瓜果实可溶性硒蛋白响应面分析方案与结果......42
60. 表 24　回归模型方程的方差分析......42
61. 表 25　验证实验结果......45

中国知网　https://www.cnki.net

1 引言

1.1 硒

硒（Selenium， Se）是动物体内所必需的营养元素和植物的有益营养元素，同时也是人体必需的微量元素[1]，1973 年，世界卫生组织（World Health Organization）正式宣布硒是人体必需的微量元素之一。硒元素自 1817 年被贝采利乌斯发现以来一直是国内外学者研究的热点话题。相关研究表明，硒对动物和人类具有重要的生理作用，其在抗癌防癌、人体免疫力调节、预防心脑血管疾病、抗衰老等诸多方面均具有积极作用，同时硒还被广泛的应用于工业生产上，硒对重金属、辐射、微波有解除毒害的效应[2-6]。硒可作为动物饲料添加剂，也可以在肥料中添加微量硒，来提高农副产品硒含量，其对植物的生理作用主要表现在提高植物产量，改善果实品质，还可以植物体内其他元素发生相互作用，来提高植物对环境、生理和病理逆境的抵抗能力等[7-10]。全世界有 40 多个国家和地区属于缺硒地区，我国是一个缺硒大国，据《中华人民共和国地方疾病与环境因素图集》揭示，从东北三省至云贵高原，占我国国土面积的 72 %地区存在一条低硒地带，华北、东北、西北、华南、华东、珠江三角洲、长江三角洲等大中城市都属于缺硒地区。我国二十二个省市的部分地区，约七亿人生活在低硒地区。硒和其他元素一样，无法由机体合成，所以要保证人体对硒的需求，就需要通过每天摄入一定量的硒。硒摄入不足，会引发克山病、大骨节病、癌症、糖尿病、心脑血管、高血压综合症等疾病[11-13]。虽然硒在人体中具有不可替代的作用，但也要重视硒过量引起的硒中毒，目前常见的补硒方式主要有通过无机亚硒酸钠补硒和通过食品摄入两种，无机亚硒酸钠是一种剧毒物质，毒性大，且动物对无机硒吸收利用率不高，而人的安全膳食硒摄入量为 50-500 μg/d，与中毒剂量(750 μg/d)相差范围小。故以亚硒酸钠作为动物及人体补硒的直接硒源，其风险大；生物大分子结合态有机硒安全、生物活性好、吸收率较高，所以有机硒补剂产品的开发和研究受到了广泛关注。研究表明，通过食物链转化，从天然食品中摄取硒是一种安全的补硒途径[14-16]。

1.1.1 植物硒的存在形式

硒被植物体吸收后，形成非常复杂的化学形态，根据组成结构的差别分为无机硒和有机硒两大类[17]。无机硒较少，包括硒酸、亚硒酸和其他一些无机形态(如 Se^{2-}和 HSe^{-})，且主要以 Se(IV)形式存在[18]。硒在植物中存在的主要形态为有机硒。小分子的有机硒形态主要是硒代氨基酸及其衍生物，主要有硒代半胱氨酸、硒代胱氨酸、硒代蛋氨酸、二甲基二硒醚、硒代高胱氨酸、硒代半胱氨酸和亚硒酸等[19-21]。植物中以大分子形态存在的硒主要有硒蛋白、硒核酸、硒多糖等，其中硒蛋白是大分子硒的主要存在形态。研究表明，植物体内的硒大部分为有机硒，占总量的 80 %以上，其中又以蛋白硒为主。硒蛋白是指硒以硒代半胱氨酸（Selenocysteine，Sec）形式通过氨基酸脱水缩合作用结合到肽链上所形成的并表现出硒生理活性的一类蛋白质[22]。

1.1.2 硒蛋白的提取方法

硒蛋白的提取与分离，是深入研究其生物与化学形态的前提，因其本身就是蛋白质的一类，所以常见的蛋白质分离提取方法都可以用于植物含硒蛋白的分离。在国内外关于蛋白质提取的研究中常用的方法主要有溶剂提取法、柱分离技术、生物学方法等。溶剂提取法具有提取方法简单、成本低等优点因此常被应用于用于实验室。溶剂提取法又因提取时选用的提取溶剂类型被分为水提法、碱提法、盐提法、酸提法、有

中国知网 https://www.cnki.net

机溶剂提取法等。

水提法：则拉莱·司玛依等[23]通过水提法、乙醇提取法、醋酸提取法、碱溶酸沉法、磷酸盐缓冲液提取法分别提取乌拉尔甘草、胀果甘草及光果甘草种子的蛋白，结果表明水提法的提取率最高，3 种蛋白含量分别为 36.54 mg/mL、30.80 mg/mL、30.12 mg/mL，显著高于其他几种提取方法的蛋白质含量。

碱提法：碱提法是溶剂提取法提取蛋白的最常用溶剂。冯谈林等[24]选取 NaOH 浓度、超声波时间、料液比、超声波功率做单因素实验探究不同因素对苦菜硒蛋白提取的影响，结果表明在 NaOH 浓度 0.2 mol/L，料液比 1：25 g/mL，超声波功率 78 W，超声时间 14 min 的条件下提取效果最佳，苦菜硒蛋白提取量为 2.595 mg/g。

盐提法:秦之皓[25]以富硒平菇为原料使用超声波辅助盐提法提取富硒平菇蛋白，对提取温度、提取时间以及盐浓度进行单因素实验，最终确定超声波辅助盐提法提取富硒平菇蛋白的最适工艺条件为：提取时间 120 min，盐浓度 0.5 ml/L，液固比 30 mL/g，提取温度 50 ℃，在此条件下蛋白得率为 39.6 %。对比纤维素酶处理、半纤维素酶处理、超声波辅助水提法、碱提法、超声波辅助碱提法和盐提法，确定超声波辅助盐提法的蛋白提取率高。

酸提法：马洪鑫[26]通过比较不同方法提取藜麦蛋白并通过扫描电镜观察蛋白质的超显微结构，结果表明碱溶酸沉法提取效果最好，酸提法制备的蛋白质外观最好。

有机溶剂提取法：杨柳[27]等人采用正交实验探究有机溶剂法提取玉米蛋白的最佳提取工艺，选取乙醇质量分数、温度、pH 值为探究因素，实验结果表明 90 %的乙醇溶液，在 55 ℃，pH6.0 的条件下，可将玉米醇溶蛋白从玉米蛋白粉中提取出来，在此条件下玉米醇溶蛋白的得率是 78.94 %。

1.1.3 硒蛋白的抗氧化作用

硒是动物和人体中的谷胱甘肽过氧化物酶和硒-P 蛋白的重要组成部分，在体内起着平衡氧化还原的作用; 氧化应激是指体内活性氧自由基(ROS)等高活性分子产生过多，超出自身过氧化物清除能力，导致机体氧化和抗氧化系统失衡[28]。研究证明其具有提高动物免疫力的作用。硒以硒蛋白的形式参与机体抗氧化和免疫调节机制，硒蛋白 K(SELENOK）和硒蛋白 S(SELENOS）是与炎症和免疫相关的 2 种硒蛋白，并且在导致内质网应激的条件下发挥保护细胞的作用[29]。硒蛋白中有五种 GPx:胞质 GPx(GPx_1)、胃肠特异性 GPx(GPx_2)、血浆 GPx(GPx_3)、磷脂氢过氧化物 GPx(GPx_4)和嗅觉上皮与胚胎组织特异性 GPx(GPx_6)，这些是人类抗氧化防御系统中特征明确的主要硒酶。$GPx_{1\text{-}3}$ 催化过氧化氢和有机氢过氧化物的还原，而 GPx_4 可以直接还原磷脂氢过氧化物和胆固醇氢过氧化物。陈春英等[30]观察了烟叶硒蛋白对四氯化碳肝损伤小鼠的保护作用，认为提前补充烟叶硒蛋白 7 天，可降低四氯化碳对肝脏的损伤，并呈现出剂量效应关系；对脂质过氧化物作用方面，硒蛋白明显优于亚硒酸钠和不含硒的蛋白组；烟叶硒蛋白对红细胞 γ 照射也具有良好的预防效果，对羟自由基有明显的清除作用。吴春凤等[31]研究结果表明硒能够通过增加硒蛋白的表达水平进而降低氧化应激水平，减少氧化应激损伤，同时增加细胞增殖活性，降低细胞凋亡水平，从而达到保护子痫前期胎盘滋养层细胞免受氧化应激损伤。铁梅等[32]采用紫外可见分光光度法研究富硒食用菌中硒蛋白和过氧化氢酶对羟自由基的清除作用，结果表明硒蛋白对羟自由基的清除率高于同质量浓度的蛋白质和亚硒酸钠，表明硒增强食用菌蛋白消除羟自由基的能力，且随着加入量的增加，硒蛋白、蛋白质和亚硒酸钠对羟自由基

中国知网 https://www.cnki.net

的清除率均增强；硒蛋白的加入促进过氧化氢酶对羟自由基的清除作用，两者在清除羟自由基方面存在着协同作用。

1.2 地梢瓜

地梢瓜（*Cynanchum thesioides* (Freyn) K. Schum）和雀瓢（*Cynanchum thesioides* (Freyn)K. *Schum.var.australe* (Maxim.) Tsiang et P.T.Li.）为萝藦科鹅绒藤属多年旱生直立半灌木，是一种集饲用、药用、食用及工业原料于一体的植物，其中雀瓢为地梢瓜变种。近年来，地梢瓜作为一种野生蔬菜受到越来越多的人所关注，分布在我国北方的大部分地区以及蒙古、朝鲜和俄罗斯等国。地梢瓜具有较强的抗旱性、抗病性和环境适应能力，可以生长在干旱山谷、路边和沙漠化的土地的条件下，也可在海拔 200-2000 m 的山坡上生长，是防风固沙和水土保持的先锋植物[33-35]。地梢瓜全草及果实均可入药，具有抗癌抗肿瘤、提高免疫力、益气、通乳等功效，外用可治瘊子、尖锐湿疣[36]，在中药资源研发利用具有重要价值。

1.2.1 地梢瓜的化学成分

植物的化学成分主要包括生物碱类、黄酮类、萜类、苯类衍生物、脂肪烷及脂肪酸类等。苑辉卿等[37]从地梢瓜全草中分离得到 14 个化合物，其中有两个为新分离鉴定的化合物。王玎等[38]利用硅胶、Sephadex LH-20 柱色谱及 HPLC 等方法对地梢瓜果实的化学成分进行分离纯化，从中新分离得到 3 个新成分，其中两个为在该属植物中首次分离得到。张晓艳等[39]对干旱胁迫下地梢瓜琥珀酸的合成代谢进行研究，结果表明琥珀酸在地梢瓜的根、茎、叶中均有分布，不同部位的琥珀酸含量存在差异，适度干旱胁迫能够促进地梢瓜琥珀酸的积累。喜杰等[40]以抗炎活性为基础，对地梢瓜进行地梢瓜苷的分离提纯探究其抗炎活性，结果表明地梢瓜苷可能是地梢瓜具有抗炎活性成分。

1.2.2 地梢瓜的营养价值

地梢瓜木质化程度低，质地柔软有多种次级代谢产物，是一种常见的食药两用特色植物资源。地梢瓜具有较高营养价值，其果实中有丰富的乳汁，含有较多营养成分和多种对人体有益的矿质元素，吃起来口感脆嫩香甜，散发淡淡的奶香味。且食用方法简单，鲜食、凉拌、清炒均可，在民间多被作为绿色野生蔬菜食用，深受农牧区居民的青睐[41-44]。丁梦军等[45]对 7 种野生植物的营养成分进行比较，结果表明地梢瓜中性洗涤纤维含量、酸性洗涤纤维含量、可溶性蛋白含量均最高。金世超[46]利用不同提取方法探究地梢瓜脂溶性成分的最佳工艺条件，结果表明在闪式提取法的最佳工艺下，地梢瓜地上部分干粉样品中脂溶性成分提取得率为 7.25 %±0.18 %，其植物体脂溶性提取物的成分主要为棕榈酸、亚麻酸、硬脂酸、油酸和烃类等。林敏等[47]采用火焰原子吸收光谱法对地梢瓜根、茎和叶消解液中的 9 种金属元素含量进行测定，结果表明地梢瓜不同部位各元素含量存在一定差异，各部位均含有丰富的 Ca、Mg、K、Na 和 Fe 元素。曹乌吉斯古楞[48]对内蒙古野生蔬菜资源综合评价，结果表明地梢瓜中含有较高的维生素和矿质元素。研究发现地梢瓜烘干全草样品中稀有元素硒的含量远高于食物成分表中 104 种蔬菜硒的平均含量，说明地梢瓜是一种天然富硒蔬菜[49]。

中国知网 https://www.cnki.net

1.3 研究的目的意义

随着生活质量的提高，人们越来越注重饮食健康。地梢瓜作为一种绿色的沙生蔬菜，具有药食两用的特性，其具有较高的营养价值和较丰富的矿质元素，口味独特，食用方法简单，且具有绿色安全无污染等特征。硒元素作为人体内一种必需的微量元素，在人体内发挥着至关重要的生理作用，硒摄入量不足会引发一系列疾病。2017 年，国家发布了卫生行业标准，规定成人硒的参考摄入量为 60 μg/d，据报道，我国成人硒平均摄入量为 44.4 μg/d，远远低于规定摄入量[50]。因此如何有效补硒，成为了现代研究的热点论题。因此，本文采用地梢瓜、雀瓢果实作为实验材料，测定其不同果实类型的水分、维生素 C、粗纤维、粗脂肪、可溶性总糖、可溶性蛋白以及硒含量；并采用不同的溶剂提取方法，提取果实硒蛋白，通过响应面法优化其提取工艺；最后采用不同浓度硒肥对地梢瓜植株进行施硒处理，探究其果实硒含量变化及其体外抗氧化活性，以期为地梢瓜果实中硒蛋白提取、利用提供技术支撑。

中国知网 https://www.cnki.net

2 地梢瓜果实营养成分分析

2.1 材料与方法

选择生长一致、良好的地梢瓜（*Cynanchum thesioides* （Freyn）K. Schum）和雀瓢（*Cynanchum thesioides* （Freyn）K. *Schum.var.australe* （Maxim.）Tsiang et P.T.Li.） 植株，随机摘取其不同大小果实，根据果实大小和宽度将其分为大果、中果和小果，长度指标以5 cm 为界限，宽度指标以1.5 cm 为界限，当果实同时满足长度大于5 cm， 宽度大于1.5 cm 时定义为大果，当果实满足长度、宽度中一项时定义为中果，两项均 不满足时定义为小果。

2.2 实验试剂

80 %乙醇、9.2 mol/L 以及 4.6 mol/L 高氯酸、0.1 mol/L 氢氧化钠、60 %硫酸、牛血清蛋白、考马斯亮蓝 G-250，90 %乙醇、85 %磷酸、石油醚（沸程 30-60 ℃）、抗坏 血酸、稀硫酸溶液、12.5 g/L 氢氧化钠、蒽酮、浓硝酸、硝酸镍。

2.3 实验仪器

FX101-0 型电热鼓风干燥箱、UH5300 型紫外分光光度计、CF16RN 型低温离心机、ME204型分析天平、ZX-S60型全不锈钢恒温水浴锅、索式脂肪提取器、干燥器、MuItiwave GO 型微波消解仪、09A24S 型赶酸仪、ZA3000 型原子吸收分光光度计。

2.4 测定指标及其方法

水分含量的测定用常压烘箱干燥法[51]，维生素 C 含量的测定采用紫外分光光度法[52]，可溶性总糖含量的测定采用蒽酮-硫酸法[51]，粗纤维含量的测定采用比色法[53]， 脂肪含量的测定采用索式抽提法[51]，可溶性蛋白含量的测定采用考马斯亮蓝染色法 [52]、硒含量测定采用石墨炉原子吸收法[54]。

2.5 数据分析

利用 Microsoft Office Excel 2010 软件处理和作图，IBM SPSS Statistics 22 软件进行差异性分析。

2.6 结果与分析

2.6.1 地梢瓜、雀瓢不同类型果实水分含量的分析

如图 1 所示，地梢瓜、雀瓢果实含水量均随果实增大逐渐降低，大果含水量较小 果含水量分别降低 18.39 %、19.44 %，且大中小地梢瓜、雀瓢果实间存在显著性差异 （P＜0.05），表明大果水分流失严重。雀瓢大中小果实含水量均低于地梢瓜，但差异 不显著（P＞0.05）。

中国知网 https://www.cnki.net

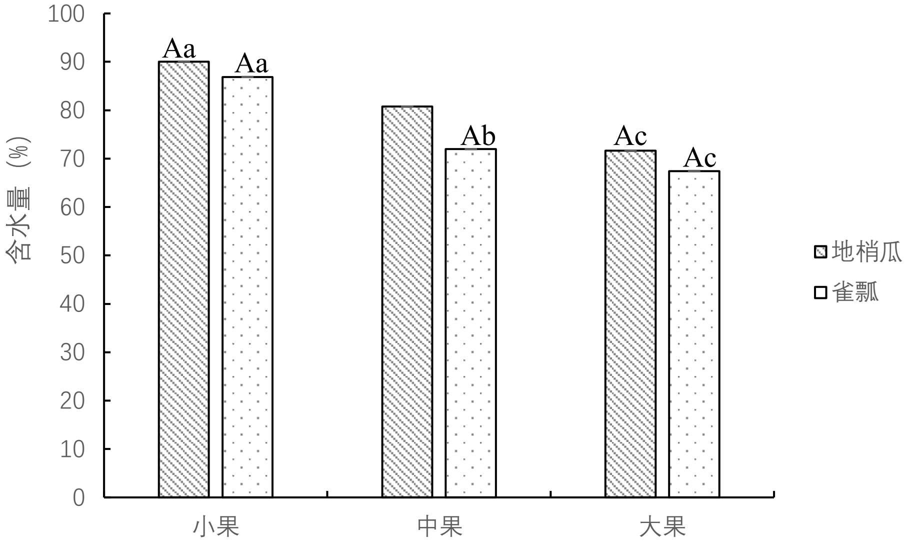

图 1 地稍瓜和雀瓢果实含水量分析

Fig.1 Analysis of fruit water content of C. thesioides

注：不同小写字母表示地稍瓜、雀瓢大中小果实间在 0.05 水平下的显著差异性；不同大写字母表示地稍瓜大中小果实与雀瓢大中小果实间在 0.05 水平下的显著性差异，下同。

2.6.2 地稍瓜、雀瓢不同类型果实维生素 C 含量的分析

如图 2 所示，地稍瓜、雀瓢果实维生素 C 含量均随果实增大先减少后增加，地稍瓜、雀瓢大中小果实维生素 C 含量均存在显著性差异（P＜0.05）。地稍瓜、雀瓢均是小果维生素 C 含量最高，分别达 107.09 mg/100 g、135.10 mg/100 g，中果维生素 C 含量最低，分别为 95.25 mg/100 g、111.03 mg/100 g。地稍瓜大中小果实维生素 C 含量均略低于雀瓢，且存在显著性差异（P＜0.05）。

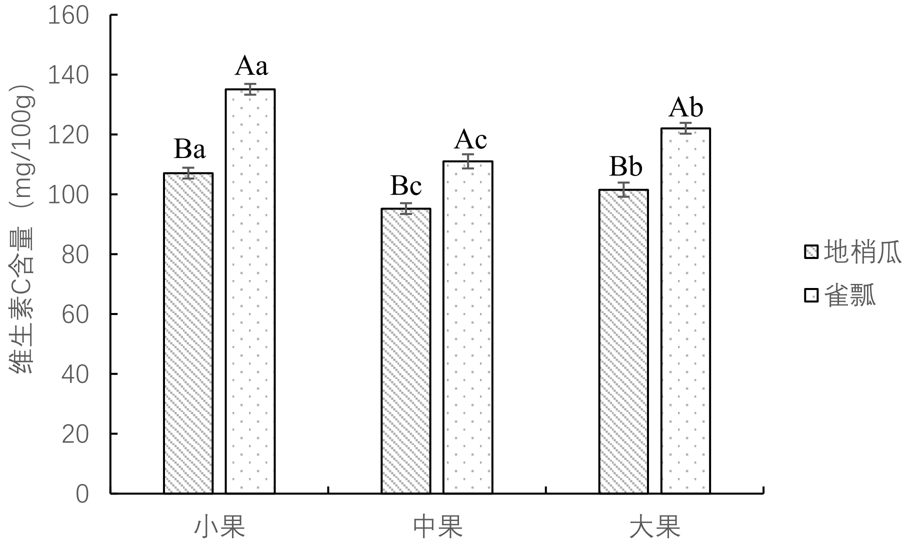

图 2 地稍瓜和雀瓢果实维生素 C 含量分析

Fig.2 Analysis of vitamin C content in Fruits of C. thesioides

中国知网 https://www.cnki.net

2.6.3 地梢瓜、雀瓢不同类型果实可溶性总糖含量的分析

如图 3 所示，地梢瓜、雀瓢果实可溶性糖含量随果实增大逐渐降低，地梢瓜、雀瓢大中小果实间均存在显著性差异（P＜0.05）。地梢瓜、雀瓢均是小果可溶性总糖含量最高，分别达 3.52 %、3.55 %，大果的可溶性总糖含量最低，分别为 3.41 %、3.45 %。地梢瓜大果、小果可溶性总糖含量显著低于雀瓢（P＜0.05）。

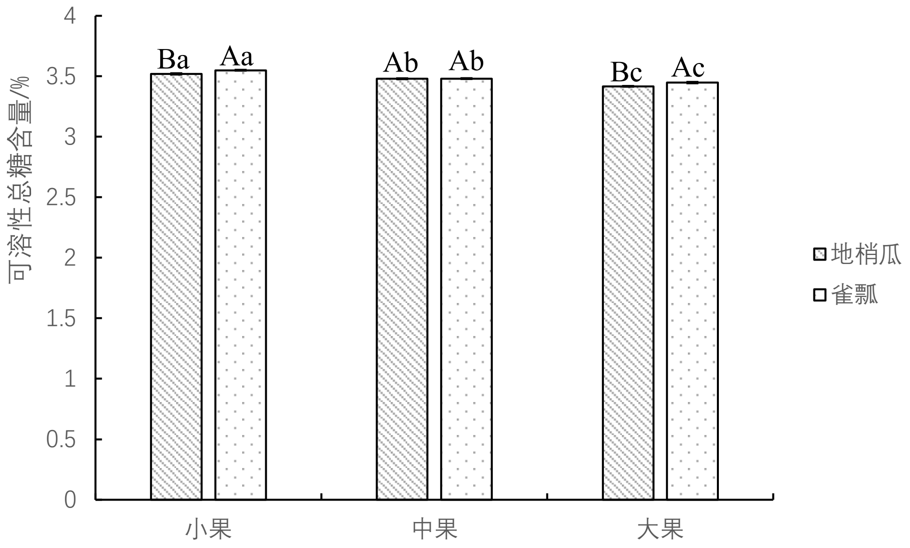

图 3 地梢瓜和雀瓢果实可溶性总糖含量分析

Fig.3 Analysis of total soluble sugar content in fruit of C. thesioides

2.6.4 地梢瓜、雀瓢不同类型果实粗纤维含量的分析

如图 4 所示，地梢瓜、雀瓢果实粗纤维含量随果实增大逐渐升高，地梢瓜中果、大果粗纤维含量显著性高于小果（P＜0.05）。雀瓢大果粗纤维含量显著高于小果、中果（P＜0.05）。地梢瓜、雀瓢大果粗纤维含量最高，分别达 1.903 %、1.948 %，地梢瓜、雀瓢均为小果粗纤维含量最低，分别为 1.834 %、1.869 %。雀瓢大果、小果粗纤维含量低于地梢瓜，雀瓢大果粗纤维含量显著性高于地梢瓜（P＜0.05）。

中国知网 https://www.cnki.net

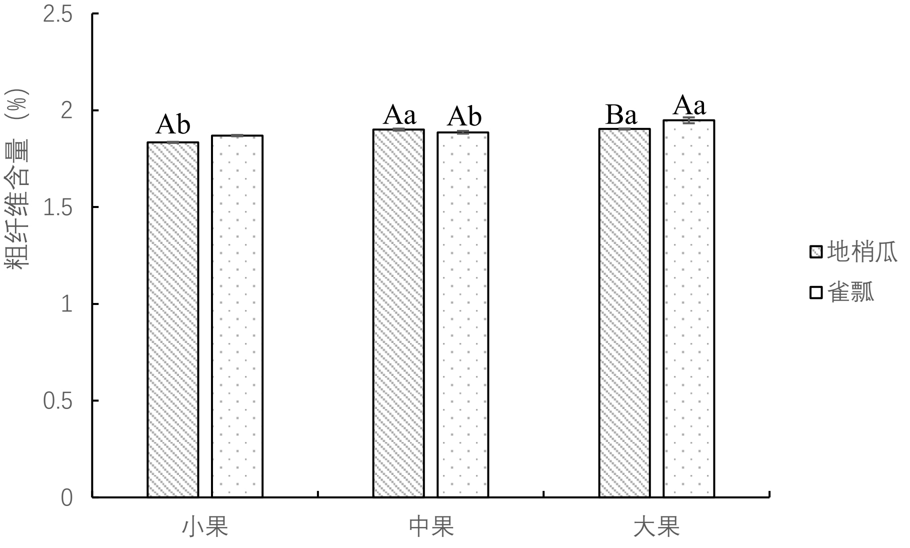

图 4　地梢瓜和雀瓢果实粗纤维含量分析

Fig.4　Analysis of crude fiber content in fruit of C. thesioides

2.6.5　地梢瓜、雀瓢不同类型果实粗脂肪含量的分析

如图 5 所示，地梢瓜果实粗脂肪含量随果实增大先增加后减少；雀瓢粗脂肪含量随果实增大逐渐增加；地梢瓜、雀瓢大中小果实间均存在显著性差异（P＜0.05）。地梢瓜中果、雀瓢大果粗脂肪含量最高，分别达 5.6 %、7.3 %，小果粗脂肪含量均最低，分别为 2.6 %、2.0 %。地梢瓜中果、小果粗纤维含量均高于雀瓢，地梢瓜、雀瓢大中小果实间均存在显著性差异（P＜0.05）。

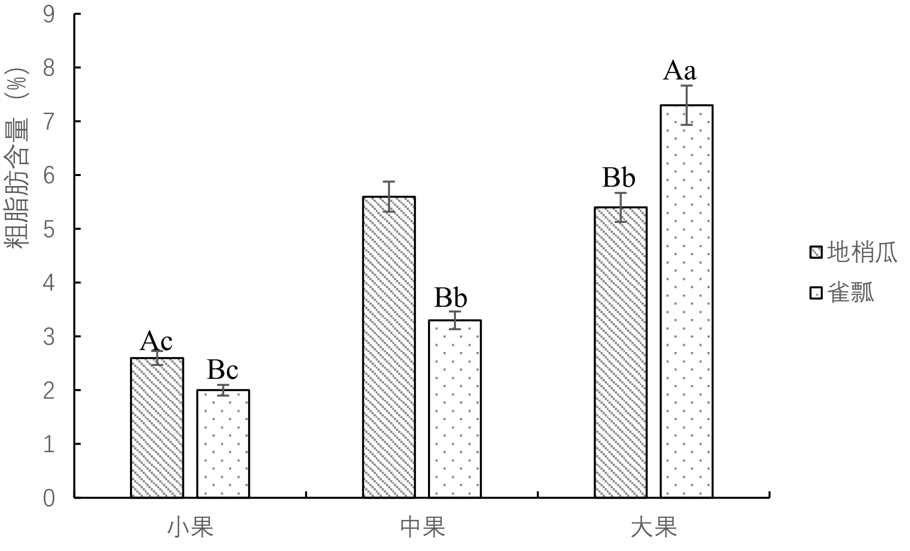

图 5　地梢瓜和雀瓢果实粗脂肪含量分析

Fig.5　Analysis of crude fat content in fruit of C. thesioides

2.6.6　地梢瓜、雀瓢不同类型果实可溶性蛋白含量的分析

如图 6 所示，地梢瓜果实可溶性蛋白含量随果实增大先减少后增加；雀瓢果实可

中国知网　https://www.cnki.net

溶性蛋白含量随果实增大逐渐降低。地梢瓜、雀瓢大中小果实间可溶性蛋白含量均存在显著性差异（P＜0.05）。地梢瓜、雀瓢均是小果可溶性蛋白含量最高，分别达 5.74 mg/g、8.04 mg/g，地梢瓜中果、雀瓢大果可溶性蛋白含量最低，分别为 4.29 mg/g、5.61 mg/g。地梢瓜大中小果实可溶性蛋白含量均显著低于雀瓢（P＜0.05）。

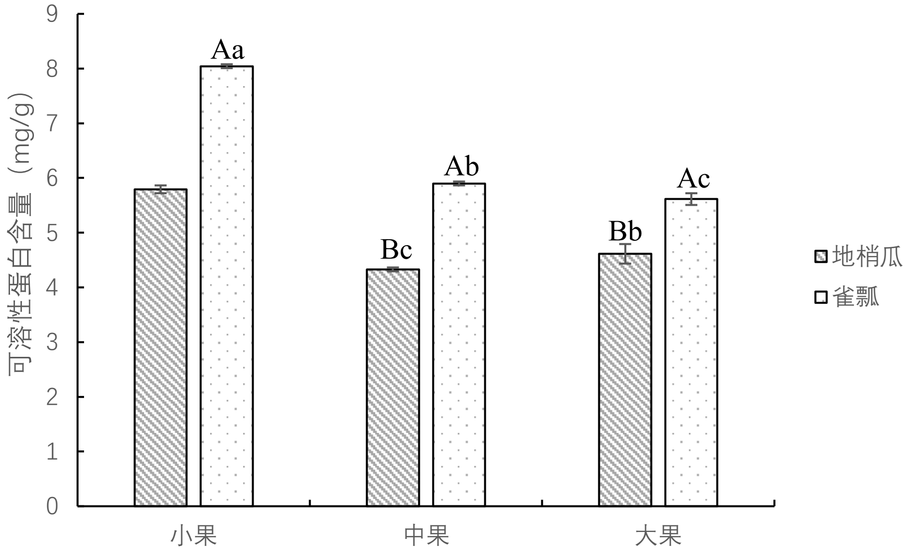

图 6　地梢瓜和雀瓢果实可溶性蛋白含量分析

Fig.6　Analysis of soluble protein content in fruit of C. thesioides

2.6.7　地梢瓜、雀瓢不同类型果实硒含量的分析

如图 7 所示，地梢瓜、雀瓢果实硒含量随果实增大均逐渐增加，地梢瓜、雀瓢大中小果实间硒含量存在显著性差异（P＜0.05）。地梢瓜、雀瓢均是大果硒含量最高，分别达 1.01 mg/kg、1.06 mg/kg，地梢瓜、雀瓢均是小果硒含量最低，分别为 0.66 mg/kg、0.69 mg/kg。地梢瓜大中小果实硒含量均显著低于雀瓢（P＜0.05）。

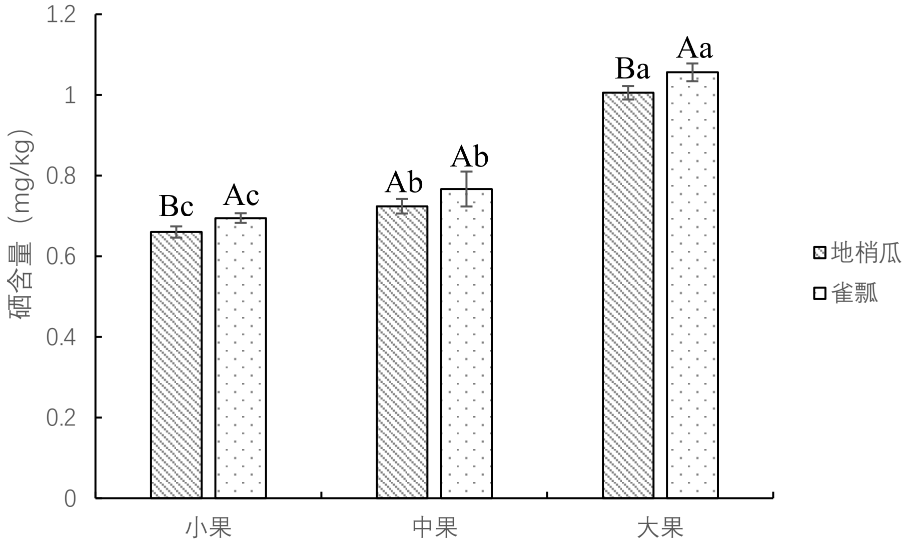

图 7　地梢瓜和雀瓢果实硒含量分析

Fig.7　Analysis of selenium content in fruit of C. thesioides

中国知网　https://www.cnki.net

2.7 讨论

植物的营养成分受分布地域、品种、生长发育阶段、土壤、海拔高度、季节、气候条件等多种因素的影响，一般时候植物的部位不同，营养成分也不同[55-56]。水分作为植物细胞内含物质的重要溶剂，其含量的多少影响着食物的口感，水分含量越高口感越脆、越好[57]。维生素 C 是人体所必需的营养物质之一，它对人体的健康状况具有重要的影响，其主要以还原型形态存在于新鲜的蔬菜水果中，维生素 C 在治疗受伤、灼伤、牙龈出血；帮助降低血液中的胆固醇；预防滤过性病毒和细菌的感染并增强免疫系统功能预防坏血病等方面疗效显著[58-59]。蛋白质是六大营养元素之一，植物性蛋白摄取不足会导致人的体力下降、精力不集中、抵抗力减弱等一系列不良症状[60]。可溶性糖广泛的存在于植物的果实之中，糖含量的多少是评价果实品质的重要指标之一。粗纤维作为膳食纤维的一部分，能够促进肠道蠕动，加快食物消化。植物组织中脂肪多为不饱和脂肪，对人体有较好的营养作用[61]。硒是人和动物体内所必须的微量元素，在机体内发挥重要的生理作用。植物营养物质的含量一定程度上决定了植物的食用价值。地梢瓜、雀瓢作为野生蔬菜，其营养物质含量丰富，水分含量地梢瓜小果最高，为 90.01 %；维生素 C、可溶性总糖、可溶性蛋白含量雀瓢小果最高，分别 135.10 mg/100 g、3.55 %、8.04 mg/g；粗纤维、粗脂肪、硒含量雀瓢大果最高，分别为 1.948 %、7.3 %、1.06 mg/kg。通过与茄子、马铃薯、菠菜等常规蔬菜[62]的营养物质进行比较，发现地梢瓜、雀瓢果实维生素 C、粗脂肪、粗纤维的含量均明显高于常规蔬菜；可溶性蛋白含量与常规蔬菜大致相等；可溶性总糖、水分含量略低于常规蔬菜，这与王春林等[57]对子午岭林区 19 种野菜的营养成分分析结果相似。地梢瓜、雀瓢果实硒含量随着果实增大呈增加的趋势，这可能是因为随着果实在增长时不断富硒所引起的，且其硒含量均高于常规的一些蔬菜[63]。宋东杰等[64]对方山 13 种野菜进行营养成分分析结果表明，泥胡菜粗蛋白、脂肪含量最高的分别为 3.08 %、0.97 %；碎米芥粗纤维含量最高为 5.87 %，均低于地梢瓜果实，由此可见地梢瓜部分营养成分不仅比一些常规蔬菜营养成分高，还比其他野菜要高，具有较高的营养价值。

中国知网 https://www.cnki.net

3 不同提取方法提取地梢瓜果实硒蛋白工艺优化

3.1 实验材料与方法

将采集自教学园区的地梢瓜果实洗净，60 ℃烘干，粉碎成粉，过 80 目筛，塑封袋密封，-18 ℃保存备用。

3.1.1 实验试剂

超纯水、氢氧化钠、盐酸、氯化钠、无水乙醇、考马斯亮蓝-G250、磷酸。

3.1.2 实验仪器

SHZ-B 水浴恒温振荡器、容量瓶、INFINITE200PRO 型酶标仪、移液枪、HC-2518R 型高速冷冻离心机、MuItiwave GO 型微波消解仪、09A24S 型赶酸仪、ZA3000 型原子吸收分光光度计。

3.1.3 实验方法

精准称取 0.1 g 地梢瓜果实粉末于 10 mL 离心管中，分别进行时间、温度、料液比、溶剂浓度的单因素实验。将离心管置于水域恒温震荡器中充分浸提后取出，冷冻离心后取 1 mL 上清液加入试管中，然后立即加入 5 mL G-250 考马斯亮蓝溶液，充分混匀后，取 0.25 mL 混合液于 96 孔酶标板中，使用酶标仪在 595 nm 波长下测定吸光值，根据标准曲线计算硒蛋白含量。

3.2 水提法提取地梢瓜果实硒蛋白

以超纯水为提取溶剂，选取提取时间、提取温度、料液比作为考察对象，采用单因素实验探究各因素对地梢瓜硒蛋白提取液中硒蛋白含量的影响，各因素的考察水平见表 1。

表 1　水提法提取地梢瓜果实可溶性硒蛋白因素考察水平

Table.1　Study on the factors of water extraction of soluble selenoprotein from CT fruit

提取时间（h）	提取温度（℃）	料液比（g/ml）
1.0	30	1:20
1.5	35	1:25
2.0	40	1:30
2.5	45	1:35
3.0	50	1:40

3.2.1 提取时间对地梢瓜硒蛋白提取的影响

精确称取 0.1 g 地梢瓜果实粉末于离心管中，设置提取条件为：温度 40 ℃，料液比 1：30，提取时间分别为 1.0 h、1.5 h、2.0 h、2.5 h、3.0 h 进行提取，设置 3 次重复。

3.2.2 提取温度对地梢瓜硒蛋白提取的影响

精确称取 0.1 g 地梢瓜果实粉末于离心管中，设置提取条件为：时间 2.0 h，料液比 1：30，提取温度分别为 30 ℃、35 ℃、40 ℃、45 ℃、50 ℃进行提取，设置 3 次

中国知网 https://www.cnki.net

重复。

3.2.3　料液比对地梢瓜硒蛋白提取的影响

精确称取 0.1 g 地梢瓜果实粉末于离心管中，设置提取条件为：时间 2.0 h，温度 40 ℃，料液比分别为 1：20、1：25、1：30、1：35、1：40 进行提取，设置 3 次重复。

3.2.4　响应面法优化地梢瓜硒蛋白提取工艺

以地梢瓜果实提取液硒蛋白含量为响应值，通过实验确定最佳水平范围并进行显著性分析，以提取时间、料液比、提取温度为因素，利用 Design-Expert 13 软件设计三因素三水平 BBD 响应面方法进行地梢瓜果实硒蛋白最佳提取工艺的选择，各因素设置水平如表 2 所示。

表 2　响应面设计的因素和水平

Table.2　Factors and levels of response surface design

水平	因素		
	A 时间（h）	B 料液比（%）	C 温度（℃）
-1	1.5	1：15	25
0	2.0	1：20	30
1	2.5	1：25	35

3.3　碱提法提取地梢瓜果实硒蛋白

选提取时间、提取温度、料液比、NaOH 浓度作为考察对象，采用单因素实验探究各因素对地梢瓜硒蛋白提取液中硒蛋白含量的影响，各因素的考察水平见表 3。

表 3　碱提法提取地梢瓜果实可溶性硒蛋白因素考察水平

Table.3　Study on the factors of extracting soluble selenoprotein from CT fruit by alkali extraction

提取时间（h）	提取温度（℃）	料液比（g/ml）	溶剂浓度（mol/L）
1.0	30	1:20	0.05
1.5	35	1:25	0.10
2.0	40	1:30	0.15
2.5	45	1:35	0.20
3.0	50	1:40	0.25

3.3.1　提取时间的单因素安排

精确称取 0.1 g 地梢瓜果实粉末于离心管中，设置提取条件为：温度 40 ℃，料液比 1：30，溶剂浓度 0.15 mol/L，提取时间分别为 1.0 h、1.5 h、2.0 h、2.5 h、3.0 h 进行提取，实验设置三次重复。

3.3.2　提取温度的单因素安排

精确称取 0.1 g 地梢瓜果实粉末于离心管中，设置提取条件为：时间 2.0 h，料液比 1：30，溶剂浓度 0.15 mol/L，提取温度分别为 30 ℃、35 ℃、40 ℃、45 ℃、50 ℃

中国知网　https://www.cnki.net

进行提取，实验设置三次重复。

3.3.3 料液比的单因素安排

精确称取 0.1 g 地梢瓜果实粉末于离心管中，设置提取条件为：时间 2.0 h，温度 40 ℃，溶剂浓度 0.15 mol/L，料液比分别为 1：20、1：25、1：30、1：35、1：40 进行提取，实验设置三次重复。

3.3.4 氢氧化钠浓度的单因素安排

精确称取 0.1 g 地梢瓜果实粉末于离心管中，设置提取条件为：时间 2.0 h，温度 40 ℃，料液比 1：30，溶剂浓度分别为 0.05 mol/L、0.10 mol/L、0.15 mol/L、0.20 mol/L、0.25 mol/L 进行提取，实验设置三次重复。

3.3.5 响应面法优化提取工艺

以地梢瓜果实提取液硒蛋白含量为响应值，通过实验确定最佳水平范围并进行显著性分析，以料液比、提取温度、溶剂浓度为因素，利用 Design-Expert 13 软件设计三因素三水平 BBD 响应面方法进行地梢瓜果实硒蛋白最佳提取工艺的选择，各因素设置水平如表 4 所示。

表 4 响应面设计的因素和水平

Table.4 Factors and levels of response surface design

水平	因素		
	A 料液比（%）	B 温度（℃）	C 溶剂浓度（mol/L）
-1	1:20	35	0.15
0	1:25	40	0.20
1	1:30	45	0.25

3.4 酸提法提取地梢瓜果实硒蛋白

选提取时间、提取温度、料液比、盐酸浓度作为考察对象，采用单因素实验探究各因素对地梢瓜硒蛋白提取液中硒蛋白含量的影响，各因素的考察水平见表 5。

表 5 酸提法提取地梢瓜果实可溶性硒蛋白因素考察水平

Table.5 Study on the factors of acid extraction of soluble selenoprotein from CT fruit

提取时间（h）	提取温度（℃）	料液比（g/ml）	溶剂浓度（mol/L）
1.0	30	1:20	0.05
1.5	35	1:25	0.10
2.0	40	1:30	0.15
2.5	45	1:35	0.20
3.0	50	1:40	0.25

3.4.1 提取时间的单因素安排

精确称取 0.1 g 地梢瓜果实粉末于离心管中，设置提取条件为：温度 40 ℃，料液比 1：30，溶剂浓度 0.15 mol/L，提取时间分别为 1.0 h、1.5 h、2.0 h、2.5 h、3.0 h 进

中国知网 https://www.cnki.net

行提取，实验设置三次重复。

3.4.2 提取温度的单因素安排

精确称取 0.1 g 地梢瓜果实粉末于离心管中，设置提取条件为：时间 2.0 h，料液比 1：30，溶剂浓度 0.15 mol/L，提取温度分别为 30 ℃、35 ℃、40 ℃、45 ℃、50 ℃进行提取，实验设置三次重复。

3.4.3 料液比的单因素安排

精确称取 0.1 g 地梢瓜果实粉末于离心管中，设置提取条件为：时间 2.0 h，温度 40 ℃，溶剂浓度 0.15 mol/L，料液比分别为 1：20、1：25、1：30、1：35、1：40 进行提取，实验设置三次重复。

3.4.4 盐酸浓度的单因素安排

精确称取 0.1 g 地梢瓜果实粉末于离心管中，设置提取条件为：时间 2.0 h，温度 40 ℃，料液比 1：30，溶剂浓度分别为 0.05 mol/L、0.10 mol/L、0.15 mol/L、0.20 mol/L、0.25 mol/L 进行提取，实验设置三次重复。

3.4.5 响应面法优化提取工艺

以地梢瓜果实提取液硒蛋白含量为响应值，通过实验确定最佳水平范围并进行显著性分析，以料液比、提取温度、溶剂浓度为因素，利用 Design-Expert 13 软件设计三因素三水平 BBD 响应面方法进行地梢瓜果实硒蛋白最佳提取工艺的选择，各因素设置水平如表 6 所示。

表 6 响应面设计的因素和水平

Table.6 Factors and levels of response surface design

水平	因素		
	A 料液比（%）	B 温度（℃）	C 溶剂浓度（mol/L）
-1	1:25	45	0.15
0	1:30	50	0.20
1	1:35	55	0.25

3.5 盐提法提取地梢瓜果实硒蛋白

选提取时间、提取温度、料液比、氯化钠浓度作为考察对象，采用单因素实验探究各因素对地梢瓜硒蛋白提取液中硒蛋白含量的影响，各因素的考察水平见表 7。

中国知网 https://www.cnki.net

表 7 盐提法提取地梢瓜果实可溶性硒蛋白因素考察水平

Table.7 Investigation level of salt extraction method to extract soluble selenoproteins from CT fruit

提取时间（h）	提取温度（℃）	料液比（g/ml）	溶剂浓度（mol/L）
1.0	30	1:20	0.05
1.5	35	1:25	0.10
2.0	40	1:30	0.15
2.5	45	1:35	0.20
3.0	50	1:40	0.25

3.5.1 提取时间的单因素安排

精确称取 0.1 g 地梢瓜果实粉末于离心管中，设置提取条件为：温度 40 ℃，料液比 1：30，溶剂浓度 0.15 mol/L，提取时间分别为 1.0 h、1.5 h、2.0 h、2.5 h、3.0 h 进行提取，实验设置三次重复。

3.5.2 提取温度的单因素安排

精确称取 0.1 g 地梢瓜果实粉末于离心管中，设置提取条件为：时间 2.0 h，料液比 1：30，溶剂浓度 0.15 mol/L，提取温度分别为 30 ℃、35 ℃、40 ℃、45 ℃、50 ℃进行提取。

3.5.3 料液比的单因素安排

精确称取 0.1 g 地梢瓜果实粉末于离心管中，设置提取条件为：时间 2.0 h，温度 40 ℃，溶剂浓度 0.15 mol/L，料液比分别为 1：20、1：25、1：30、1：35、1：40 进行提取。

3.5.4 氯化钠浓度的单因素安排

精确称取 0.1 g 地梢瓜果实粉末于离心管中，设置提取条件为：时间 2.0 h，温度 40 ℃，料液比 1：30，溶剂浓度分别为 0.05 mol/L、0.10 mol/L、0.15 mol/L、0.20 mol/L、0.25 mol/L 进行提取，实验设置三次重复。

3.5.5 响应面法优化提取工艺

以地梢瓜果实提取液硒蛋白含量为响应值，通过实验确定最佳水平范围并进行显著性分析，以料液比、提取温度、溶剂浓度为因素，利用 Design-Expert 13 软件设计三因素三水平 BBD 响应面方法进行地梢瓜果实硒蛋白最佳提取工艺的选择，各因素设置水平如表 8 所示。

中国知网 https://www.cnki.net

表 8 响应面设计的因素和水平

Table.8 Factors and levels of response surface design

水平	因素		
	A 料液比（%）	B 温度（℃）	C 溶剂浓度（mol/L）
-1	1:20	30	0.10
0	1:25	35	0.15
1	1:30	40	0.20

3.6 有机溶剂（乙醇）提取法提取地梢瓜果实硒蛋白

选提取时间、提取温度、料液比、乙醇浓度作为考察对象，采用单因素实验探究各因素对地梢瓜硒蛋白提取液中硒蛋白含量的影响，各因素的考察水平见表 9。

表 9 有机溶剂提取法提取地梢瓜果实可溶性硒蛋白因素考察水平

Table.9 Investigation level of organic solvent extraction method to extract soluble selenoproteins from CT fruit

提取时间（h）	提取温度（℃）	料液比（g/ml）	溶剂浓度（%）
1.0	30	1:20	60
1.5	35	1:25	65
2.0	40	1:30	70
2.5	45	1:35	75
3.0	50	1:40	80

3.6.1 提取时间的单因素安排

精确称取 0.1 g 地梢瓜果实粉末于离心管中，设置提取条件为：温度 40 ℃，料液比 1：30，溶剂浓度 70 %，提取时间分别为 1.0 h、1.5 h、2.0 h、2.5 h、3.0 h 进行提取，实验设置三次重复。

3.6.2 提取温度的单因素安排

精确称取 0.1 g 地梢瓜果实粉末于离心管中，设置提取条件为：时间 2.0 h，料液比 1：30，溶剂浓度 70%，提取温度分别为 30 ℃、35 ℃、40 ℃、45 ℃、50 ℃进行提取，实验设置三次重复。

3.6.3 料液比的单因素安排

精确称取 0.1 g 地梢瓜果实粉末于离心管中，设置提取条件为：时间 2.0 h，温度 40 ℃，溶剂浓度 70 %，料液比分别为 1：20、1：25、1：30、1：35、1：40 进行提取，实验设置三次重复。

3.6.4 乙醇浓度的单因素安排

精确称取 0.1 g 地梢瓜果实粉末于离心管中，设置提取条件为：时间 2.0 h，温度 40 ℃，料液比 1：30，溶剂浓度分别为 60 %、65 %、70 %、75 %、80 %进行提取，实验设置三次重复。

中国知网 https://www.cnki.net

3.6.5 响应面法优化提取工艺

以地梢瓜果实提取液硒蛋白含量为响应值，通过实验确定最佳水平范围并进行显著性分析，以料液比、提取温度、溶剂浓度为因素，利用 Design-Expert 13 软件设计三因素三水平 BBD 响应面方法进行地梢瓜果实硒蛋白最佳提取工艺的选择，各因素设置水平如表 10 所示。

表 10 响应面设计的因素和水平

Table.10 Factors and levels of response surface design

水平	因素		
	A 料液比（%）	B 温度（℃）	C 溶剂浓度（%）
-1	1:20	45	75
0	1:25	50	80
1	1:30	55	85

3.7 数据处理方法

Microsoft Office Excel 2010 软件、Design-Expert 13 软件处理和作图，IBM SPSS Statistics 22 软件进行差异性分析。

3.8 结果与分析

3.8.1 水提法对地梢瓜果实硒蛋白含量的影响

3.8.1.1 不同提取时间对地梢瓜果实硒蛋白含量的影响

如图 8 可知，在其他提取条件不变的情况下，地梢瓜果实提取液中可溶性硒蛋白含量随着时间的增加先增高后减少，当浸提时间达到 2 h 时，可溶性硒蛋白含量达到最高为 1.27 mg/g，因此选取 1.5 h、2 h、2.5 h 作为响应中心值。

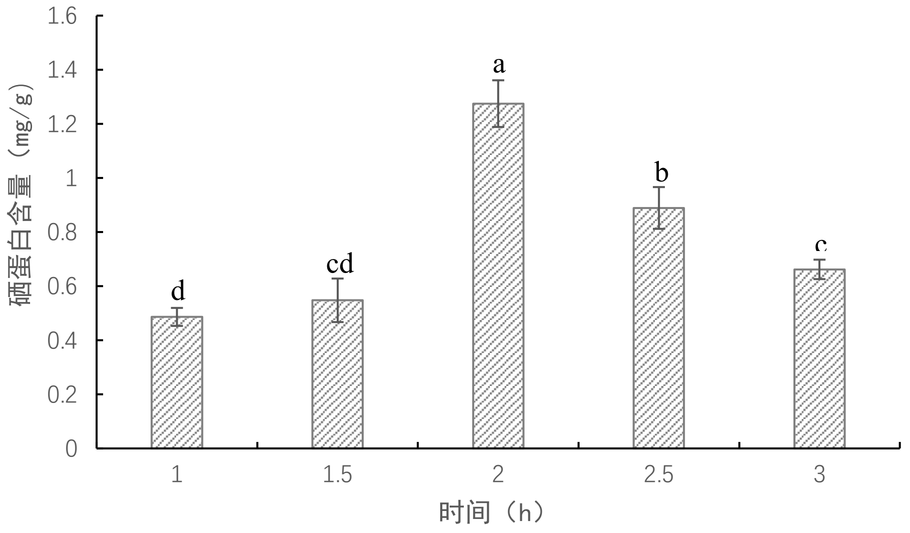

图 8 不同提取时间对地梢瓜果实可溶性硒蛋白含量的影响

Fig.8 Effect of different extraction time on soluble selenoprotein content of CT fruit

注：不同小写字母表示不同处理间在 0.05 水平下的显著差异性，下同。

中国知网 https://www.cnki.net

3.8.1.2　不同提取温度对地稍瓜果实可溶性硒蛋白含量的影响

如图 9 可知，在其他提取条件固定时，地梢瓜果实提取液中可溶性硒蛋白含量随温度的升高逐渐降低，在浸提温度为 30 ℃时，可溶性硒蛋白含量最高为 0.81 mg/g，因此选取温度 25 ℃、30 ℃、35 ℃作为响应中心值。

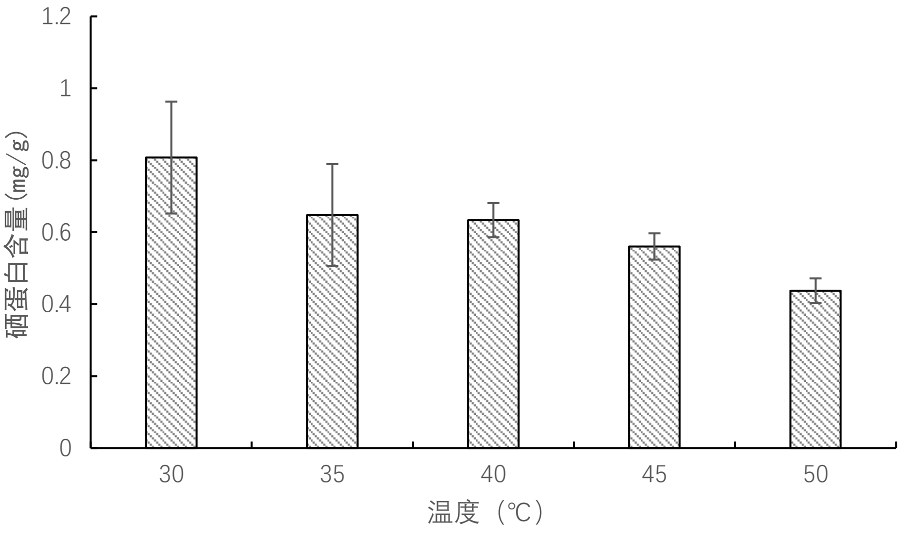

图 9　不同提取温度对地梢瓜果实可溶性硒蛋白含量的影响

Fig.9　Effect of different extraction temperatures on soluble selenoprotein content of CT fruit

3.8.1.3　不同料液比对地梢瓜果实可溶性硒蛋白含量的影响

如图 10 可知，在其他条件固定时，地梢瓜果实提取液中可溶性硒蛋白含量随料液比的降低逐渐降低，在料液比为 1：20 时可溶性硒蛋白含量最高为 2.42 mg/g，因此选取料液比 1：15、1：20、1：25 作为响应中心值。

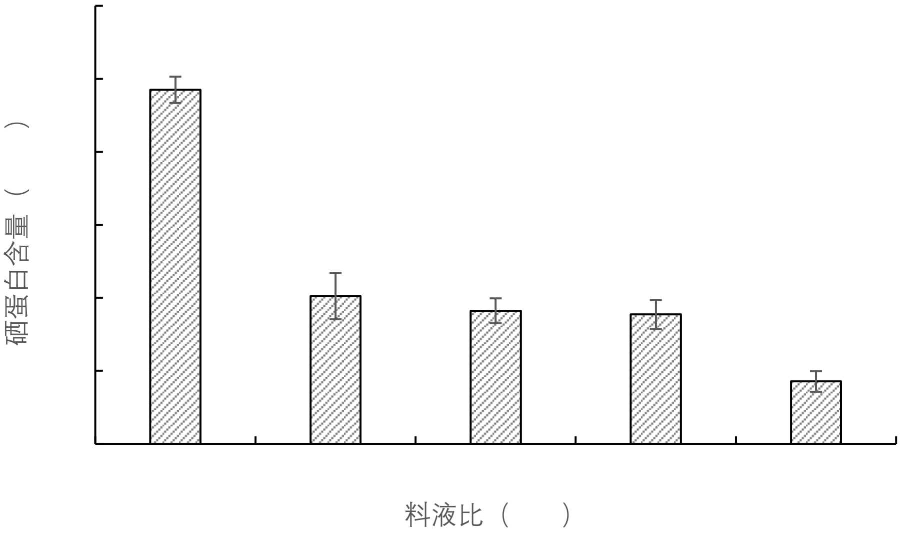

图 10　不同料液比对地梢瓜果实可溶性硒蛋白含量的影响

Fig.10　Effect of different solid-liquid ratio on soluble selenoprotein content in fruit of CT

中国知网 https://www.cnki.net

3.8.1.4 响应面法优化地梢瓜硒蛋白提取工艺

依据响应面分析方法实验设计原理，综合单因素实验结果，以浸提温度、浸提时间、料液比作为考察因素，并分别记为 A、B、C。以提取液的硒蛋白含量为相应值，分析方案见表 11 所示，结果分析见表 12 所示。

表 11 地梢瓜果实可溶性硒蛋白响应面分析方案与结果

Table.11 Response surface analysis scheme and results of soluble selenoprotein in CT fruit

实验号	因素			硒蛋白含量 Y mg/g
	A	B	C	
1	1	1	0	2.661
2	1	-1	0	1.465
3	-1	0	-1	1.631
4	0	0	0	2.684
5	0	1	-1	2.154
6	-1	1	0	1.903
7	0	0	0	2.579
8	0	-1	1	1.281
9	0	0	0	2.619
10	1	0	1	1.389
11	0	0	0	2.559
12	-1	-1	0	1.855
13	0	0	0	2.482
14	1	0	-1	2.014
15	0	1	1	1.452
16	0	-1	-1	1.370
17	-1	0	1	1.392

中国知网 https://www.cnki.net

表 12 回归模型方程的方差分析

Table.12 Variance analysis of regression model equation

来源	平方和	自由度	均方	F 值	P 值
模型	4.55	9	0.5059	106.02	< 0.0001
A-A	0.0699	1	0.0699	14.66	0.0065
B-B	0.6045	1	0.6045	126.68	< 0.0001
C-C	0.3424	1	0.3424	71.75	< 0.0001
AB	0.3295	1	0.3295	69.05	< 0.0001
AC	0.0372	1	0.0372	7.81	0.0268
BC	0.0939	1	0.0939	19.69	0.0030
A^2	0.3436	1	0.3436	72.01	< 0.0001
B^2	0.4528	1	0.4528	94.89	< 0.0001
C^2	2.02	1	2.02	423.07	< 0.0001
残差	0.0334	7	0.0048		
失拟项	0.0111	3	0.0037	0.6658	0.6155
纯误差	0.0223	4	0.0056		
总和	4.59	16			

以地梢瓜果实提取液硒蛋白含量为响应值，经过软件进行回归拟合后，得到各因素对响应值影响的回归方程为：

$$Y=2.58+0.0935\times A+0.2749\times B+0.2069\times C+0.2870\times AB-0.0965\times AC-0.1532\times BC-0.2857\times A^2-0.3279\times B^2-0.6924\times C^2$$

通过方差分析结果得到，该回归模型的P值＜0.0001达到极显著水平（P＜0.01），失拟项 P 值为 0.6155（P＞0.05）不存在显著性差异。该模型的决定系数 $R^2=0.9927$，表明 99.27 %的响应值变化都可以解释，模型与实际拟合良好，可以用来分析预测水提法提取地梢瓜果实可溶性硒蛋白含量的结果。该模型中一次项 B、C；交互项 AB；二次项 A^2、B^2、C^2 均达到了极显著水平（P＜0.01）。由 F 值可知，对地梢瓜果实提取液中可溶性硒蛋白含量的主要影响因素为 B＞C＞A，即料液比＞提取温度＞提取时间。

各因素交互作用对地梢瓜果实硒蛋白含量影响的响应面图及等高线图如图 11 所示，响应曲面越陡，等高线越接近椭圆形，说明两因素的交互作用越显著。AB、BC 坡面较为陡峭；AB、BC 等高线图更接近椭圆，表明 A 与 B 之间、A 与 C 之间具有较好的交互作用。其中考察因素 BC 的等高线图密集程度最高，表明 B、C 两因素对地梢瓜果实提取液中硒蛋白含量有明显的影响。

中国知网 https://www.cnki.net

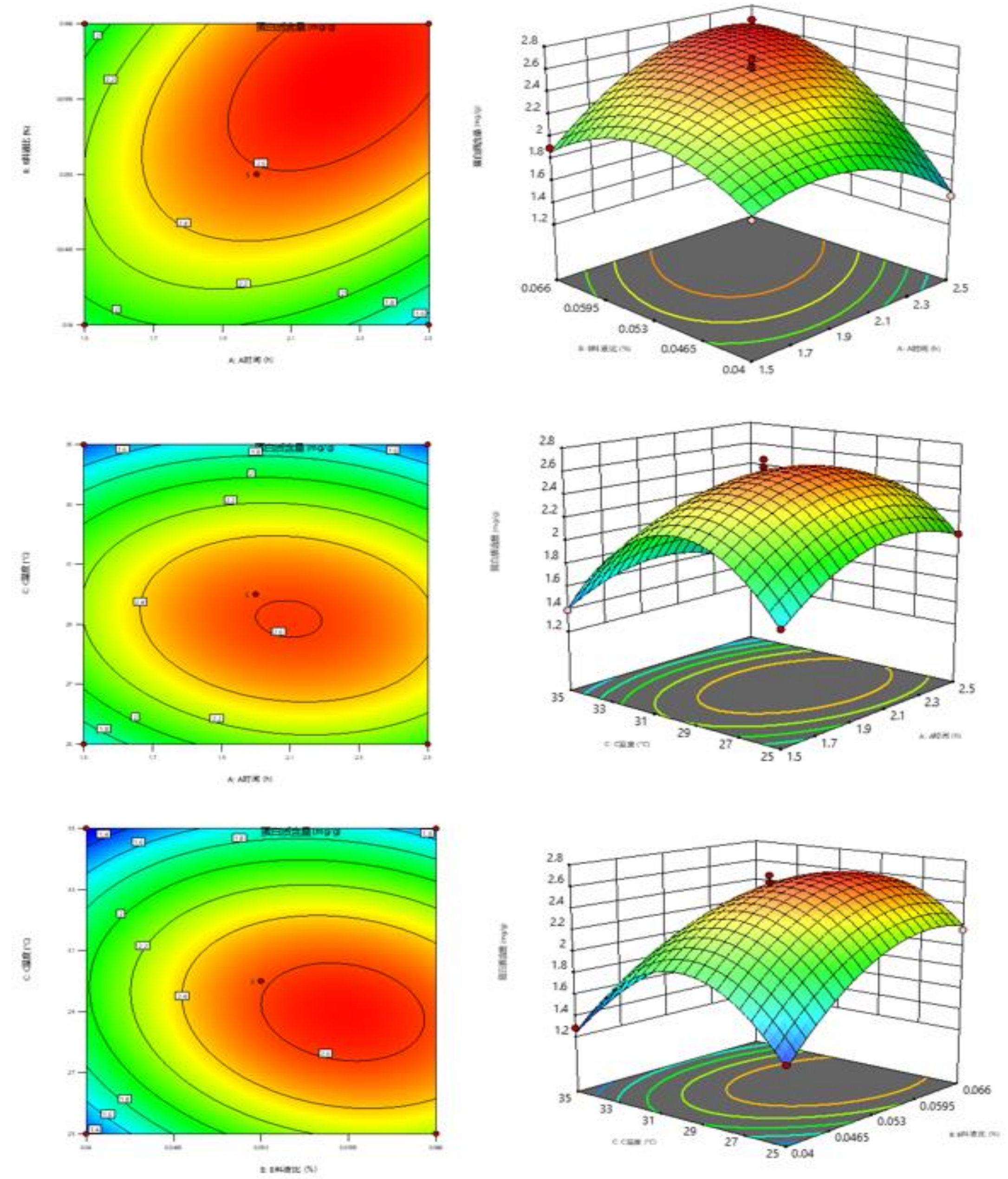

图 11　各两因素交互作用对地梢瓜果实可溶性硒蛋白含量影响的三维响应曲线图和等高线图

Fig.11　The three-dimensional response curve and contour map of the interaction of the two factors on the soluble selenoprotein content of the fruit of the CT fruit

3. 8. 1. 5　地梢瓜果实硒蛋白提取最佳工艺验证

水提法提取地梢瓜硒蛋白提取最佳工艺为：提取时间 2.29 h、料液比 1：15.87、提取温度 28.64 ℃，理论硒蛋白含量为 2.741 mg/g。为方便实验的可操作性，在进行验证实验时设定工艺参数为：提取时间 2.3 h、料液比 1：15.9、温度 28.6 ℃，实验硒蛋白含量为 2.738 mg/g 与理论预测值接近，表明此模型及提取工艺稳定性强，可操作性高，可以用于地梢瓜果实硒蛋白的提取。

中国知网　https://www.cnki.net

表 13　验证实验结果

Table.13　Verify experimental results

	时间（h）	料液比（%）	温度（℃）	硒蛋白含量（mg/g）
最佳工艺参数	2.29	1：15.87	28.64	2.741
实际工艺参数	2.3	1：15.9	28.6	2.738

3.8.2　碱提法对地稍瓜果实硒蛋白含量的影响

3.8.2.1　不同提取时间对地稍瓜果实可溶性硒蛋白含量的影响

如图 12 可知，在其他提取条件固定时，地稍瓜果实提取液中可溶性硒蛋白含量先随时间的增加逐渐增高，在 2 h 时达到最高值为 4.97 mg/g，随后其含量逐渐降低，因此选取时间 1.5 h、2 h、2.5 h 作为响应中心值。

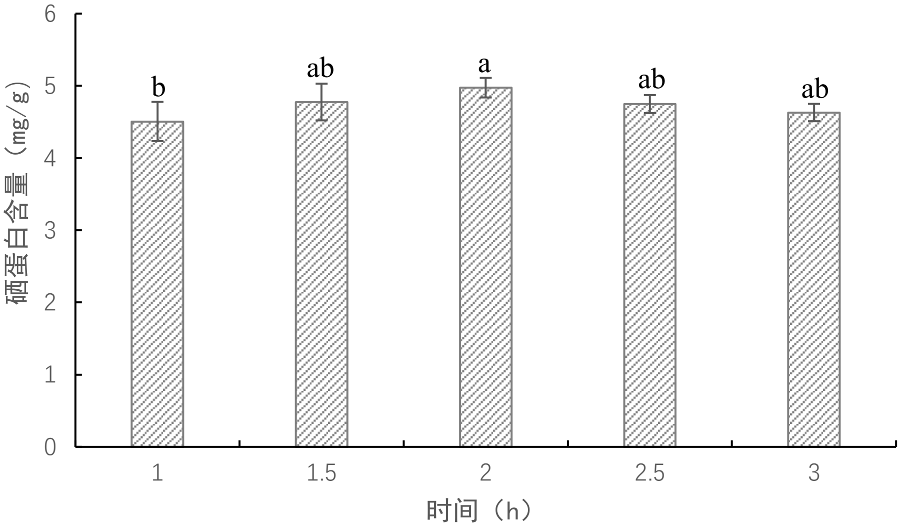

图 12　不同提取时间对地稍瓜果实可溶性硒蛋白含量的影响

Fig.12　Effect of different extraction time on soluble selenoprotein content of CT fruit

3.8.2.2　不同提取温度对地稍瓜果实可溶性硒蛋白含量的影响

如图 13 可知，在其他提取条件固定时，地稍瓜果实提取液中可溶性硒蛋白含量先随温度的升高逐渐增高，在 40 ℃时达到最高值为 5.30 mg/g，随后随温度的增加其含量开始降低，因此选取提取温度 35 ℃、40 ℃、45 ℃作为响应中心值。

中国知网　https://www.cnki.net

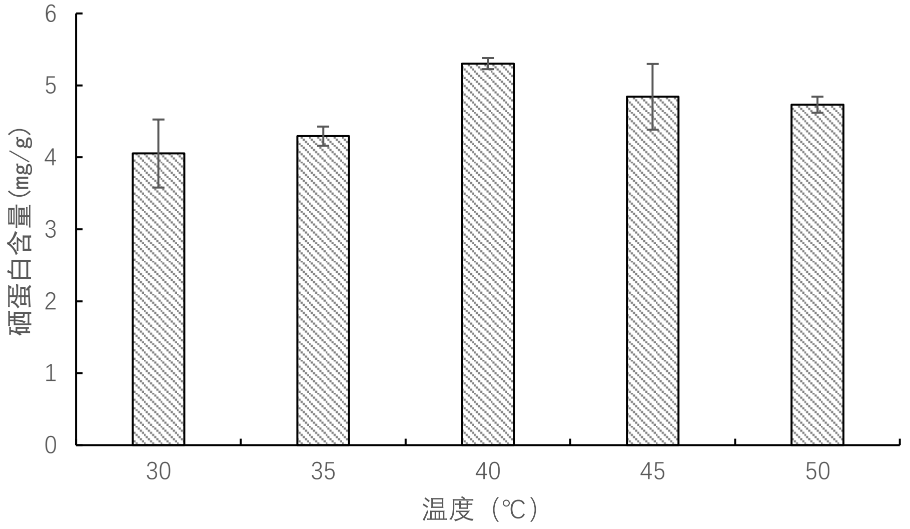

图 13　不同提取温度对地梢瓜果实可溶性硒蛋白含量的影响

Fig.13　Effect of different extraction temperatures on soluble selenoprotein content of CT fruit

3.8.2.3　不同料液比对地梢瓜果实可溶性硒蛋白含量的影响

如图 14 可知，在其他提取条件固定时，地梢瓜果实提取液中可溶性硒蛋白含量先随料液比的降低先升高后逐渐降低，在料液比为 1：25 时达到最高值为 5.05 mg/g，因此选取料液比 1：20、1：25、1：30 作为响应中心值。

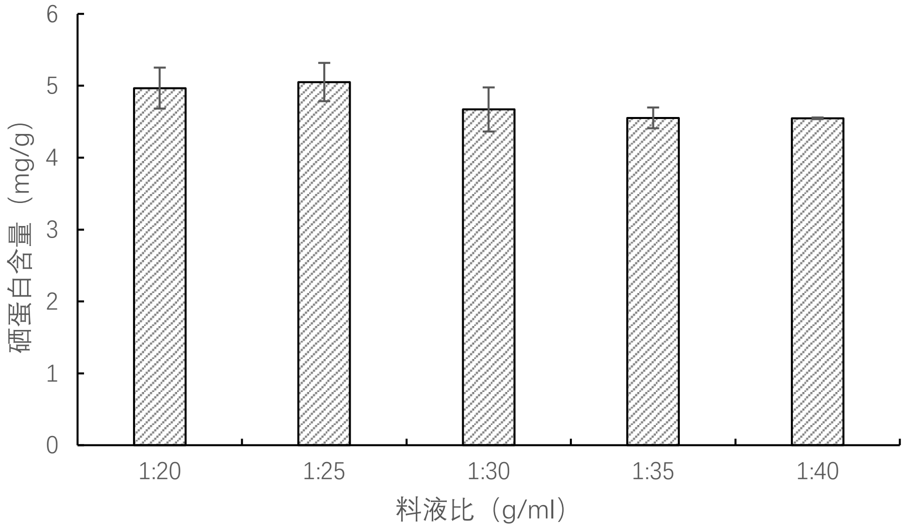

图 14　不同料液比对地梢瓜果实可溶性硒蛋白含量的影响

Fig.14　Effect of different solid-liquid ratio on soluble selenoprotein content in fruit of CT

3.8.2.4　不同氢氧化钠浓度对地梢瓜果实可溶性硒蛋白含量的影响

如图 15 可知，在其他提取条件固定时，地梢瓜果实提取液中可溶性硒蛋白含量随溶剂浓度的增加逐渐增高，在溶剂浓度为 0.2 mol/L 时达到最高值为 5.40 mg/g，因此选取溶剂浓度 0.15 mol/L、0.2 mol/L、0.25 mol/L 作为响应中心值。

中国知网 https://www.cnki.net

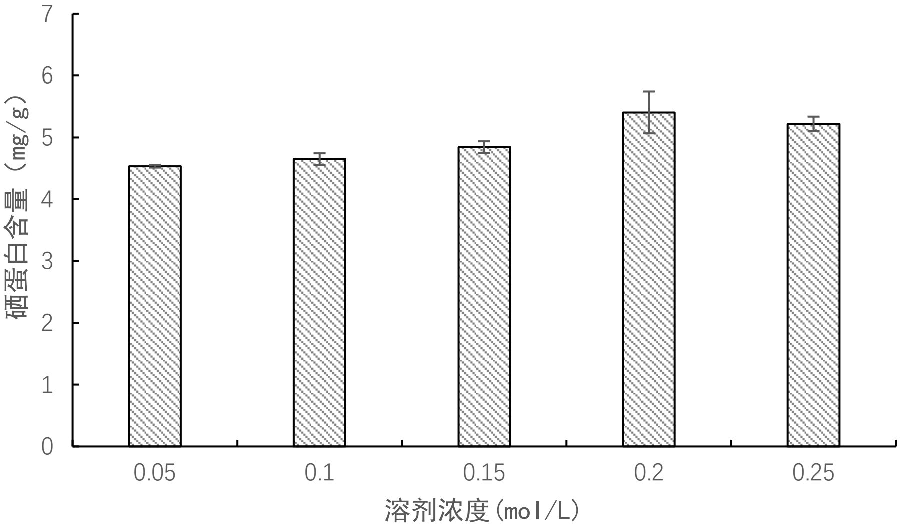

图 15 不同溶剂浓度对地稍瓜果实可溶性硒蛋白含量的影响

Fig.15 Effects of different solvent concentrations on soluble selenoprotein content in fruit of CT

3.8.2.5 响应面分析结果

依据响应面分析方法实验设计原理，综合单因素实验结果，选取料液比、浸提温度、溶剂浓度作为考察因素，并分别记为 A、B、C。以提取液的硒蛋白含量为相应值，分析方案见表 14 所示，结果分析见表 15 所示。

中国知网 https://www.cnki.net

表 14 地梢瓜果实可溶性硒蛋白响应面分析方案与结果

Table.14 Response surface analysis scheme and results of soluble selenoprotein in CT fruit

实验号	因素			硒蛋白含量 Y mg/g
	A	B	C	
1	0	1	-1	5.183
2	0	0	0	5.702
3	-1	-1	0	2.350
4	1	1	0	5.750
5	-1	1	0	5.026
6	-1	0	1	1.580
7	1	-1	0	3.993
8	0	-1	1	3.416
9	0	0	0	5.812
10	1	0	-1	5.545
11	-1	0	-1	5.092
12	0	-1	-1	5.407
13	0	0	0	5.087
14	0	0	0	5.048
15	1	0	1	5.251
16	0	0	0	5.707
17	0	1	1	5.568

表 15 回归模型方程的方差分析

Table.15 Variance analysis of regression model equation

来源	平方和	自由度	均方	F 值	P 值
模型	22.32	9	2.48	7.46	0.0074
A-A	5.27	1	5.27	15.84	0.0053
B-B	5.06	1	5.06	15.21	0.0059
C-C	3.66	1	3.66	11.01	0.0128
AB	0.2111	1	0.2111	0.6351	0.4517
AC	2.59	1	2.59	7.79	0.0269
BC	1.41	1	1.41	4.25	0.0783
A^2	3.11	1	3.11	9.35	0.0184
B^2	0.4654	1	0.4654	1.40	0.2753
C^2	0.2532	1	0.2532	0.7617	0.4117
残差	2.33	7	0.3324		
失拟项	1.78	3	0.5918	4.29	0.0967
纯误差	0.5517	4	0.1379		
总和	24.64	16			

以地梢瓜果实提取液硒蛋白含量为响应值，经过软件进行回归拟合后，得到各因

中国知网 https://www.cnki.net

素对响应值影响的回归方程为：

$Y=5.47+0.8114\times A+0.7951\times B-0.6765\times C-0.2298\times AB+0.8045\times AC+0.594\times BC-0.8590\times A^2-0.3325\times B^2-0.2452\times C^2$

通过方差分析结果得到，该回归模型的P值为0.0074达到极显著水平（P＜0.01），失拟项 P 值为 0.0967（P＞0.05）不存在显著性差异。该模型的决定系数 $R^2=0.9056$，表明 90.56 %的响应值变化都可以解释，模型与实际拟合良好，可以用来分析预测碱提法提取地梢瓜果实可溶性硒蛋白含量的结果。该模型中一次项 A、B；交互项 AC；二次项 A^2 达到了显著水平（P＜0.05）。由 F 值可知，对地梢瓜果实提取液中可溶性硒蛋白含量的主要影响因素为 A＞B＞C 即料液比＞提取温度＞溶剂浓度。

各因素交互作用对地梢瓜果实硒蛋白提取液硒蛋白含量影响的响应面图及等高线图如图 16 所示，AB、AC 坡面较为陡峭 BC 之间具有一定坡度；AB 等高线图更接近椭圆，表明 A 与 B 之间具有较好的交互作用。其中考察因素 AB 的等高线图密集程度最高，表明 A、B 两因素对地梢瓜果实提取液中硒蛋白含量有明显的影响。

中国知网 https://www.cnki.net

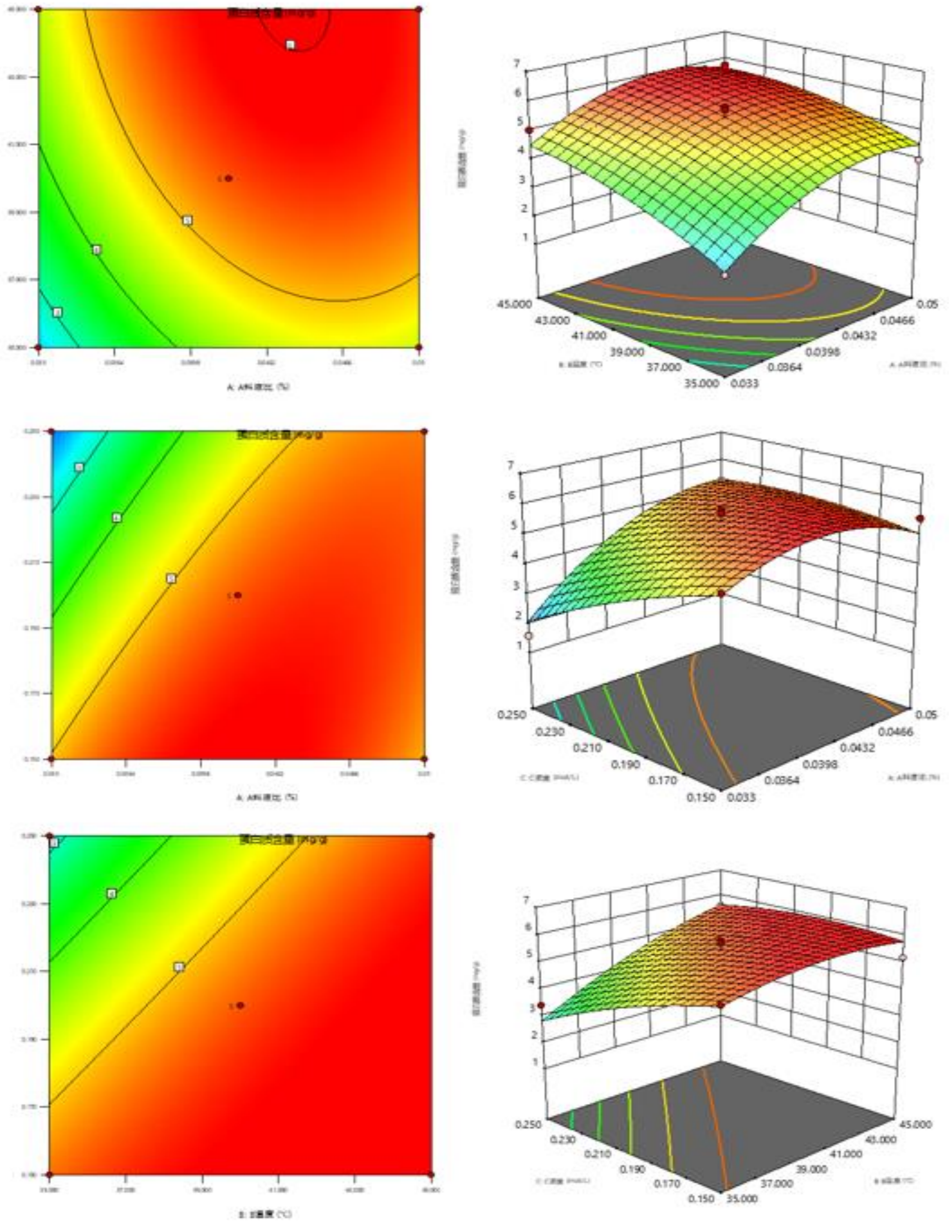

图 16　各两因素交互作用对地梢瓜果实可溶性硒蛋白含量影响的三维响应曲线图和等高线图

Fig.16　The three-dimensional response curve and contour map of the interaction of the two factors on the soluble selenoprotein content of the fruit of the CT fruit

3.8.2.6　验证实验

最佳工艺：料液比 1：22.7、提取温度 44.62 ℃、溶剂浓度 0.243 mol/L 理论硒蛋白含量为 5.925 mg/g。为方便实验的可操作性，在进行验证实验时设定工艺参数为：料液比 1：23、提取温度 44.6 ℃、溶剂浓度 0.25 mol/L，实验硒蛋白含量为 5.924 mg/g 与理论预测值接近，表明此模型及提取工艺稳定性强，可操作性高，可以用于地梢瓜果实硒蛋白的提取。

中国知网　https://www.cnki.net

表 16　验证实验结果

Table.16　Verify experimental results

	料液比（%）	温度（℃）	溶剂浓度（mol/L）	硒蛋白含量（mg/g）
最佳工艺参数	1：22.7	44.62	0.243	5.925
实际工艺参数	1：23	44.6	0.25	5.924

3.8.3　酸提法对地稍瓜果实硒蛋白含量的影响

3.8.3.1　不同提取时间对地稍瓜果实可溶性硒蛋白含量的影响

如图 17 可知，在其他提取条件固定时，地稍瓜果实提取液中可溶性硒蛋白含量先随时间的增加逐渐增高，在 2.5 h 时达到最高值为 1.63 mg/g，随后其含量降低，因此选取时间 2 h、2.5 h、3 h 作为响应中心值。

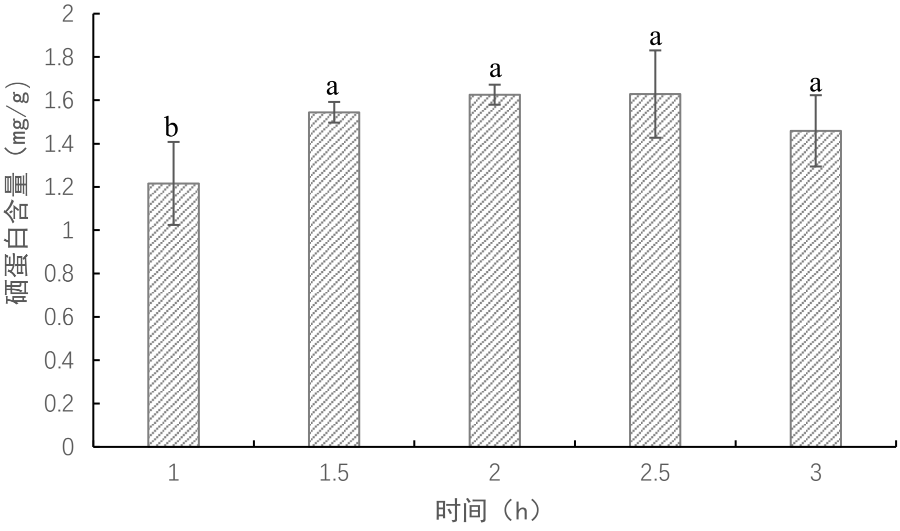

图 17　不同提取时间对地稍瓜果实可溶性硒蛋白含量的影响

Fig.17　Effect of different extraction time on soluble selenoprotein content of CT fruit

3.8.3.2　不同提取温度对地稍瓜果实可溶性硒蛋白含量的影响

如图 18 可知，在其他提取条件固定时，地稍瓜果实提取液中可溶性硒蛋白含量随温度的增高而逐渐增高，在 50 ℃时达到最高值为 1.79 mg/g，因此选取提取温度 45 ℃、50 ℃、55 ℃作为响应中心值。

中国知网　https://www.cnki.net

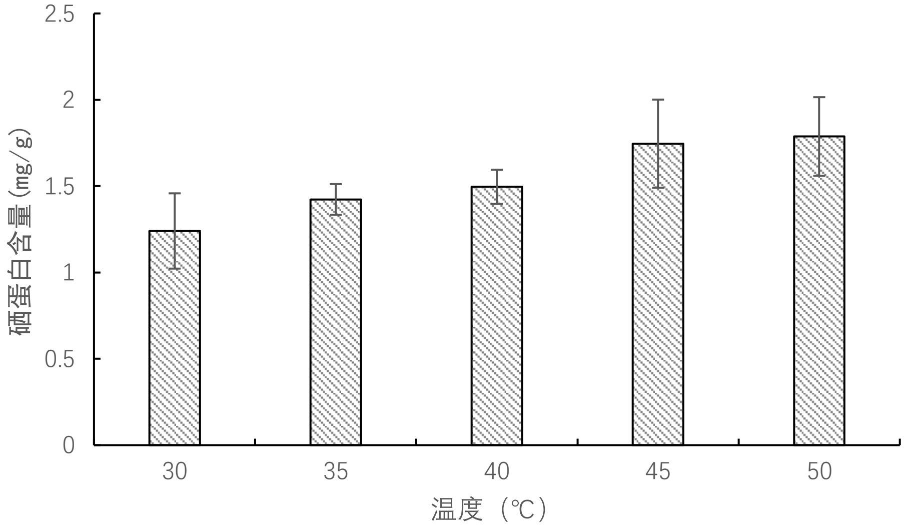

图 18 不同提取温度对地梢瓜果实可溶性硒蛋白含量的影响

Fig.18 Effect of different extraction temperatures on soluble selenoprotein content of CT fruit

3.8.3.3 不同料液比对地梢瓜果实可溶性硒蛋白含量的影响

如图 19 可知，在其他提取条件固定时，地梢瓜果实提取液中可溶性硒蛋白含量先随料液比的降低先逐渐增高，在 1：30 时达到最高值为 2.02 mg/g，随后其含量随料液比降低而降低，因此选取料液比 1：25、1：30、1：35 作为响应中心值。

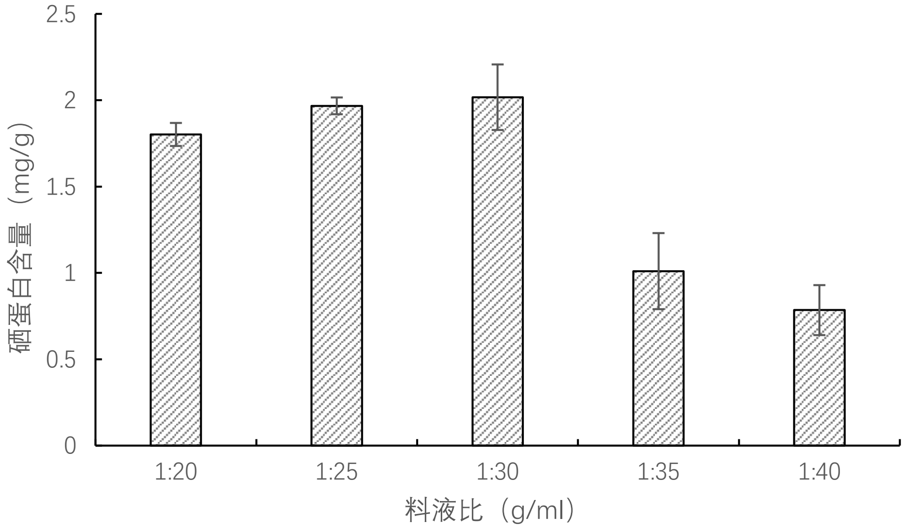

图 19 不同料液比对地梢瓜果实可溶性硒蛋白含量的影响

Fig.19 Effect of different solid-liquid ratio on soluble selenoprotein content in fruit of CT

3.8.3.4 不同盐酸浓度对地梢瓜果实可溶性硒蛋白含量的影响

如图 20 可知，在其他提取条件固定时，地梢瓜果实提取液中可溶性硒蛋白含量先随溶剂浓度的增高逐渐增高，在 0.2 mol/L 时达到最高值为 3.56 mg/g，随后其含量降低，因此选取溶剂浓度 0.15 mol/L、0.2 mol/L、0.25 mol/L 作为响应中心值。

中国知网 https://www.cnki.net

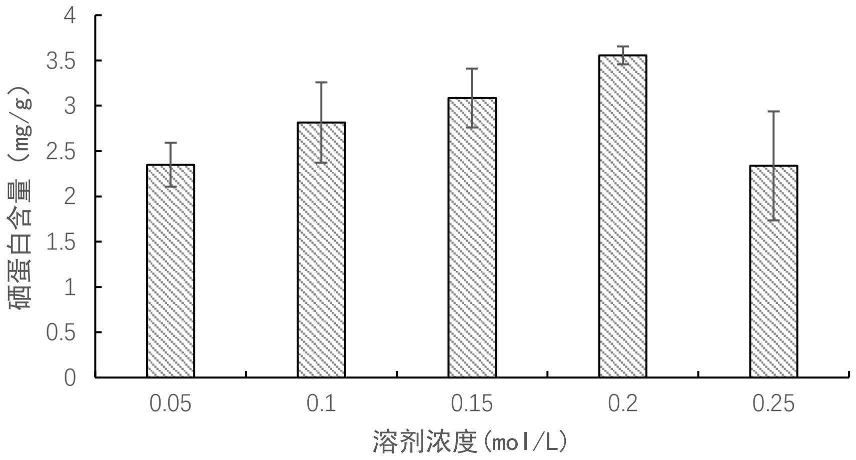

图 20 不同溶剂浓度对地梢瓜果实可溶性硒蛋白含量的影响

Fig.20 Effects of different solvent concentrations on soluble selenoprotein content in fruit of CT

3.8.3.5 响应面分析结果

依据响应面分析方法实验设计原理，综合单因素实验结果，选取料液比、浸提温度、溶剂浓度作为考察因素，并分别记为 A、B、C。以提取液的硒蛋白质含量为相应值，分析方案见表 17 所示，结果分析见表 18 所示。

表 17 地梢瓜果实可溶性硒蛋白响应面分析方案与结果

Table.17 Response surface analysis scheme and results of soluble selenoprotein in CT fruit

实验号	因素			硒蛋白含量 Y mg/g
	A	B	C	
1	0	-1	1	2.203
2	0	0	0	2.689
3	0	0	0	2.693
4	1	-1	0	1.987
5	-1	0	1	1.873
6	0	1	1	2.275
7	1	0	1	1.834
8	1	1	0	2.413
9	0	0	0	2.894
10	1	0	-1	2.173
11	0	0	0	2.688
12	0	0	0	2.691
13	0	-1	-1	2.133
14	0	1	-1	2.322
15	-1	0	-1	2.182
16	-1	-1	0	1.710
17	-1	1	0	2.141

中国知网 https://www.cnki.net

表 18 回归模型方程的方差分析

Table.18 Variance analysis of regression model equation

来源	平方和	自由度	均方	F 值	P 值
模型	1.73	9	0.1926	7.55	0.0071
A-A	0.0314	1	0.0314	1.23	0.3039
B-B	0.1562	1	0.1562	6.13	0.0425
C-C	0.0488	1	0.0488	1.92	0.2089
AB	6.250E-06	1	6.250E-06	0.0002	0.9879
AC	0.0002	1	0.0002	0.0088	0.9278
BC	0.0034	1	0.0034	0.1342	0.7249
A^2	0.8263	1	0.8263	32.41	0.0007
B^2	0.2136	1	0.2136	8.38	0.0232
C^2	0.3127	1	0.3127	12.26	0.0100
残差	0.1785	7	0.0255		
失拟项	0.1452	3	0.0484	5.83	0.0608
纯误差	0.0332	4	0.0083		
总和	1.91	16			

以地梢瓜果实提取液硒蛋白含量为响应值，经过软件进行回归拟合后，得到各因素对响应值影响的回归方程为：

$$Y=2.73+0.0626\times A+0.1398\times B-0.07811\times C-0.0013\times AB-0.00755\times AC-0.0292\times BC-0.4430\times A^2-0.2253\times B^2-0.2725\times C^2$$

通过方差分析结果得到，该回归模型的P值为0.0071达到极显著水平（$P<0.01$），失拟项 P 值为 0.0608（$P>0.05$）不存在显著性差异。该模型的决定系数 $R^2=0.9067$，表明 90.67 %的响应值变化都可以解释，模型与实际拟合良好，可以用来分析预测酸提法提取地梢瓜果实可溶性硒蛋白含量的结果。该模型二次项 A^2、C^2 均达到了显著水平（$P<0.05$）。由 F 值可知，对地梢瓜果实提取液中可溶性硒蛋白含量的主要影响因素为 B＞C＞A，即提取温度＞溶剂浓度＞料液比。

各因素交互作用对地梢瓜果实硒蛋白提取液硒蛋白含量影响的响应面图及等高线图如图 21 所示，AB、AC 坡面较为陡峭 BC 之间具有一定坡度；AB、AC 等高线图更接近椭圆，表明 A 与 B 之间，A 与 C 之间具有较好的交互作用。其中考察因素 AB、AC 的等高线图密集程度较高，表明 A、B、C 三因素对地梢瓜果实提取液中硒蛋白含量均有明显的影响。

中国知网 https://www.cnki.net

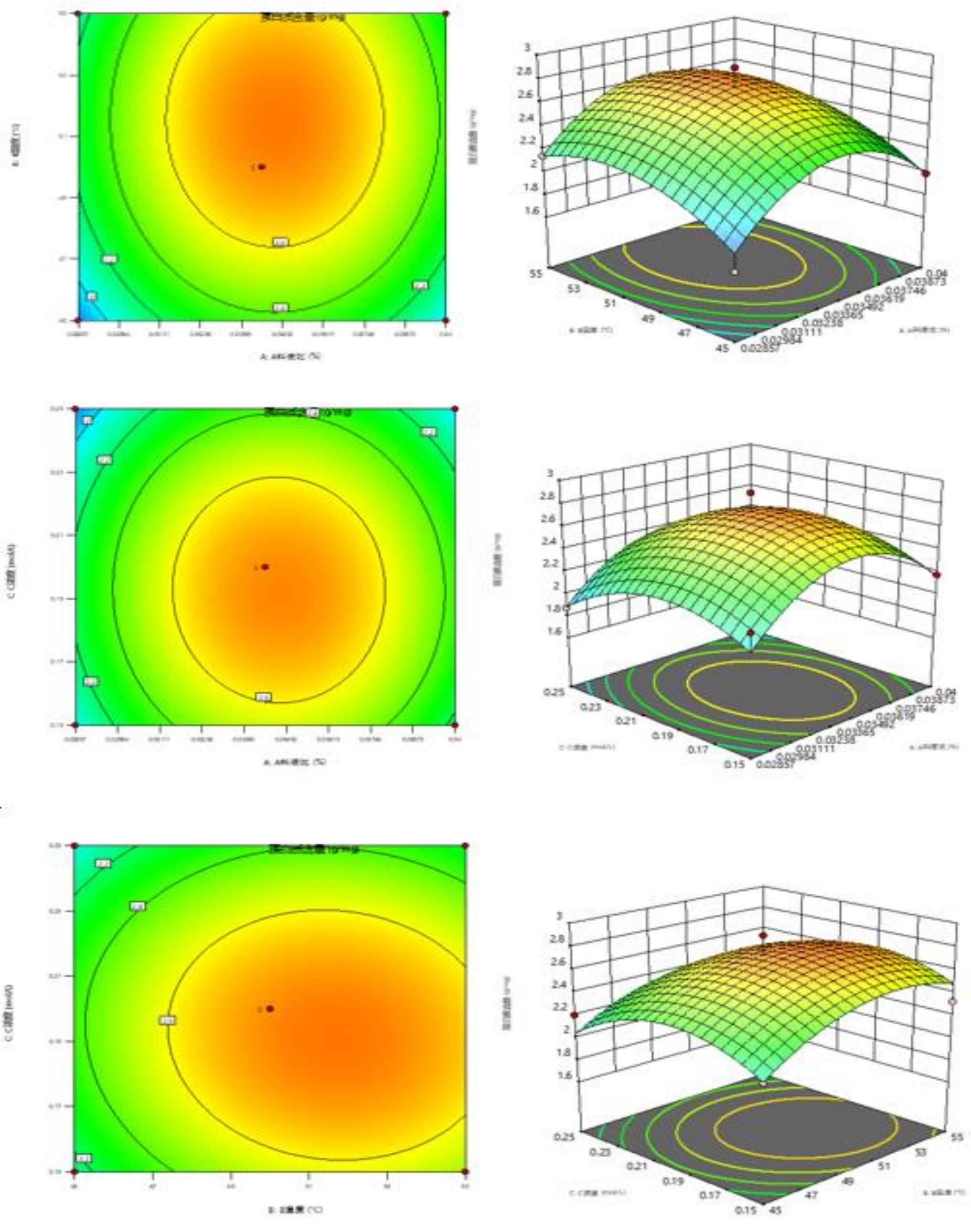

图 21　各两因素交互作用对地梢瓜果实可溶性硒蛋白含量影响的三维响应曲线图和等高线图

Fig.21　The three-dimensional response curve and contour map of the interaction of the two factors on the soluble selenoprotein content of the fruit of the CT fruit

3.8.3.6　验证实验

最佳工艺：料液比 1：28.6、提取温度 51.603 ℃、溶剂浓度 0.192 mol/L 理论硒蛋白含量为 2.762 mg/g。为方便实验的可操作性，在进行验证实验时设定工艺参数为：料液比 1：29、提取温度 51.7 ℃、溶剂浓度 0.19 mol/L，实验硒蛋白含量为 2.761 mg/g 与理论预测值接近，表明此模型及提取工艺稳定性强，可操作性高，可以用于地梢瓜果实硒蛋白的提取。

中国知网 https://www.cnki.net

表 19 验证实验结果

Table.19 Verify experimental results

	料液比（%）	温度（℃）	溶剂浓度（mol/L）	硒蛋白含量（mg/g）
最佳工艺参数	1：28.6	51.603	0.192	2.762
实际工艺参数	1：29	51.7	0.19	2.761

3.8.4 盐提法对地梢瓜果实硒蛋白含量的影响

3.8.4.1 不同提取时间对地梢瓜果实可溶性硒蛋白含量的影响

如图 22 可知，在其他提取条件固定时，地梢瓜果实提取液中可溶性硒蛋白含量先随时间的增加逐渐增高，在 1.5 h 时达到最高值为 2.529 mg/g，随后其含量随时间的增加逐渐降低，因此选取时间 1 h、1.5 h、2 h 作为响应中心值。

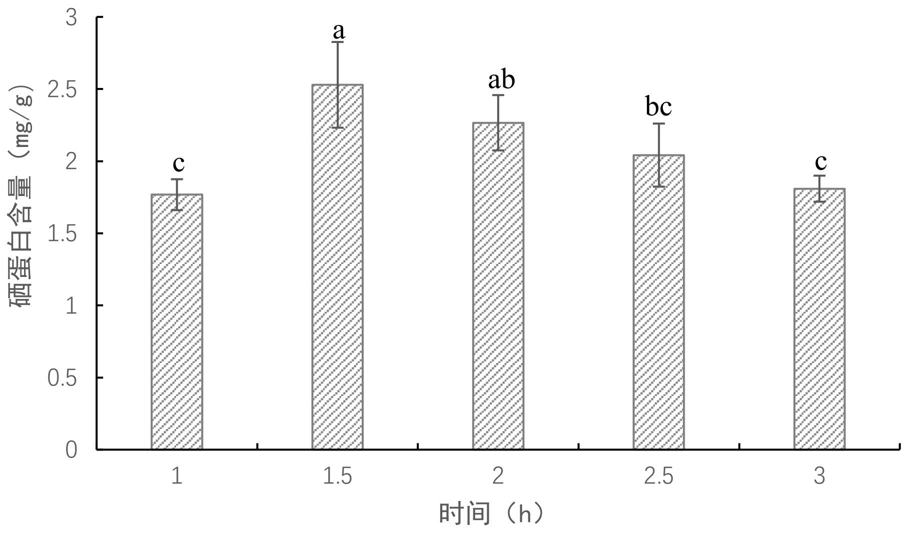

图 22 不同提取时间对地梢瓜果实可溶性硒蛋白含量的影响

Fig.22 Effect of different extraction time on soluble selenoprotein content of CT fruit

3.8.4.2 不同提取温度对地梢瓜果实可溶性硒蛋白含量的影响

如图 23 可知，在其他提取条件固定时，地梢瓜果实提取液中可溶性硒蛋白含量先随提取温度的增加逐渐增高，在 35 ℃时达到最高值为 2.79 mg/g，随后其含量随提取温度的增加逐渐降低，因此选取时间 30 ℃、35 ℃、40 ℃作为响应中心值。

中国知网 https://www.cnki.net

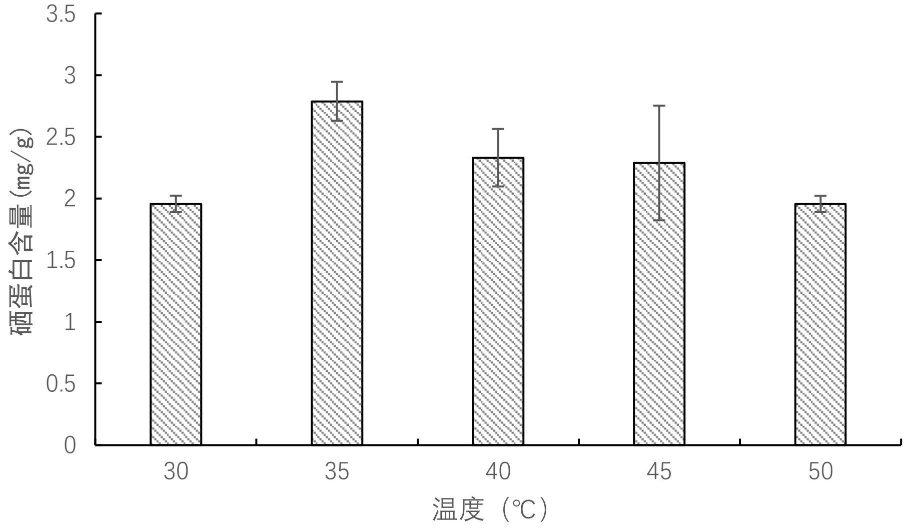

图 23 不同提取温度对地梢瓜果实可溶性硒蛋白含量的影响

Fig.23 Effect of different extraction temperatures on soluble selenoprotein content of CT fruit

3.8.4.3 料液比对地梢瓜果实可溶性硒蛋白含量的影响

如图 24 可知，在其他提取条件固定时，地梢瓜果实提取液中可溶性硒蛋白含量先随料液比的降低逐渐增高，在 1：25 时达到最高值为 2.81 mg/g，随后其含量随料液比的降低逐渐降低，因此选取时间 1：20、1：25、1：30 作为响应中心值。

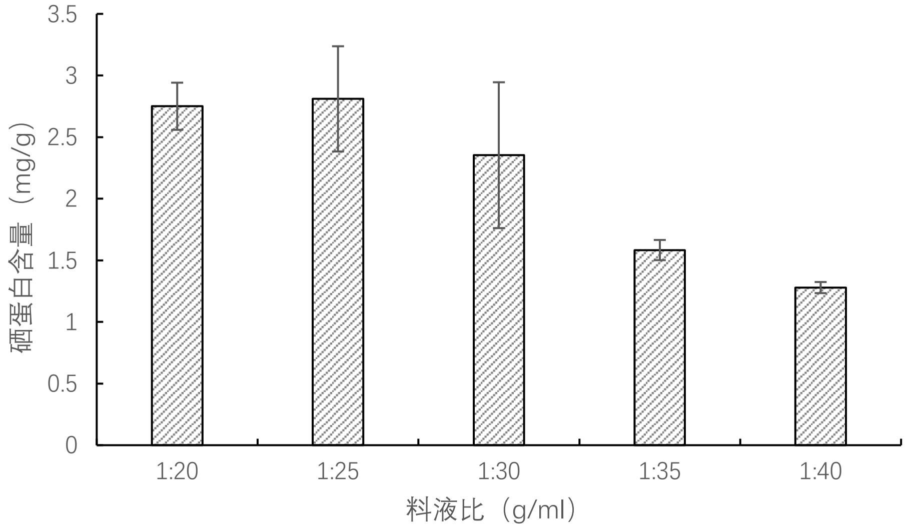

图 24 不同料液比对地梢瓜果实可溶性硒蛋白含量的影响

Fig.24 Effect of different solid-liquid ratio on soluble selenoprotein content in fruit of CT

3.8.4.4 不同氯化钠浓度对地梢瓜果实可溶性硒蛋白含量的影响

如图 25 可知，在其他提取条件固定时，地梢瓜果实提取液中可溶性硒蛋白含量先溶剂浓度的增加逐渐增高，在 0.15 mol/L 时达到最高值为 3.94 mg/g，随后其含量随提取温度的增加逐渐降低，因此选取时间 0.1 mol/L、0.15 mol/L、0.2 mol/L 作为响

中国知网 https://www.cnki.net

应中心值。

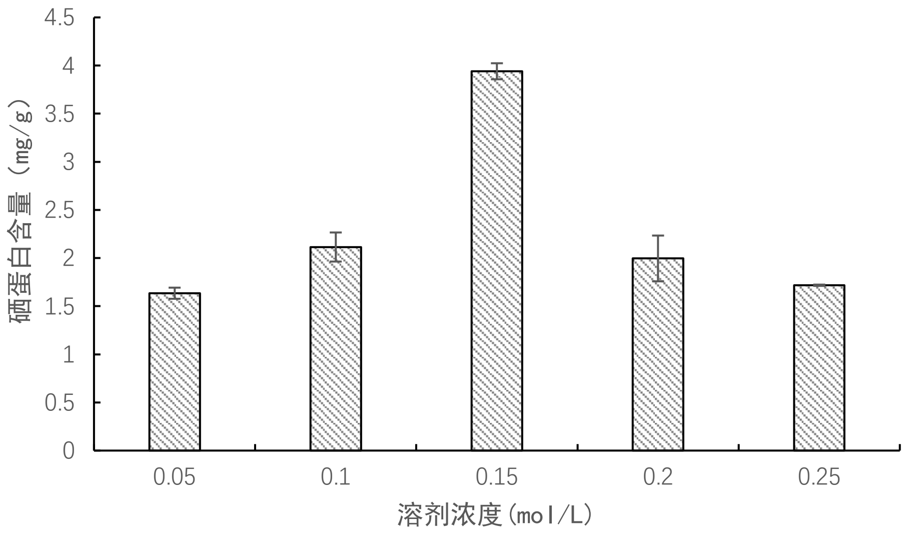

图 25 不同溶剂浓度对地梢瓜果实可溶性硒蛋白含量的影响

Fig.25 Effects of different solvent concentrations on soluble selenoprotein content in fruit of CT

3.8.4.5 响应面分析结果

依据响应面分析方法实验设计原理，综合单因素实验结果，选取料液比、浸提温度、溶剂浓度作为考察因素，并分别记为 A、B、C。以提取液的硒蛋白含量为相应值，分析方案见表 20 所示，结果分析见表 21 所示。

中国知网 https://www.cnki.net

表 20 地稍瓜果实可溶性硒蛋白响应面分析方案与结果

Table.20 Response surface analysis scheme and results of soluble selenoprotein in CT fruit

实验号	因素			硒蛋白含量 Y mg/g
	A	B	C	
1	0	-1	1	1.719
2	0	0	0	5.973
3	0	1	-1	1.960
4	1	0	-1	1.277
5	0	-1	-1	1.576
6	1	-1	0	3.441
7	-1	1	0	4.146
8	0	1	1	6.057
9	-1	0	1	1.419
10	0	0	0	5.831
11	1	0	1	1.558
12	0	0	0	5.867
13	0	0	0	3.773
14	-1	0	-1	1.124
15	0	0	0	5.976
16	1	1	0	1.774
17	-1	-1	0	3.150

表 21 回归模型方程的方差分析

Table.21 Variance analysis of regression model equation

来源	平方和	自由度	均方	F 值	P 值
模型	51.17	9	5.69	4.11	0.0380
A-A	0.4001	1	0.4001	0.2889	0.6076
B-B	2.05	1	2.05	1.48	0.2630
C-C	2.90	1	2.90	2.09	0.1912
AB	1.77	1	1.77	1.28	0.2951
AC	0.0000	1	0.0000	0.0000	0.9954
BC	3.91	1	3.91	2.82	0.1368
A^2	15.52	1	15.52	11.21	0.0123
B^2	0.8018	1	0.8018	0.5790	0.4716
C^2	20.74	1	20.74	14.98	0.0061
残差	9.69	7	1.38		
失拟项	6.02	3	2.01	2.1	0.2327
纯误差	3.68	4	0.9190		
总和	60.86	16			

以地梢瓜果实提取液硒蛋白含量为响应值，经过软件进行回归拟合后，得到各因

中国知网 https://www.cnki.net

素对响应值影响的回归方程为：

$$Y=-87.944+2728.72\times A+1.380\times B+140.347\times C-15.665\times AB-8.235\times AC+3.95400\times BC-26572.664\times A^2-0.018\times B^2-887.850\times C^2$$

通过方差分析结果得到，该回归模型的 P 值为 0.038 达到显著水平（$P<0.05$），失拟项 P 值为 0.2327（$P>0.05$）不存在显著性差异。该模型的决定系数 $R^2=0.8407$，表明 84.07 %的响应值变化都可以解释，模型与实际拟合良好，可以用来分析预测盐提法提取地梢瓜果实可溶性硒蛋白含量的结果。该模型二次项 A^2、C^2 均达到了显著水平（$P<0.05$）。由 F 值可知，对地梢瓜果实提取液中可溶性硒蛋白含量的主要影响因素为 C＞B＞A，即溶剂浓度＞提取温度＞料液比。

各因素交互作用对地梢瓜果实硒蛋白提取液硒蛋白含量影响的响应面图及等高线图如图 26 所示，AC 坡面较为陡峭 AB、BC 之间具有一定坡度；AB、BC 等高线图更接近椭圆，表明 A 与 B 之间，B 与 C 之间具有较好的交互作用。其中考察因素 AB、BC 的等高线图密集程度较高，表明 A、C 两因素对地梢瓜果实提取液中硒蛋白含量均有明显的影响。

中国知网 https://www.cnki.net

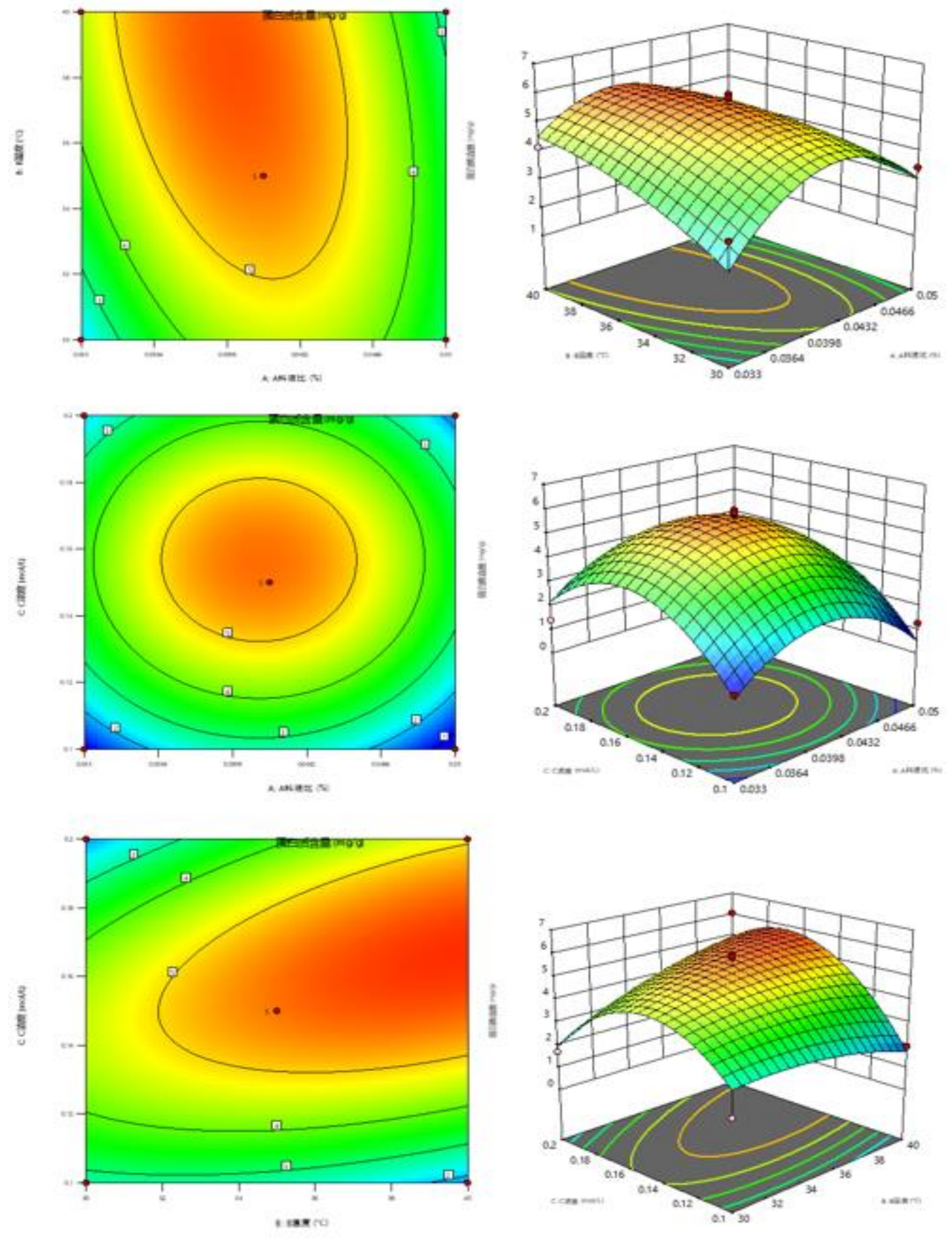

图 26 各两因素交互作用对地稍瓜果实可溶性硒蛋白含量影响的三维响应曲线图和等高线图

Fig.26 The three-dimensional response curve and contour map of the interaction of the two factors on the soluble selenoprotein content of the fruit of the CT fruit

3.8.4.6 验证实验

最佳工艺：料液比 1：21.74、提取温度 35.99 ℃、溶剂浓度 0.141 mol/L 理论硒蛋白含量为 4.731 mg/g。为方便实验的可操作性，在进行验证实验时设定工艺参数为：料液比 1：22、提取温度 36 ℃、溶剂浓度 0.14 mol/L，实验硒蛋白含量为 4.730 mg/g 与理论预测值接近，表明此模型及提取工艺稳定性强，可操作性高，可以用于地稍瓜

中国知网 https://www.cnki.net

果实硒蛋白的提取。

表 22　验证实验结果

Table.22　Verify experimental results

	料液比（%）	温度（℃）	溶剂浓度（mol/L）	硒蛋白含量（mg/g）
最佳工艺参数	1：21.74	35.99	0.141	4.731
实际工艺参数	1：22	36	0.14	4.730

3.8.5　有机溶剂提取法对地梢瓜果实硒蛋白含量的影响

3.8.5.1　不同提取时间对地梢瓜果实可溶性硒蛋白含量的影响

如图 27 可知，在其他提取条件固定时，地梢瓜果实提取液中可溶性硒蛋白含量先随时间的增加逐渐增高，在 2.5 h 时达到最高值为 1.15 mg/g，随后其含量随时间的增加逐渐降低，因此选取时间 2 h、2.5 h、3 h 作为响应中心值。

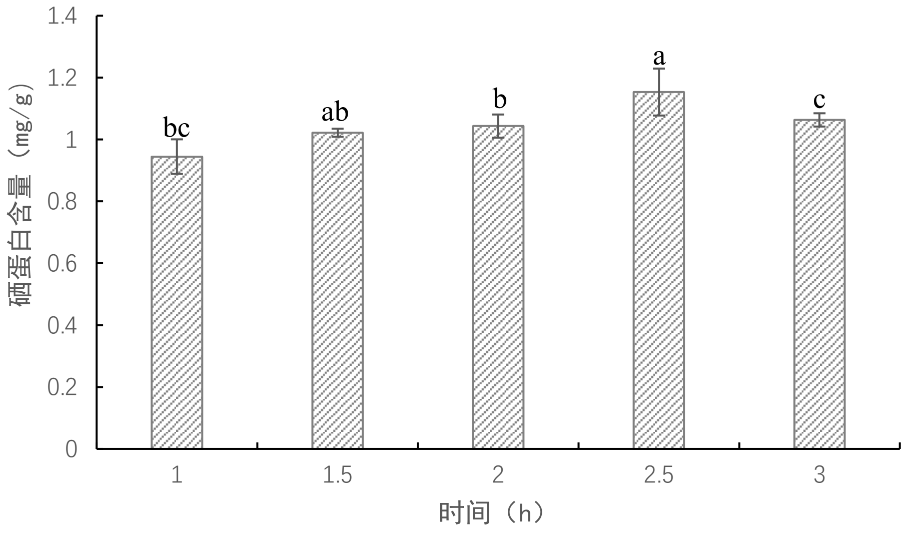

图 27　不同提取时间对地梢瓜果实可溶性硒蛋白含量的影响

Fig.27　Effect of different extraction time on soluble selenoprotein content of CT fruit

3.8.5.2 不同提取温度对地梢瓜果实可溶性硒蛋白含量的影响

如图 28 可知，在其他提取条件固定时，地梢瓜果实提取液中可溶性硒蛋白含量先随提取温度的升高逐渐增高，在 50 ℃时达到最高值为 1.47 mg/g，因此选取提取温度 45 ℃、50 ℃、55 ℃作为响应中心值。

中国知网　https://www.cnki.net

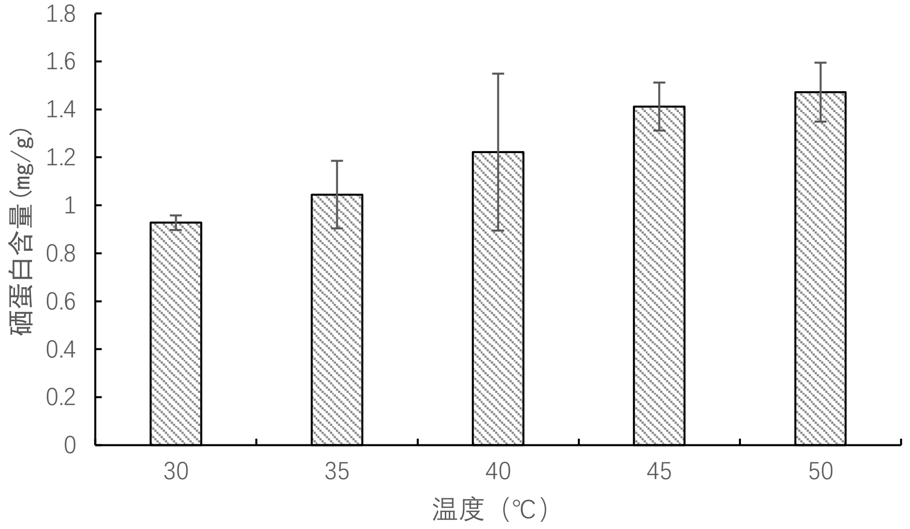

图 28 不同提取温度对地梢瓜果实可溶性硒蛋白含量的影响

Fig.28 Effect of different extraction temperatures on soluble selenoprotein content of CT fruit

3.8.5.3 不同料液比对地梢瓜果实可溶性硒蛋白含量的影响

如图 29 可知，在其他提取条件固定时，地梢瓜果实提取液中可溶性硒蛋白含量先随料液比的降低逐渐增高，在 1：25 时达到最高值为 1.45 mg/g，随后其含量随料液比的降低逐渐降低，因此选取料液比 1：20、1：25、1：30 作为响应中心值。

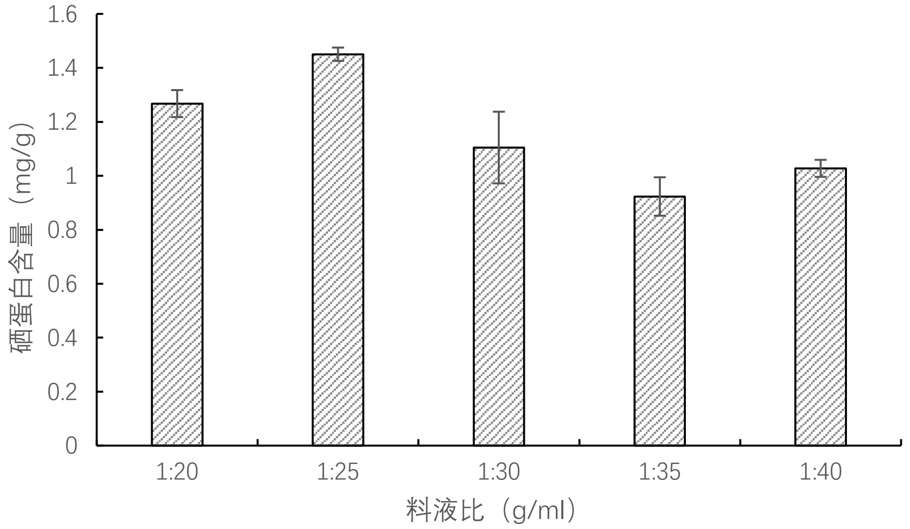

图 29 不同料液比对地梢瓜果实可溶性硒蛋白含量的影响

Fig.29 Effect of different solid-liquid ratio on soluble selenoprotein content in fruit of CT

3.8.5.4 不同乙醇浓度对地梢瓜果实可溶性硒蛋白含量的影响

如图 30 可知，在其他提取条件固定时，地梢瓜果实提取液中可溶性硒蛋白含量先溶剂浓度升高而逐渐增高，在 80 h 时达到最高值为 2.06 mg/g 因此选取溶剂浓度 75 %、80 %、85 %作为响应中心值。

中国知网 https://www.cnki.net

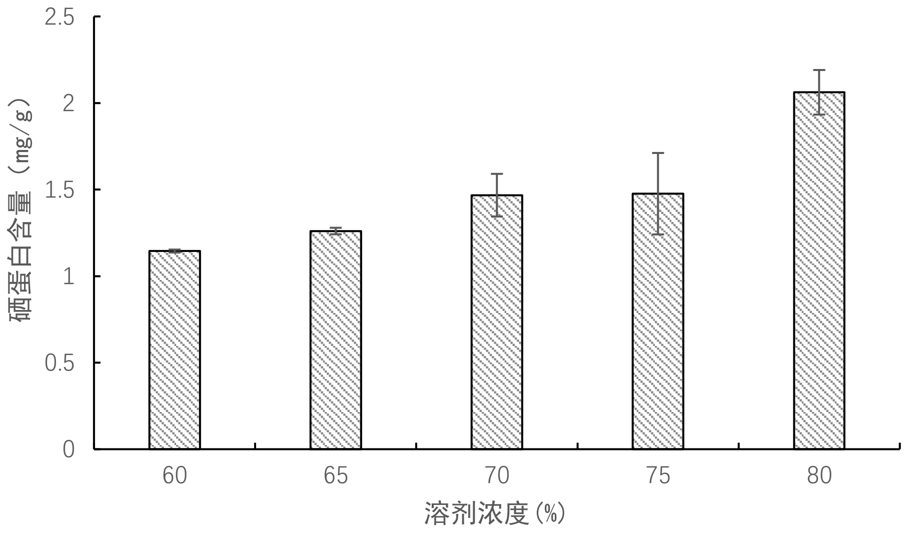

图 30　不同溶剂浓度对地梢瓜果实可溶性硒蛋白含量的影响

Fig.30　Effects of different solvent concentrations on soluble selenoprotein content in fruit of CT

3.8.5.5 响应面分析结果

依据响应面分析方法实验设计原理，综合单因素实验结果，选取料液比、浸提温度、溶剂浓度作为考察因素，并分别记为 A、B、C。以提取液的硒蛋白含量为相应值，分析方案见表 23 所示，结果分析见表 24 所示。

中国知网 https://www.cnki.net

表 23 地稍瓜果实可溶性硒蛋白响应面分析方案与结果

Table.23 Response surface analysis scheme and results of soluble selenoprotein in CT fruit

实验号	因素			硒蛋白含量 Y mg/g
	A	B	C	
1	0	1	-1	2.293
2	1	0	1	2.496
3	0	-1	-1	1.711
4	0	0	0	3.159
5	0	0	0	3.186
6	-1	-1	0	1.843
7	1	-1	0	2.794
8	-1	0	-1	1.707
9	0	0	0	3.244
10	0	0	0	3.222
11	1	0	-1	1.889
12	0	1	1	2.933
13	1	1	0	2.963
14	0	0	0	2.942
15	0	-1	1	2.578
16	-1	1	0	2.505
17	-1	0	1	2.424

表 24 回归模型方程的方差分析

Table.24 Variance analysis of regression model equation

来源	平方和	自由度	均方	F 值	P 值
模型	51.17	9	5.69	4.11	0.0380
A-A	0.4001	1	0.4001	0.2889	0.6076
B-B	2.05	1	2.05	1.48	0.2630
C-C	2.90	1	2.90	2.09	0.1912
AB	1.77	1	1.77	1.28	0.2951
AC	0.0000	1	0.0000	0.0000	0.9954
BC	3.91	1	3.91	2.82	0.1368
A^2	15.52	1	15.52	11.21	0.0123
B^2	0.8018	1	0.8018	0.5790	0.4716
C^2	20.74	1	20.74	14.98	0.0061
残差	9.69	7	1.38		
失拟项	6.02	3	2.01	2.1	0.2327
纯误差	3.68	4	0.9190		
总和	60.86	16			

以地梢瓜果实提取液硒蛋白含量为响应值，经过软件进行回归拟合后，得到各因

中国知网 https://www.cnki.net

素对响应值影响的回归方程为：

$Y=-87.944+2728.72\times A+1.380\times B+140.347\times C-15.665\times AB-8.235\times AC+3.95400\times BC-26572.664\times A^2-0.018\times B^2-887.850\times C^2$

通过方差分析结果得到，该回归模型的P值为0.0009达到极显著水平（P＜0.01），失拟项 P 值为 0.1101（P＞0.05）不存在显著性差异。该模型的决定系数 $R^2=0.9504$，表明 95.04 %的响应值变化都可以解释，模型与实际拟合良好，可以用来分析预测盐提法提取地梢瓜果实可溶性硒蛋白含量的结果。该模型的一次项 C，二次项 A^2、C^2 均达到了显著水平（P＜0.05）。由 F 值可知，对地梢瓜果实提取液中可溶性硒蛋白含量的主要影响因素为 C＞B＞A，即溶剂浓度＞提取温度＞料液比。

各因素交互作用对地梢瓜果实硒蛋白提取液硒蛋白含量影响的响应面图及等高线图如图 31 所示，AC、BC 坡面较为陡峭 AB 之间具有一定坡度；AB、BC 等高线图更接近椭圆，表明 A 与 B 之间，B 与 C 之间具有较好的交互作用。其中考察因素 AB 的等高线图密集程度较高，表明 A、B 两因素对地梢瓜果实提取液中硒蛋白含量均有明显的影响。

中国知网 https://www.cnki.net

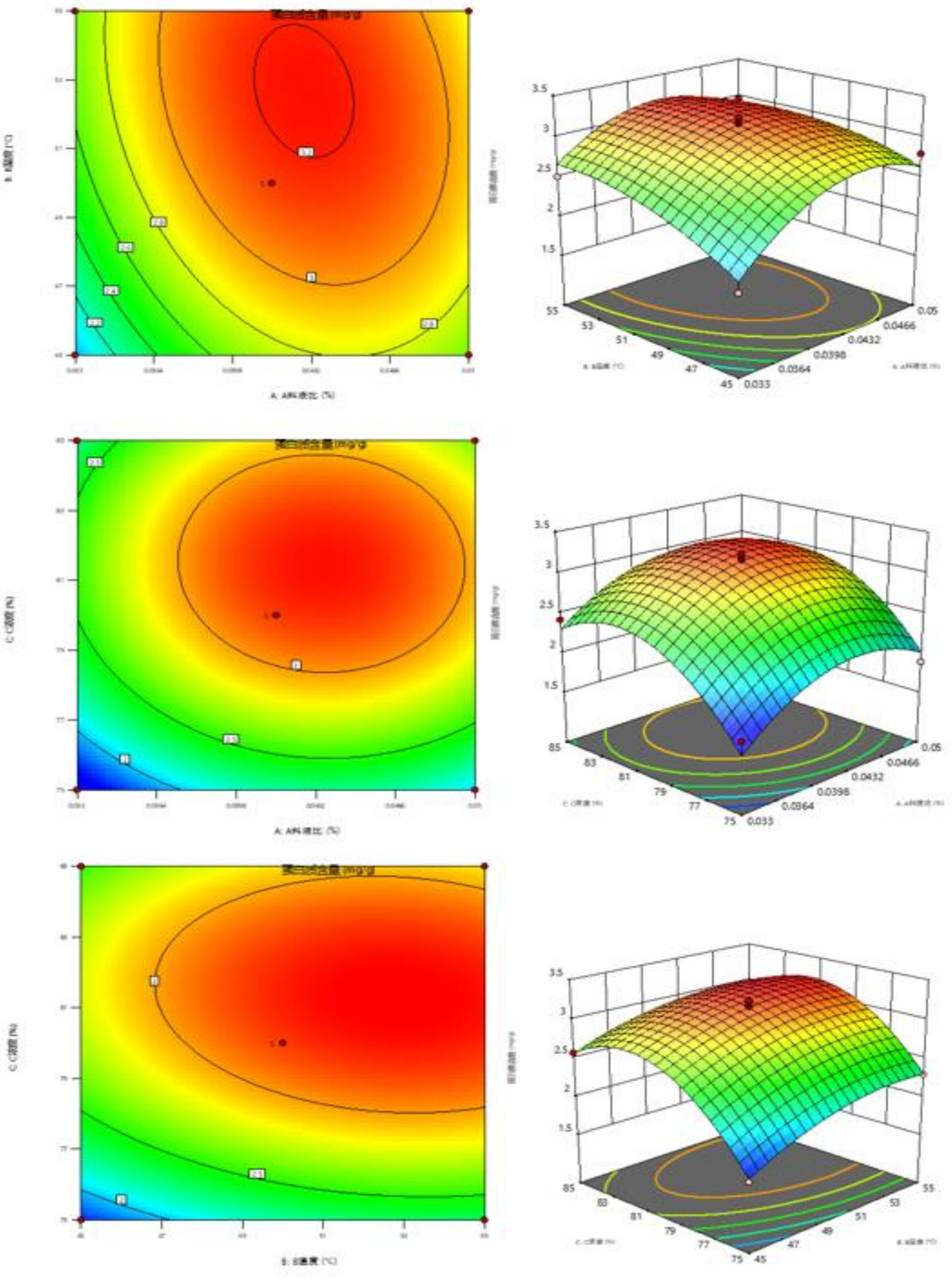

图 31 各两因素交互作用对地梢瓜果实可溶性硒蛋白含量影响的三维响应曲线图和等高线图

Fig.31 The three-dimensional response curve and contour map of the interaction of the two factors on the soluble selenoprotein content of the fruit of the CT fruit

3.8.5.6 验证实验

最佳工艺：料液比 1：23.3、提取温度 52.485 ℃、溶剂浓度 81.37 %理论硒蛋白含量为 3.271 mg/g。为方便实验的可操作性，在进行验证实验时设定工艺参数为：料液比 1：23、提取温度 52.5 ℃、溶剂浓度 81.4 %，实验硒蛋白含量为 3.268 mg/g 与理

中国知网 https://www.cnki.net

论预测值接近，表明此模型及提取工艺稳定性强，可操作性高，可以用于地梢瓜果实硒蛋白的提取。

表 25 验证实验结果

Table.25 Verify experimental results

	料液比（%）	温度（℃）	溶剂浓度（%）	硒蛋白含量（mg/g）
最佳工艺参数	1：23.3	52.485	81.37	3.271
实际工艺参数	1：23	52.5	81.4	3.268

3.9 讨论

水提法作为一种比较温和的提取方式，能够避免提取介质对蛋白质结构的破坏，较好的提取植物中所含的清蛋白类蛋白质。如果在提取时将 pH 值用适当的缓冲液控制，其稳定性和溶解度都可以增加。袁保红[65]对地木耳蛋白质的提取工艺及稳定性进行了研究，结果证明地木耳蛋白质可以采用水为溶剂的方法提取，提取最佳条件为 pH=9，温度为 40 ℃-50 ℃。提取时间控制在 1 h-2 h 即可达到 75 %的提取率。本论文通过单因素探究实验，发现地梢瓜可溶性硒蛋白含量随着提取时间的增加呈现先增高后减少的趋势，这是因为时间较低时，蛋白质只有少量溶出，随着时间增加，水溶性蛋白质逐渐溶解于提取液，但提取时间过长也会导致蛋白质结构受到破坏，导致产物得率下降。利用水提法提取地梢瓜硒蛋白的适宜温度较低，过高的温度会导致蛋白产物结构的破坏，影响最终提取率。料液比的变化对提取可溶性硒蛋白的提取率影响较为显著，地梢瓜果实提取液中可溶性硒蛋白含量随料液比的降低逐渐降低，这是由于较大的料液比提升了原料的表面积，从而使得提取效率较高，随着料液比逐渐降低，原料表面积降低，提取效率也受到了影响。

通过实验对响应面模型的验证，得出水提法提取地梢瓜硒蛋白的最佳工艺为：料液比 1：15.87、提取温度 28.64 ℃、时间 2.29 h 理论硒蛋白含量为 2.741 mg/g。

碱法提取的机理是：蛋白质分子中的二级结构与氢键被碱液破坏，导致其表面的电荷类型一致，加快了两者之间的解离[66]。致使蛋白分子的溶解度得到一定范围的提高。碱法提取蛋白质的具有简单的流程，且需要的投入也比较低，故使用碱提法进行蛋白质提取的研究较多，如林树花[67]以脱脂栝楼籽为材料，采用碱提法提取栝楼籽蛋白，通过正交试验对工艺参数进行了优化。结果表明，在提取时间 80 min，提取温度 70 ℃，固液比 1∶20，碱提 pH 值 10.0 的条件下，栝楼籽蛋白的平均得率为 44.37 %。但碱提法存在高碱条件下容易使蛋白质分子变形、水解及提取物中会存在非蛋白质等副作用。除此之外，高碱环境还会对蛋白质中的氨基酸构型造成破坏，从而降低蛋白的某些应用特性，严重的情况下会产生赖－丙氨酸等对人身体有损伤的物质。在使用碱提法提取地梢瓜硒蛋白时，提取率随时间增加而逐渐升高，在提取时间为 2h 时达到最高提取率，但提取时间继续增加，蛋白质的结构受到破坏程度越高，提取率随之下降。以温度作为变量时，条件 40 ℃在以下时，随着温度升高，分子运动加速，提取率逐渐提高，但当温度超过 40 ℃，部分蛋白质可能发生了一定程度的变性，故产物得率降低；以料液比作为变量时，较低的料液比会使得原料与提取介质的作用面积较小，影响提取效率，在料液比为 1：25 时达到最高值，这说明 1：25 可能是碱提法提取地梢瓜硒蛋白的适宜料液比。地梢瓜果实提取液中可溶性硒蛋白含量随 NaOH 浓度的增加逐渐增高，这说明碱浓度越高，对蛋白质二级结构与氢键的破坏作用越强，

中国知网 https://www.cnki.net

从而提升蛋白质的提取效率。通过分析响应面优化模型可以得知，料液比的作用系数要比其他因素的作用系数更大，故在各因素中，料液比对碱提法提取蛋白质的效率影响程度更大。

通过实验对响应面模型的验证，得出碱提法提取地梢瓜硒蛋白的最佳工艺为：料液比 1∶22.7、提取温度 44.62 ℃、溶剂浓度 0.243 mol/L 理论硒蛋白含量为 5.925 mg/g。

酸提法是利用蛋白质在强酸作用下的变性沉淀。利用 HCl 将提取液的 pH 值调节至 4.0 左右直至产生蛋白沉淀。此方法操作方便，节省能耗，成本低，沉淀快，可减少生物碱含量。目前已有部分研究利用酸提法进行蛋白质的提取，如程道梅[68]等利用酸提法甘薯叶片中的蛋白质效果较好，叶蛋白浓缩物得率为 19.5 %，而马洪鑫[69]等人以藜麦为原料，采用碱溶酸沉法、盐析法、酸提法、水提法 4 种方法制备藜麦蛋白时发现，酸提法所提蛋白表面光滑，结构紧密。这说明酸提法能够较好的保护所提取蛋白的整体结构。本节通过单因素实验发现，酸提法提取硒蛋白时，随着提取时间增加，硒蛋白得率提高，而当提取时间超过 1.5 h，这种增长趋势不再显著，这说明 1.5 h 可能是酸提法提取时间的适宜值。酸提法所需的适宜温度较高，在 30-50 ℃范围内，随着温度的升高，分子运动加速，提取液中硒蛋白含量逐渐升高。以料液比作为变量时，较低的料液比会使得原料与提取介质的作用面积较小，影响提取效率，酸提法提取地梢瓜硒蛋白在料液比为 1：30 时达到最高值，这说明 1：30 可能是碱提法提取地梢瓜硒蛋白的适宜料液比。以盐酸浓度为变量时，硒蛋白含量先随溶剂浓度的增高逐渐增高，在 0.2 mol/L 时达到最高值，随后提取率显著降低。这可能是由于随着盐酸浓度的逐渐提升，溶液的 pH 值逐渐接近地梢瓜蛋白质的等电点，使得蛋白质逐渐析出，但当盐酸浓度过高时，蛋白质的结构受到了破坏，提取率随之下降。

通过实验对响应面模型的验证，得出酸提法最佳工艺为料液比 1：28.6、提取温度 51.603 ℃、溶剂浓度 0.192 mol/L 理论硒蛋白含量为 2.762 mg/g。

盐提法的原理和碱提法基本相似，球蛋白类蛋白质能溶于稀盐溶液中，NaCl 溶液是典型的稀盐溶液，故盐提法提取蛋白质时常使用 NaCl 溶液作为提取介质。虽然盐提法的成本比较低，但是蛋白质在盐液中的溶解度没有在碱液中溶解度高，所以利用盐提法获得蛋白质的比率相对较低。但对于某些材料来说，盐提法提取蛋白质的效率更高，如李超等人采用盐提法提取花椒籽仁中蛋白质，在单因素试验的基础上，通过响应面试验优化花椒籽仁蛋白质提取最佳工艺条件。最佳提取工艺条件为提取 pH=11、提取时间 35 min、提取温度 50 ℃、料液比 1:21、NaCl 浓度 1.2 mol/L；在最佳提取条件下，花椒籽仁蛋白质提取率达到 88.77 %，所得蛋白质含量达到 93.17 %[70]。本节通过单因素实验探究发现，提取所需的最适时间为 1.5 h 左右，随后其提取率随时间的增加逐渐降低。与其他提取方法进行对比，发现盐提法达到最佳提取率所用的提取时间最短，这可能说明 NaCl 溶液促进球蛋白类蛋白质析出的速度要快于其他介质。盐提法所需的适宜温度要高于其他提取方法，在 30-50 ℃范围内，随着温度的升高，提取液中蛋白含量逐渐升高。根据响应面回归拟合模型，溶剂浓度对盐提法提取得率的影响最大，并且不同溶剂浓度对地梢瓜果实可溶性硒蛋白含量的所造成影响差异较为显著，这可能是由于蛋白质在较高浓度的盐液中的溶解度较高，但当盐液浓度继续升高时，蛋白质分子变形，甚至某些氨基酸的结构遭到破坏，使得蛋白提取率下降。

根据实验验证响应面优化结果，确定盐提法最佳工艺为：料液比 1：21.74、提取温度 35.99 ℃、溶剂浓度 0.141 mg/g，理论硒蛋白含量为 4.731 mg/g。

中国知网 https://www.cnki.net

有机溶剂提取法是利用醇溶蛋白在有机溶剂中的溶解度较大原理进行蛋白质提取的方法，因其提取效率不够理想，目前通常与其他方法进行联用以提升提取率。如李银清[71]采用醇提法制备鹿茸胶原蛋白，鹿茸醇提物中主要含有 I 型胶原含量约为 70 %，胶原蛋白提取率为 10.3 %。通过单因素比较实验发现，随着提取时间的增加，地梢瓜硒蛋白含量逐渐升高，并在 2.5 h 时达到最高值，这说明 2.5 h 可能是醇提法提取地梢瓜蛋白的适宜值，比对其他提取方法发现，有机溶剂提取法提取蛋白的适宜时间要比其他方法更长，这可能是由于醇溶蛋白较其它种类的蛋白更难溶解，需要更长的提取时间。以温度作为变量时，发现随着温度的升高，分子热运动加速，蛋白二级结构松动加速，提取的效率逐渐增加。以料液比作为变量时，醇提法所需的最佳料液比为 1:25 这与其他提取方法呈现出相似的趋势。以溶剂浓度作为变量时，随着溶剂浓度的增加，蛋白提取率逐渐增加这是由于随着溶剂浓度的提高，根据相似相溶原理，醇溶蛋白的溶解度逐渐提高故提取液中的蛋白含量逐渐升高。

通过实验对响应面模型的验证，得出有机溶剂提取法最佳工艺为：料液比 1:23.3、提取温度 52.485 ℃、溶剂浓度 81.37 %理论硒蛋白含量为 3.271 mg/g。

响应面法是利用合理的试验设计并通过得到的数据，采用多元二次回归方程来拟合因素与响应值之间的函数关系，然后对回归方程进行分析后，找到最佳工艺参数，是解决多变量问题的一种统计学方法。这种方法试验次数少、周期短最后得出的回归方程精度高，是降低成本、优化加工条件、提高产品质量的一种有效方法[72]。本章使用响应曲面法对五种地梢瓜硒蛋白提取方法进行了工艺优化并通过实验验证，优化后工艺蛋白最终提取率与模型理论值相近，并皆高于组内设置的单因素实验结果，证明了响应面模型的可靠性，得出了利用五种方法提取地梢瓜硒蛋白的最佳提取条件。通过对优化后各提取方法的硒蛋白提取率进行对比发现碱提法的提取效果最好。

中国知网 https://www.cnki.net

4 硒肥对地梢瓜果实硒含量及其体外抗氧化活性的影响

4.1 材料与方法

4.1.1 实验材料

在地梢瓜开花期叶面喷施氨基酸螯合硒肥，选择晴朗无风天气的傍晚对地梢瓜进行喷施有机硒肥，喷施效果以叶片均匀喷施但无液体滴下为宜，如施肥后遇下雨等天气则在第二天进行补施。设置 4 个处理，其有机硒肥浓度分别为：0 $mg \cdot L^{-1}$、处理 1：2 $mg \cdot L^{-1}$、处理 2：4 $mg \cdot L^{-1}$、处理 3：6 $mg \cdot L^{-1}$。每次间隔 7 天，共计喷施 4 次，在最后一次施肥 15 天后进行取样。

4.1.2 实验试剂

硒标准溶液（购自北方伟业计量技术研究院）、硝酸镍、超纯水、硝酸优级纯、过氧化氢、氢氧化钠、1，1-二苯基-2-三硝基苯肼（DPPH）自由基、无水乙醇、盐酸（优级纯）、三氯乙酸、三氯化铁、Tris-HCL 缓冲液（PH=8.2）、PBS 缓冲液（PH=6.6）、邻苯三酚、铁氰化钾、硫酸亚铁、过氧化氢、水杨酸。

4.1.3 实验仪器

SHZ-B 水浴恒温振荡器、容量瓶、INFINITE200PRO 型酶标仪、移液枪、HC-2518R 型高速冷冻离心机、MuItiwave GO 型微波消解仪、09A24S 型赶酸仪、ZA3000 型原子吸收分光光度计、ZX-S60 型全不锈钢恒温水浴锅。

4.1.4 实验方法

以 NaOH 为提取溶剂，设置提取条件为料液比 1：20、温度 45 ℃、0.15 mol/L、提取时间 2 h，水域恒温震荡提取，然后冷冻离心，取 2.5 mL 上清液于聚四乙烯消化罐中，加入 2.5 $mLHNO_3$ 和 0.5 mLH_2O_2，微波消解，赶酸，定容后置于 4 ℃冰箱保存备用。

4.2 硒肥处理果实及提取液中硒含量的测定

（1）果实硒含量

取地梢瓜果实粉末 0.5 g 置于聚四乙烯消化罐中，加入 5 $mLHNO_3$ 和 0.5 mLH_2O_2 在微波消解仪消解后采用石墨炉原子吸收法测得提取液中硒含量。

（2）提取液硒含量

取浸提液在 4000 r/min4 ℃下离心 15 min 后，取 2.5 mL 上清液于聚四乙烯消化罐中，加入 2.5 $mLHNO_3$ 和 0.5 mLH_2O_2 在微波消解仪消解后采用石墨炉原子吸收法测得提取液中硒含量。

（3）仪器设定参数

测定波长 196.0 nm，灯电流 7.0 mA，夹缝宽度 1.3 nm，时间常数 0.1 s,光电倍增管负高压 370 V，记录峰面积。采用与标准溶液共进基体改进剂方式加入 3 μL1%$Ni(NO_3)_2$,进样总量为 20 μL。

4.3 硒肥对地梢瓜果实硒蛋白体外抗氧化活性的影响

清除 DPPH 自由基能力的测定参考 DPPH 自由基清除法 [73]；还原力的测定参考铁氰化钾法[74]；清除羟自由基能力的测定参考水杨酸法[75]；清除超氧阴离子自由基能

中国知网 https://www.cnki.net

力的测定参考邻苯三酚法[76]。

4.4 数据处理方法

Microsoft Office Excel 2010 软件处理和作图，IBM SPSS Statistics 22 软件进行差异性分析。

4.5 结果与分析

4.5.1 不同浓度硒肥对果实及提取液中硒含量的影响

如图 33 所示，随着硒肥浓度的增高，果实和提取液的硒含量均呈先增高后降低的趋势，果实、提取液均在硒肥浓度为 2 ml/L 时硒含量最高分别为 1.92 mg/kg、0.58 mg/kg，显著高于其他处理（P＜0.05）。在硒肥浓度为 6 ml/L 时两者硒含量均最低，且低于对照组。因此，低浓度的硒肥处理可增加地梢瓜果实的硒含量，而浓度过高则会降低地梢瓜果实的硒含量。

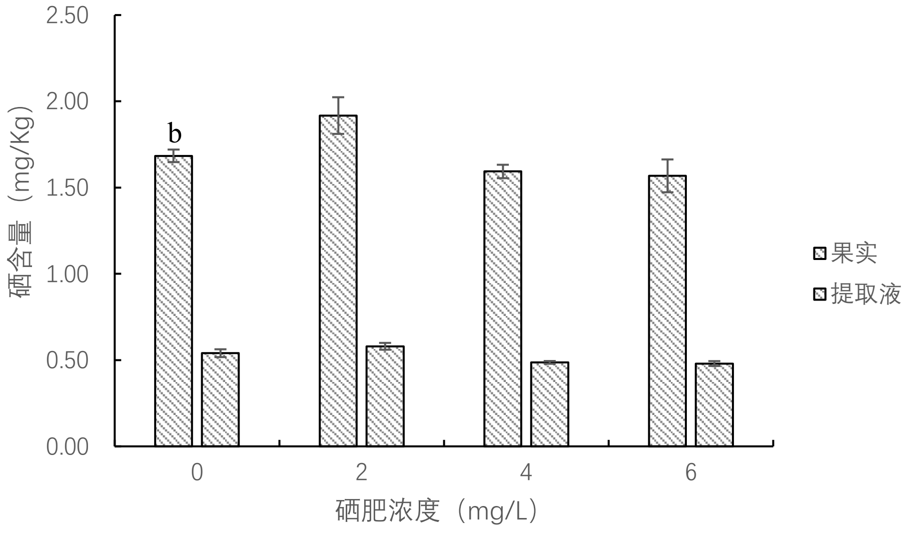

图 32 不同浓度硒肥处理果实及提取液中硒含量的影响

Fig.32 Effects of different concentrations of selenium fertilizer on selenium content in fruit and extract

注：不同小写字母表示不同硒肥处理果实间在 0.05 水平下的显著差异性；不同大写字母表示不同硒肥处理提取液间在 0.05 水平下的显著性差异。

4.5.2 清除 DPPH 效果分析

如图 34 可知，随着样品稀释浓度的增加，各施硒浓度地梢瓜硒蛋白提取液对 DPPH 自由基的清除效率均呈逐渐增加趋势。6 mg/L 硒肥处理的地梢瓜硒蛋白提取液清除率增长最快，从 42.28 %增长至61.94 %，增长了 19.66 %。随硒肥浓度的增加同 样品浓度水平的地梢瓜硒蛋白提取液清除率呈先增高后降低的趋势，2 mg/L 硒肥处 理的地梢瓜硒蛋白提取液清除能力最强，与提取液中硒含量的增长趋势一致。因此，
可以得出硒含量的高低与体外清除 DPPH 能力有一定的关联性。

中国知网 https://www.cnki.net

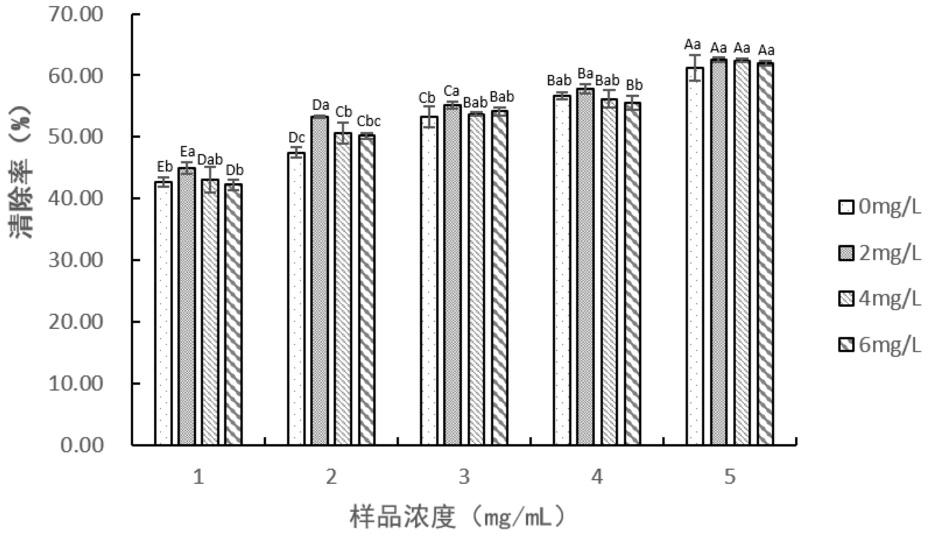

图 33 地稍瓜硒蛋白提取液体外清除 DPPH 自由基能力测定结果

Fig.33 The determination results of DPPH free radical scavenging ability of selenoprotein extract in CT in vitro

注：不同小写字母表示同一样品浓度不同硒肥浓度间在 0.05 水平下的显著差异性；不同大写字母表示同一硒肥浓度不同样品间在 0.05 水平下的显著性差异，下同。

4.5.3 还原能力分析

如图 35 可知，不同硒肥处理地稍瓜果实硒蛋白提取液的还原能力均随样品稀释浓度的增加不断增强，其中 2 mg/L 硒肥处理的地稍瓜果实硒蛋白提取液还原能力增长趋势最大，其吸光值从 0.37 增长至 1.33，增长达 0.96。2 mg/L 硒肥处理的地稍瓜果实硒蛋白提取液还原力均在同样品浓度中最高，与体外清除 DPPH 自由基能力一致。

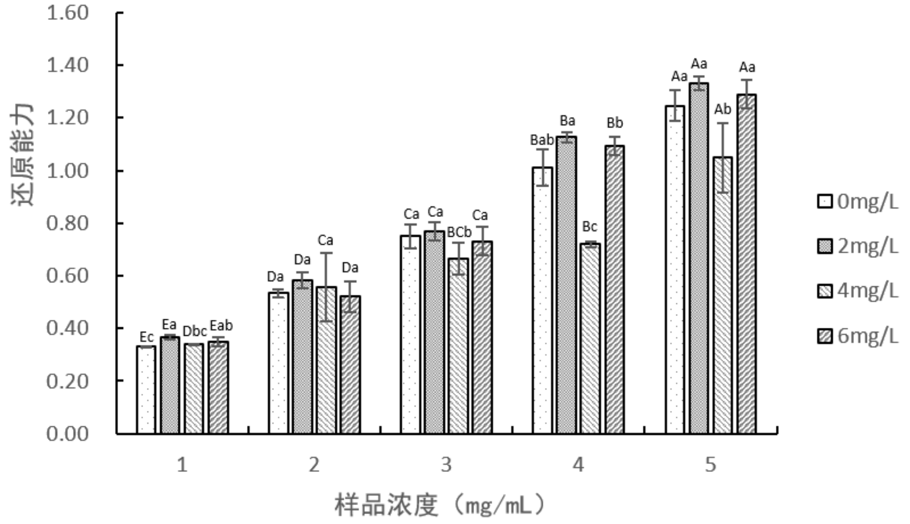

图 34 地稍瓜硒蛋白提取液还原能力测定结果

Fig.34 Determination results of reducing power of selenoprotein extract from CT

中国知网 https://www.cnki.net

4.5.4 清除羟自由基能力分析

如图 36 可知，不同硒肥处理地梢瓜果实硒蛋白提取液体外清除羟自由基的能力均随样品稀释浓度的增加不断增强，清除率增长值 2 mg/L 硒肥处理＞6 mg/L 硒肥处理＞0 mg/L 硒肥处理＞4 mg/L 硒肥处理。随硒肥浓度的增加同样品浓度水平的地梢瓜硒蛋白提取液清除率呈先增高后降低的趋势，2 mg/L 硒肥处理的地梢瓜硒蛋白提取液清除能力最强，清除率最高可达 68.73 %。

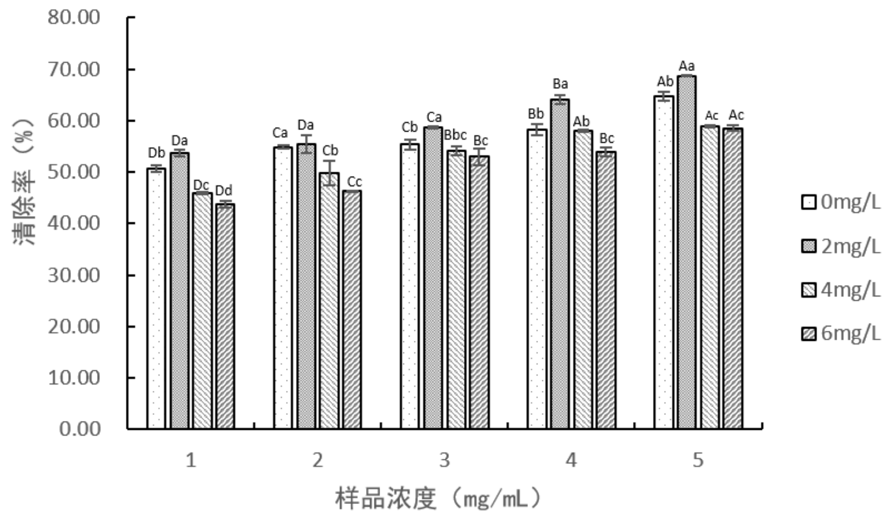

图 35 地梢瓜硒蛋白提取液体外清除羟自由基能力测定结果

Fig.35 Determination results of hydroxyl radical scavenging ability in vitro of selenoprotein extract of CT

4.5.5 清除超氧阴离子能力分析

如图 37 可知，随着样品稀释浓度的增加，各施硒浓度地梢瓜硒蛋白提取液对超氧阴离子的清除效率均呈逐渐增加趋势。4 mg/L 硒肥处理的地梢瓜硒蛋白提取液清除率增长最快，从 34.24 %增长至 51.15 %，增长了 16.91 %。随硒肥浓度的增加同样品浓度水平的地梢瓜硒蛋白提取液清除率呈先增高后降低的趋势，2 mg/L 硒肥处理的地梢瓜硒蛋白提取液清除能力最强，清除率最高可达 53.74 %。

中国知网 https://www.cnki.net

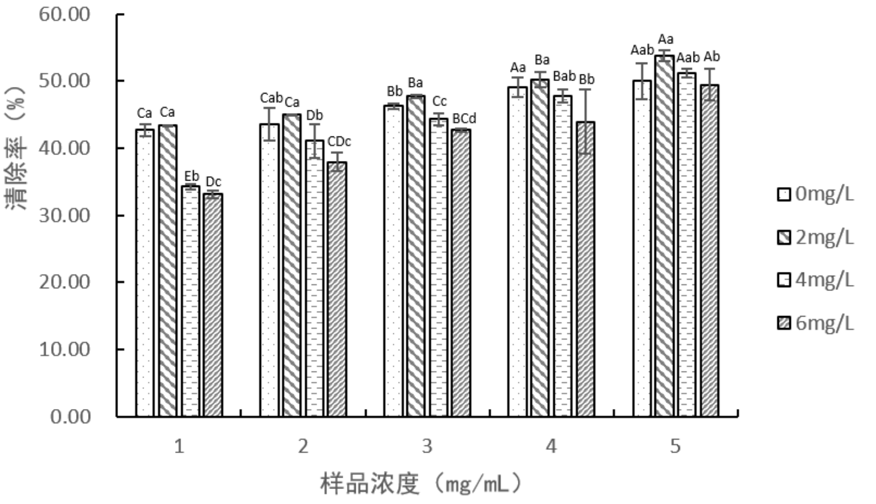

图 36 地梢瓜硒蛋白提取液体外清除超氧阴离子能力测定结果

Fig.36 The determination results of superoxide anion scavenging ability of selenoprotein extract in CT

4.6 讨论

硒是一种植物体内不可缺少的微量有益元素，在提高植物光合作用及抗氧化能力、调控植物重金属胁迫等方面具有重要作用，一定程度上还能增加作物产量[77-79]。长期以来，国内外就如何提高硒肥利用率，开展了大量的研究，在施硒方式上常采用土壤施硒、叶面喷施硒肥、硒肥浸种和拌种等[80]。其中以土壤施硒和叶面喷施硒肥为主。相关研究表明适量的硒元素可以提高谷子产量，而高浓度硒元素则会抑制产量[81-82]。在本实验中，不同浓度硒肥处理对地梢瓜果实硒含量具有明显的影响，地梢瓜果实硒含量随外源硒浓度的增加而呈现先升高再降低。这可能是因为适量施硒增加了土壤的有效硒含量，有利于土壤有效磷、钾含量的提高，改善土壤肥力，从而影响作物对养分的吸收利用而过量施硒可能会对土壤结构有一定的破坏[83]。

自由基是指最外层轨道上具有未成对电子的原子、原子团或分子，它们具有极高的反应活性，易于攻击周围的物质。抗氧化剂具有与生物大分子竞争结合自由基的能力，以此保护生命体内生物大分子不被自由基攻击，将自由基活性氧导向抗氧化分子，从而减缓有害效应的产生。由于体内实验方法周期长、成本大、实验繁琐，故大量样品的抗氧化性活性筛选仍有赖于体外实验法[84]。本论文对以碱提法最佳优化工艺提取出的不同浓度硒肥处理下的地梢瓜硒蛋白的抗氧化活性进行了体外实验探究，综合实验结果可以得出：地梢瓜硒蛋白具有较强的抗氧化活性，这与程利增对[85]硒蛋白的研究呈现出相似的结果。其各样品浓度的体外抗氧化性均高于李甜[74]测得同样品浓度的 BHT，但低于同样品浓度的天然抗氧化剂 Vc。这可能与硒蛋白组分中含有较高抗氧化活性的氨基酸有关，如段宝鑫[86]对富硒甘薯蛋白质的结构进行研究发现，其含有其中天冬氨酸、谷氨酸、亮氨酸等高抗氧化活性氨基酸，提高了富硒甘薯硒蛋白的抗氧化活性。

低浓度硒肥处理能够增强地梢瓜的还原能力以及其对 DPPH、羟自由基、超氧阴离子的清除能力。当处理浓度为 2 mg/L 时，地梢瓜硒蛋白的综合抗氧化活性较强，但当硒肥处理的浓度超过 4 mg/L 时，地梢瓜硒蛋白的抗氧化能力会随着浓度增高而发生不同程度的下降，这与不同浓度硒肥处理下地梢瓜中的硒元素含量的测定结果呈

中国知网 https://www.cnki.net

现一定的相关性。这可能是由于硒元素不仅能够作为构成硒蛋白和硒酶的活性中心而发挥抗氧化活性，其自身也作为一种带负电荷的非金属元素，能够催化或直接参与对植物体内过多的活性氧自由基的清除作用，硒肥处理浓度为 2 mg/L 地梢瓜的硒含量显著高于其他处理，故该浓度下硒蛋白的高抗氧化活性是硒元素和硒蛋白的协同作用结果。

中国知网 https://www.cnki.net

5 结论与展望

5.1 结论

(1)水分含量地梢瓜小果最高，为 90.01 %；维生素 C、可溶性总糖、可溶性蛋白含量雀瓢小果最高，分别 135.10 mg/100g、3.55 %、8.04 mg/g；粗纤维、粗脂肪、硒含量雀瓢大果最高，分别为 1.948 %、7.3 %、1.06 mg/kg。

(2)水提法最佳提取工艺为：料液比 1：15.87、提取温度 28.64 ℃、时间 2.29 h，理论硒蛋白含量为 2.741 mg/g；碱提法最佳提取工艺为：料液比 1：22.7、提取温度 44.62 ℃、溶剂浓度 0.243 mol/L，理论硒蛋白含量为 5.925 mg/g；酸提法最佳提取工艺为：料液比 1：28.6、提取温度 51.603 ℃、溶剂浓度 0.192 mol/L，理论硒蛋白含量为 2.762 mg/g；盐提法最佳提取工艺为：料液比 1：21.74、提取温度 35.99 ℃、溶剂浓度 0.141 mg/L，理论硒蛋白含量为 4.731 mg/g；有机溶剂提取法最佳提取工艺为：料液比 1:23.3、提取温度 52.485 ℃、溶剂浓度 81.37 %，理论硒蛋白含量为 3.271 mg/g。

(3)在硒肥施用浓度 2 mg/L 样品稀释浓度为 5 mg/mL 时，地梢瓜果实、提取液中硒含量最高分别为 1.92 mg/kg、0.58 mg/kg，此时地梢瓜果实硒蛋白清除 DPPH 自由基、羟自由基、超氧阴离子以及还原能力明显强于其他浓度处理。

5.2 展望

本研究主要针对地梢瓜果实的基础成分；硒蛋白的提取工艺；不同硒肥处理对果实提取液的硒含量以及体外抗氧化性进行了研究，由于实验条件和时间的限制，还存在以下内容需要进一步研究和讨论：

(1)地梢瓜果实硒蛋白的分离纯化、结构、有机硒形态等有待进一步研究。

(2)地梢瓜果实硒蛋白的产品性开发，以及开发以地梢瓜果实为原料的有机硒补充剂。

中国知网 https://www.cnki.net

参 考 文 献

[1] ZHU Y G,PILON-SMITS E A H,ZHAO F J, et al.Selenium in higher plants: understanding mechanisms for biofortification and phytoremediation[J]. Trends Plant Sci, 2009, 14(8) :436－44222

[2] 申玉香,陈银娣,张钰淇等.富硒桃的研究与开发综述[J].现代园艺,2022,45(10):17-19.

[3] 张澜,段耿婷,刘森林等.影响茶树硒积累的单因素及正交实验分析[J/OL].分子植物育种:1-15[2023-03-24]. http://kns.cnki.net/kcms/detail/46.1068.S.20230322.1117.007.html.

[4] Zhao Minmeng et al. The Effects of In Ovo Feeding of Selenized Glucose on Selenium Concentration and Antioxidant Capacity of Breast Muscle in Neonatal Broilers[J]. Biological trace element research, 2023,

[5] Qiu Huiling et al. Selenium-enriched Bacillus subtilis Improves Growth Performance, Antioxidant Capacity, Immune Status, and Gut Health of Broiler Chickens[J]. Biological trace element research, 2023,

[6] HAWKESFORD M J，ZHAO F J．Strategies for increasing the selenium content of wheat[J].J.Cereal Sci, 2007， 46(3):282－292．

[7] 梁郅哲,司振兴,牛慧伟,许自成,贾玮,许嘉阳.硒对植物的毒害作用研究进展[J].河南农业科学,2022,51(06):13-21.

[8] 郭肖兰.硒元素的生物强化途径研究进展[J].山西农业科学,2023,51(03):340-346.

[9] 贾宏昉,宋家永,王海红等.硒对作物生理、生长发育及产量、品质的影响研究进展[J].河南农业大学学报,2006(04):449-454.

[10] 刘大会,周文兵,朱端卫等.硒在植物中生理功能的研究进展[J].山地农业生物学报,2005(03):253-259.

[11] 李傲瑞,乔新星,赵飞飞,陈继平,张亚丽,任蕊.硒与人体健康关系研究进展[J].绿色科技,2020(12):121-122.

[12] 张勇.浅谈微量元素硒在人体中的作用[J].科学咨询(科技·管理),2011,254(08):76-77.

[13] 关玉群,朱磊,王充,胡锡珉,代卫红.硒与人体健康[J].现代预防医学,2003(05):700-702.

[14] 张剑.食品中硒的功效与安全性分析[J].食品安全导刊,2018, 213(22):56-57.

[15] 陈长兰,郇丰宁,孟雪莲,吕晶.硒对人体的作用机理及科学补硒方法[J].辽宁大学学报(自然科学版),2016,43(02):155-168+92.

[16] 田俊梅,张丁,付瑞娟等.大豆硒蛋白与亚硒酸钠生物利用的比较研究[J].大豆科学,2010,29(03):534-536.

[17] 程建中,杨萍,桂仁意.植物硒形态分析的研究综述[J].浙江农林大学学报,2012,29(02):288-295.

[18] 李应生,李亚男,陈大清.硒的生物学功能及植物的富硒机理[J].湖北农学院学报,2003(06):476-480.

[19] 刘为,尹金晶,吴慕慈等.富硒农产品中硒代氨基酸形态及其在不同蛋白组分中的分布[J].食品与机械,2022,38(06):45-51+190.

[20] Hu Jiangtao et al. Seleno-Amino Acids in Vegetables: A Review of Their Forms and Metabolism [J]. Frontiers in Plant Science, 2022, 13 : 804368-804368.

[21] 陈永波,刘淑琴,刘瑶等.富硒产品中硒的形态分析及化学评分模式的建立[J].生物资源,2021,43(01):79-85.

[22] 刘成龙,田宗仁,张克等.硒蛋白生理功能研究综述[J].中国食品学报,2021,21(02):358-366.

[23] 则拉莱·司玛依,帕尔哈提·柔孜,吾哈丽妮萨·麦麦提托合提等.三种甘草种子蛋白的提取方法、结构及抗氧化活性比较研究[J].食品与发酵工业,2022,48(18):227-234.

[24] 冯谈林,杨秦玉,韩淑雅等.苦菜硒蛋白饲料添加剂制备及抗氧化性研究[J].中国饲料,2023,719(03):116-123.

[25] 秦之皓. 富硒平菇蛋白的硒检测[D]. 武汉：武汉轻工大学,2020.

中国知网 https://www.cnki.net

[26] 马洪鑫,袁治浩,刘洪海等.比较不同方法提取藜麦蛋白[J].食品安全质量检测学报,2021,12(05):1890-1898.

[27] 杨柳,尤丽新,张英楠.有机溶剂法提取玉米蛋白的研究[J].吉林农业,2015,346(01):59.

[28] 郭嘉,门小明,邓波等.动物硒蛋白功能、表达及其肉质调控机制研究进展[J].浙江农业学报,2021,33(09):1779-1788.

[29] 王连顺,李梓萌,靳晓敏等.硒和硒蛋白在水产养殖中的应用进展[J].动物营养学报,2023,35(01):77-85.

[30] 陈春英,张劲松,黄开勋等.烟叶硒蛋白在实验性小鼠肝损伤中的抗氧化作用[J].营养学报,1996(04):457-460.

[31] 吴春凤,仲维杰,吴海双等.硒对氧化应激诱导的胎盘滋养层细胞增殖与凋亡的影响[J].现代生物医学进展,2020,20(23):4413-4417.

[32] 铁梅,刘丽,庄晓虹等.硒蛋白和过氧化氢酶清除羟自由基作用的研究[J].食品研究与开发,2017,38(17):6-10.

[33] 张晓艳,杨忠仁,张凤兰,郝丽珍,翟学婧,唐雨.干旱胁迫对地梢瓜幼苗叶片解剖结构的影响[J].东北师大学报(自然科学版),2020,52(03):116-123.

[34] 陈叶,梁军,罗光宏.水土保持植物地梢瓜驯化研究初探[J].林业实用技术,2008(02):35-36.

[35] 翟学婧. 地梢瓜胚胎生物学研究[D]. 呼和浩特：内蒙古农业大学,2018.

[36] 刘贺群,王忠生.地梢瓜治疗肛周尖锐湿疣报告[C].中国肛肠病研究心得集,2011:375.

[37] 苑辉卿,左春旭.地梢瓜化学成分的研究[J].药学学报,1992(08):589-594.

[38] 王玎,陈刚,乔莉等.地梢瓜果实化学成分的研究[J].中国药物化学杂志,2007(02):101-103.

[39] 张晓艳,杨忠仁,张凤兰等.干旱胁迫对地梢瓜琥珀酸合成代谢的影响[J].西北农林科技大学学报(自然科学版),2020,48(04):137-145.

[40] 喜杰,吴依静,钟国跃等.基于抗炎活性的蒙药地梢瓜质量标准指标性成分测定[J].中华中医药杂志,2022,37(02):1046-1050

[41] 席存瑞,樊旭红,樊友丽,等.食药饲多功用野生植物地梢瓜的驯化栽培[J].农业开发与装备,2020(01):126-127+153.

[42] 马玖军,崔新国,王小龙等.地稍瓜的利用和栽培[J].特种经济动植物,2006(03):30.

[43] 王爱文,罗光宏,陈叶.药食两用野菜—地稍瓜的特征特性及人工栽培技术[J].甘肃农业科技,2006(11):30-31.

[44] 董昕瑜.地稍瓜栽培管理[J].特种经济动植物,2017,20(12):46-47.

[45] 丁梦军,郝丽珍,那顺吉日嘎啦,张凤兰,杨忠仁,张晓艳,布仁吉雅.内蒙古七种饲用植物的营养成分分析[J].黑龙江畜牧兽医,2017,(04):165-167.

[46] 金世超. 萝藦科三种能源植物脂溶性成分提取与转化利用研究[D]. 北京：北京林业大学,2014.

[47] 林敏,吴冬青,安红钢等.FAAS 测定地梢瓜不同部位金属元素[J].光谱实验室,2009,26(06):1477-1479.

[48] 曹乌吉斯古楞. 内蒙古野生蔬菜资源及其综合评价[D]. 呼和浩特：内蒙古师范大学,2007.

[49] 王晓华,李桂凤.山东省 50 种常食野生植物硒含量分析[J].营养学报,2003(02):171-172+174.

[50] 刘其容.富硒农业种植发展前景综述[J].农民致富之友,2019, 595(02):108.

[51] 李小方，张志良主编.植物生理学实验指导第 5 版[M].北京：高等教育出版社,2016

[52] 马宏飞,卢生有,韩秋菊,李薇.紫外分光光度法测定五种果蔬中维生素 C 的含量[J].化学与生物工程,2012,29(08):92-94.

[53] 高俊凤主编.植物生理学实验指导[M].北京：高等教育出版社,2006

中国知网 https://www.cnki.net

[54] 李宝瑞. 黄豆富硒特性及硒的形态分布研究[D]. 沈阳：辽宁大学,2015.

[55] MCNULTY S N,TORT J F, RINALDI G,et al.Genomes of Fasciola hepatica from the Americas Reveal Colonization with Neorickettsia Endobacteria Related to the Agents of Potomac Horse and Human Sennetsu Fevers[J].PLoS Genetics, 2017, 13(1):e1006537

[56] CLARK P E, LEE J.KO K.et al.Prescribed fire effects on resource selection by cattle in mesic sagebrush steppe.Part 2:Mid-summer grazing [J].Journal of Arid Environments, 2016, 124(1):398-412

[57] 王春林,张博,贾学明.子午岭林区常见野生蔬菜营养成分分析[J].长江蔬菜,2015(02):46-49.

[58] 张凤兰,杨忠仁,郝丽珍,刘建文,胡宁宝,张进文.5 种野生蔬菜叶片营养成分分析[J].华北农学报,2009,24(02):164-169.

[59] 杨诗谣,李美英,宋正蕊.不同蔬菜水果中维生素 C(VC)含量检测分析[J].中国检验检测,2020,28(02):38-39+65.

[60] 仇亮,程玉静,翟彩娇,王小秋,宋益民,姜永平,邱海荣.不同品种蘘荷营养成分分析和综合评价[J].浙江农业科学,2022,63(05):1060-1063+1067.

[61] 刘志皋主编.食品营养学第 2 版[M].北京：中国轻工业出版社,2004

[62] 杨月欣.中国食物成分表[M].北京：北京大学医学出版社,2019

[63] 喻凤莲,周娟娇,侯莉等.德阳市富硒土壤区富硒蔬菜种植适宜性筛选研究[J].四川农业科技,2022,423(12):40-43.

[64] 宋东杰,吴晓霞,周丽丽,陆亚琴,冯亭.方山部分野菜资源植物营养成分分析[J].北方园艺,2012(01):52-54.

[65] 袁保红,杜青平.地木耳蛋白质提取工艺及其稳定性的研究[J].食品研究与开发,2007(04):117-120.

[66] 江漓，林姚，赵小虎,等.除杂和酶法相结合提取米渣蛋白[J]. 食品科技，2011(12):179-182.

[67] 林树花,谭兴和,李高阳等.碱提法提取栝楼籽蛋白工艺条件的优化[J].湖南农业科学,2015,353(02):86-88.

[68] 程道梅,韩珍琼.甘薯叶蛋白提取方法对提取率及营养成分的影响研究[J].食品科技,2010,35(11):208-210.

[69] 马洪鑫,袁治浩,刘洪海,杨许花,杨雪研,柯义强,高丹丹,宋礼.比较不同方法提取藜麦蛋白[J].食品安全质量检测学报,2021,12(05):1890-1898.

[70] 李超,蒲彪,刘兴艳等.响应面法优化花椒籽仁蛋白质盐提工艺条件[J].中国油脂,2017,42(06):97-101+120.

[71] 李银清,毕胜男,韩烨等.醇提法制备鹿茸胶原的初步研究[J].特产研究,2007,115(01):9-11.

[72] 慕运动.响应面方法及其在食品工业中的应用[J].郑州工程学院学报,2001(03):91-94.

[73] 熊双丽,卢飞,史敏娟等.DPPH 自由基清除活性评价方法在抗氧化剂筛选中的研究进展[J].食品工业科技,2012,33(08):380-383.

[74] 李甜. 新春 36 号黑小麦中硒的分布、富硒蛋白的制备及抗氧化活性研究[D]. 石河子：石河子大学,2016.

[75] 李粉玲,蔡汉权,林泽平.红豆多糖抗氧化性及还原能力的研究[J].食品工业,2014,35(02):190-194.

[76] 宋怡红. 富硒香菇中有机硒形态及其特性研究[D]. 沈阳：辽宁大学,2022.

[77] 王晓虎,张建珍,侯非凡等.土施硒肥位置对谷子生理特性及硒积累的影响[J/OL].山西农业大学学报(自然科学版):1-9[2023-04-14].

[78] 马俊桃,周文,李静浩等.外源硒调控植物重金属胁迫机制的研究进展[J]. 中国农业科技导报，2022, 24（6）：27-35.

[79] 兰敏.外源硒对谷子光合特性、产量形成及硒吸收的影响[D]. 晋中：山西农业大学,2020.

中国知网 https://www.cnki.net

[80] 李星星，韩芳，周雪，等.富硒谷子研究进展[J].中国农学通报,2022，38（7）：1-6.
[81] 郭美俊,郭平毅,原向阳等.叶面喷施亚硒酸钠对谷子光合特性及产量构成的影响[J].核农学报,2014,28(06):1099-1107.
[82] 张宇杰,郭平毅,郭美俊等.外源硒矿粉对谷子保护酶活性、产量和籽粒中硒含量的影响[J].中国农业科技导报,2021,23(05):153-159.
[83] 田秀英,李会合,王正银.施硒对苦荞 N,P,K 营养元素和土壤有效养分含量的影响[J].水土保持学报,2009,23(03):112-115.
[84] 于守洋,崔洪斌.中国保健食品的进展[M]. 北京:人民卫生出版社,2001.96
[85] 程利增. 茶叶硒蛋白的分离纯化、结构、有机硒形态及其抗氧化活性研究[D]. 上海：上海师范大学,2017.
[86] 段宝鑫. 富硒甘薯中蛋白质的提取、纯化及其结构的初步解析[D]. 沈阳：辽宁大学,2021.
中国知网 https://www.cnki.net
